# Engineering Azobenzene Derivatives to Control the Photoisomerization Process

**DOI:** 10.1021/acs.jpca.3c06108

**Published:** 2023-12-05

**Authors:** Flavia Aleotti, Vasilis Petropoulos, Hannah Van Overeem, Michele Pettini, Michele Mancinelli, Daniel Pecorari, Margherita Maiuri, Riccardo Medri, Andrea Mazzanti, Fabrizio Preda, Antonio Perri, Dario Polli, Irene Conti, Giulio Cerullo, Marco Garavelli

**Affiliations:** † Dipartimento di Chimica Industriale “Toso Montanari”, Università di Bologna, Viale del Risorgimento 4, 40136 Bologna, Italy; ‡ Dipartimento di Fisica - Politecnico di Milano, Piazza Leonardo da Vinci 32, Milano 20133, Italy; § van’t Hoff Institute for Molecular Sciences, Universiteit van Amsterdam, Science Park 904, 1098 XH Amsterdam, The Netherlands; ∥ Dipartimento di Chimica “Giacomo Ciamician”, Università di Bologna, Via F. Selmi 2, 40126 Bologna, Italy; ⊥ NIREOS s.r.l, Via Giovanni Durando 39, 20158 Milan, Italy; # CNR - Institute for Photonics and Nanotechnologies (IFN), Piazza Leonardo da Vinci 32, 20133 Milan, Italy

## Abstract

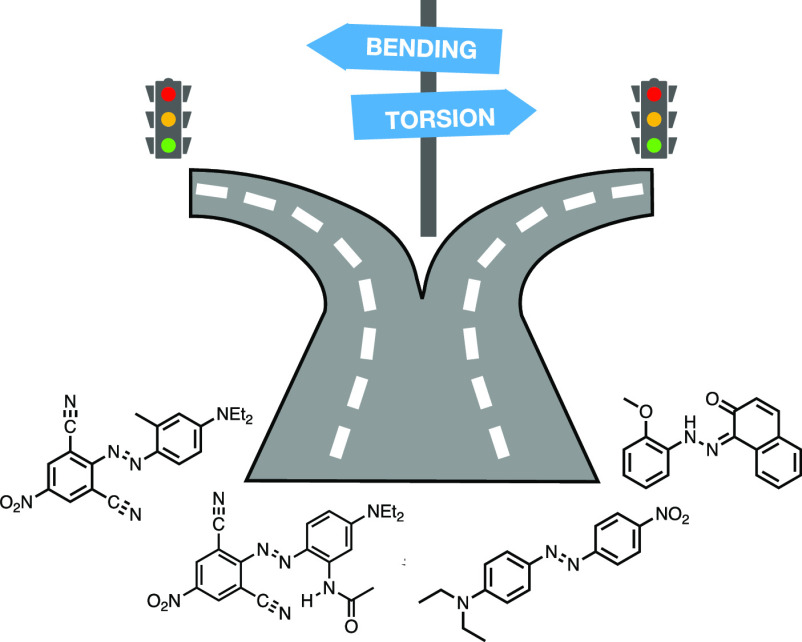

In this work, we show how the structural features of photoactive azobenzene derivatives can influence the photoexcited state behavior and the yield of the trans/cis photoisomerization process. By combining high-resolution transient absorption experiments in the vis–NIR region and quantum chemistry calculations (TDDFT and RASPT2), we address the origin of the transient signals of three poly-substituted push–pull azobenzenes with an increasing strength of the intramolecular interactions stabilizing the planar trans isomer (absence of intramolecular H-bonds, methyl, and traditional H-bond, respectively, for 4-diethyl-4′-nitroazobenzene, Disperse Blue 366, and Disperse Blue 165) and a commercial red dye showing keto–enol tautomerism involving the azo group (Sudan Red G). Our results indicate that the intramolecular H-bonds can act as a “molecular lock” stabilizing the trans isomer and increasing the energy barrier along the photoreactive CNNC torsion coordinate, thus preventing photoisomerization in the Disperse Blue dyes. In contrast, the involvement of the azo group in keto–enol tautomerism can be employed as a strategy to change the nature of the lower excited state and remove the nonproductive symmetric CNN/NNC bending pathway typical of the azo group, thus favoring the productive torsional motion. Taken together, our results can provide guidelines for the structural design of azobenzene-based photoswitches with a tunable excited state behavior.

## 1 Introduction

Azobenzene is a prototypical photoactive molecule that undergoes trans ⇆ cis photoisomerization upon light irradiation. This photoswitchable behavior makes azobenzene attractive for applications in materials with light controllable properties,^1−5^ but applications in photobiology and photopharmacology have also grown in recent years.^6−12^ Already in the late 1960s, azobenzene was applied to the photocontrol of enzymes and ion channels,^13,14^ and nowadays, targeted protein modification with azobenzenes has led to many more in vivo applications.^7,8^ Traditionally, trans → cis conversion is achieved with UV-light irradiation, whereas the reverse cis → trans process can occur via either thermal relaxation or visible-light irradiation. For in vivo applications, however, photoswitching in the vis–NIR window is desirable to avoid cell damage and enable effective tissue penetration. Even though progresses were made thanks to two-photon absorption^15,16^ and upconverting nanoparticles,^17^ there are still practical and technological limitations for these strategies that make it desirable to find photoswitches that undergo photoisomerization with vis–NIR light.

To this aim, push–pull substitution has shown to redshift the excitation energy of the absorbing bright state in azobenzene (ππ*) up to the visible range^18−22^ thanks to the concurrent HOMO destabilization (by the electron donor group) and LUMO stabilization (by the electron withdrawing group). Although push–pull azobenzenes often show cis instability,^22^ the high rate of thermal back-isomerization is desirable for some photopharmacological applications, because it allows the reversion of the switch by simply stopping the irradiation.^9^ In addition to the spectral differences, it has been recently suggested that push–pull substitution is also influencing the deactivation mechanism in azobenzene, with possible consequences on the photoisomerization quantum yield.^23^

Over the years, two reactive coordinates have been identified for the parent compound:^24−28^ the rotation of one phenyl around the central NN bond (torsion) and the oscillations of the two CNN angles (bending). The latter is activated immediately after photoexcitation to the bright ππ* state (S_2_) and promotes ultrafast internal conversion (IC) to the dark *n*π* state (S_1_). In contrast, the torsional motion requires internal vibrational energy redistribution and is responsible for the photoisomerization on S_1_.^24,25,27^ Nowadays, there is general consensus that bending and torsion are not mutually exclusive but rather mixed to form a wide S_1_/S_0_ crossing seam that drives the photoprocess. However, the balance between the two mechanisms (i.e., which region of the crossing seam is visited) has turned out to be a key factor in determining the photoisomerization quantum yield. Indeed, the bending oscillations triggered by light absorption are mainly symmetric and prevent isomerization because they could lead at most to the inversion of both CNN angles (and therefore to the starting isomer). On the other hand, the torsional region of the CI seam is more favorable to isomerization. The lower ππ* excitation energy in push–pull azobenzenes reduces the amplitude of the symmetric CNN oscillations compared to the parent compound, thus favoring the energy redistribution to the (productive) torsional coordinate.^23^

Here, we present a joint experimental/computational study of four push–pull azobenzene derivatives selected for their absorption in the visible range, which are of interest for biological applications. By means of vis–NIR, high-temporal-resolution transient absorption (TA) spectroscopy, and electronic structure calculations (both at the TDDFT and RASPT2 level), we investigate their excited state behavior and connect the structural differences with different deactivation mechanisms. A prototypical push–pull azobenzene (4-diethyl-4′-nitroazobenzene, DNAB) is compared to a red dye showing keto–enol tautomerism involving the azo group (Sudan Red G, SRG) and two poly-substituted blue dyes (Disperse Blue 366 named DB366 and Disperse Blue 165 named DB165) that show an increasing hydrogen bond strength (methyl vs traditional H-bond). For each compound, the origin of the transient signals is first discussed based on the electronic structure at key geometries (e.g., ground and excited state minima). Subsequently, the presence and accessibility of crossing points along the deactivation pathways typical of azo compounds are discussed. Understanding the effect of the different substituents (and of their position) on the excited state behavior is of key importance for the rational design of azobenzene derivatives with tunable excitation energy and photoisomerization efficiency.

## 2 Materials and Methods

### 2.1 Synthesis and Purification of the Dyes

SRG and DNAB are yellowish-red azo dyes commercially available and were used as received. Details on the synthesis, purification, and characterization of DB366 and DB165 are found in the Supporting Information.

### 2.2 Steady-State Absorption

UV–vis–NIR absorption measurements were recorded by using a PerkinElmer Lambda 1050 spectrophotometer. The measurements were carried out in air-equilibrated solutions at 25 °C. Absorption spectra were measured in 1 mm path length quartz cells, after subtracting the contribution of the reference solvent.

### 2.3 Steady-State Fluorescence

Steady-state photoluminescence measurements were performed with excitation from a supercontinuum laser in the visible area using a short-pass filter at 500 nm (FESH500). The emission was recorded using a commercial ultrastable common-path birefringent interferometer (model GEMINI by NIREOS SRL) based on Fourier transform spectroscopy.^29^ The measurements were carried out in 1 cm path length quartz cells after subtracting the contribution of the reference solvent. All fluorescence measurements were recorded with a 0.5 s integration time. For determination of the fluorescence quantum yield, rhodamine B in water was used as a reference (details in the Supporting Information).

### 2.4 Transient Absorption Spectroscopy

The femtosecond TA experiments were conducted using an amplified Ti:sapphire laser system, which emits ≈100 fs pulses with a central wavelength of 800 nm (1.55 eV) and a repetition rate of 2 kHz.^30,31^ The pump beam was delivered by a noncollinear optical parametric amplifier (NOPA) pumped by the second harmonic of the laser, generated in a 1 mm thick β-barium borate (BBO) crystal and seeded by a white-light continuum (WLC) generated in a 1 mm thick sapphire plate. The broadband amplified pulses had a spectrum spanning from 1.85 to 2.43 eV and were compressed to sub-20 fs duration using a pair of specially designed chirped mirrors. The pump pulses were modulated at a 1 kHz repetition rate via a mechanical chopper and focused onto a spot with a diameter of 180 μm. A fraction of the 800 nm laser beam was employed to generate the probe pulses by supercontinuum generation in a 1 mm thick sapphire plate. The probe’s energy ranged from 1.7 to 2.53 eV. The probe beam was focused onto a spot with a diameter of 110 μm and positioned at a noncollinear angle relative to the pump beam on the samples. The overall temporal resolution of the setup was assessed using cross-frequency resolved optical gating (X-FROG) and found to be less than 30 fs.^30^ For DNAB and SRG, experiments aimed at examining long-lived residual signals were performed, using narrowband NOPA excitation pulses at 2.38 and 1.72–2.82 eV probe energy bandwidth, resulting in a sub-100 fs temporal resolution. In all the experiments, the temporal delay between the pump and probe pulses could be adjusted with a mechanical stage, allowing for a maximum pump–probe delay time of 40 ps and 1.2 ns in the broadband and narrowband excitation experiments, respectively. The samples used were prepared in 200 μm thick quartz cuvettes in a methanol solvent with an absorbance of less than 0.1 OD at the peak wavelength. To prevent photodamage or photochemistry in the illuminated area, the samples were flowed during the measurements. The excitation fluence was maintained at approximately 30 μJ/cm^2^ for all experiments. A magic angle configuration (54.7°) between the pump and probe polarizations was employed for all femtosecond TA experiments. Global analysis by multiexponential functions was performed on the data sets using the Glotaran software.^32^

## 3 Computational Methods

### 3.1 Preliminary Calculations

In order to find out the most relevant geometries to consider for the subsequent QM/MM studies, some preliminary calculations were performed for all the considered chromophores either in the gas phase or with implicit solvation (PCM, methanol; details are found in the Supporting Information).

### 3.2 QM/MM Setup

The setup of the methanol droplets containing the various chromophores was accomplished in three steps using the automated tools implemented in COBRAMM^33^ using the following parameters. Step 1: for each compound, the gas-phase optimized geometry (M06/6-31G*) was put in a methanol box of size 20 Å with periodic boundary conditions. Step 2: the solvent molecules were subject to an MM optimization followed by a thermalization molecular dynamics (MD) run of 30 ps at constant volume to heat the system up to 300 K and a subsequent 1500 ps equilibration MD at constant temperature (300 K). All these simulations were run using the GAFF force field through the interface with AMBER18^34^ using a time step of 0.002 ps, a cutoff value for the evaluation of the MM potential of 10 Å, and replacing the MM charges of the solute atoms with the M06/6-31G* ESP charges. Step 3: a 16 Å droplet of solvent molecules surrounding the chromophore was cut out of the lowest energy snapshot from the equilibration trajectory. The chromophore was either subject to a modified force field or kept fixed at the gas-phase optimized geometry during all of the droplet setup (see the Supporting Information for details).

### 3.3 QM/MM Calculations

All the QM/MM calculations were performed using COBRAMM^33^ interfaced with the MM software AMBER18^34^ and with Gaussian16^35^ or OpenMolcas^36^ for the (TD)DFT and RASSCF/RASPT2 QM calculations, respectively. The QM system encompassed all the chromophore atoms, while the solvent molecules were treated as the MM part of which the inner 8 Å layer was allowed to move during geometry optimizations and molecular dynamics (movable layer) while molecules in the outer solvation shell (8–16 Å) were kept frozen (at their position at the time of the droplet cut) throughout all QM/MM computations.

All the QM calculations for geometry optimizations and molecular dynamics were performed at the DFT (ground state) or TDDFT (excited states) in the presence of the MM external charges using the M06 functional and the 6-31G* basis set and applying Grimme’s dispersion correction with the original D3 damping function (GD3). In the case of excited state TDDFT molecular dynamics, the Tamm–Dancoff approximation (TDA) was applied (as the COBRAMM default). The electronic state energies at the optimized geometries, as well as at state crossing points identified by MD, were recalculated at the multistate (MS) and/or single-state (SS) RASPT2/RASSCF/6-31G* level of theory (see the Supporting Information for RASPT2 details and RASSCF active space size and composition). For the evaluation of excited-state absorption brightness at the TDDFT level, transition dipole moments (TDMs) were evaluated using the TDA eigenvector components through the Multiwfn program.^37^ In the case of Sudan Red G, the spin–orbit coupling (SOC) magnitude at the TDDFT level was also evaluated from the eigenvector components using the pySOC code.^38^ In the cases where the QM/MM energies were not comparable between different geometries (due to too different droplet configurations), the reported relative energies were obtained by adding the QM/MM energy gaps (with respect to S_0_) to the energy of S_0_ in the polarizable continuum model (PCM), relative to the S_0_ minimum in PCM. Such cases are explicitly mentioned in the following.

### 3.4 PCM Calculations

All calculations with implicit solvation were performed at the M06/6-31G* level of theory through Gaussian16^35^ employing the PCM model (methanol). Two types of calculations were performed for each considered compound in order to explore the potential energy surfaces (PES) and assess the presence of the crossing points already reported for unsubstituted azobenzene along CNNC torsion and symmetric CNN/NNC bending^24,26−28^: (a) a fully unconstrained geometry optimization starting from a distorted geometry along CNNC torsion, in order to break the planarity of the trans system and possibly locate the peaked S_1_/S_0_ torsional crossing; (b) a relaxed scan along the CNN/NNC symmetric bending coordinate starting from the Franck–Condon (FC) point in order to locate a possible sloped S_1_/S_0_ planar crossing.

## 4 Results and Discussion

**Figure 1:**
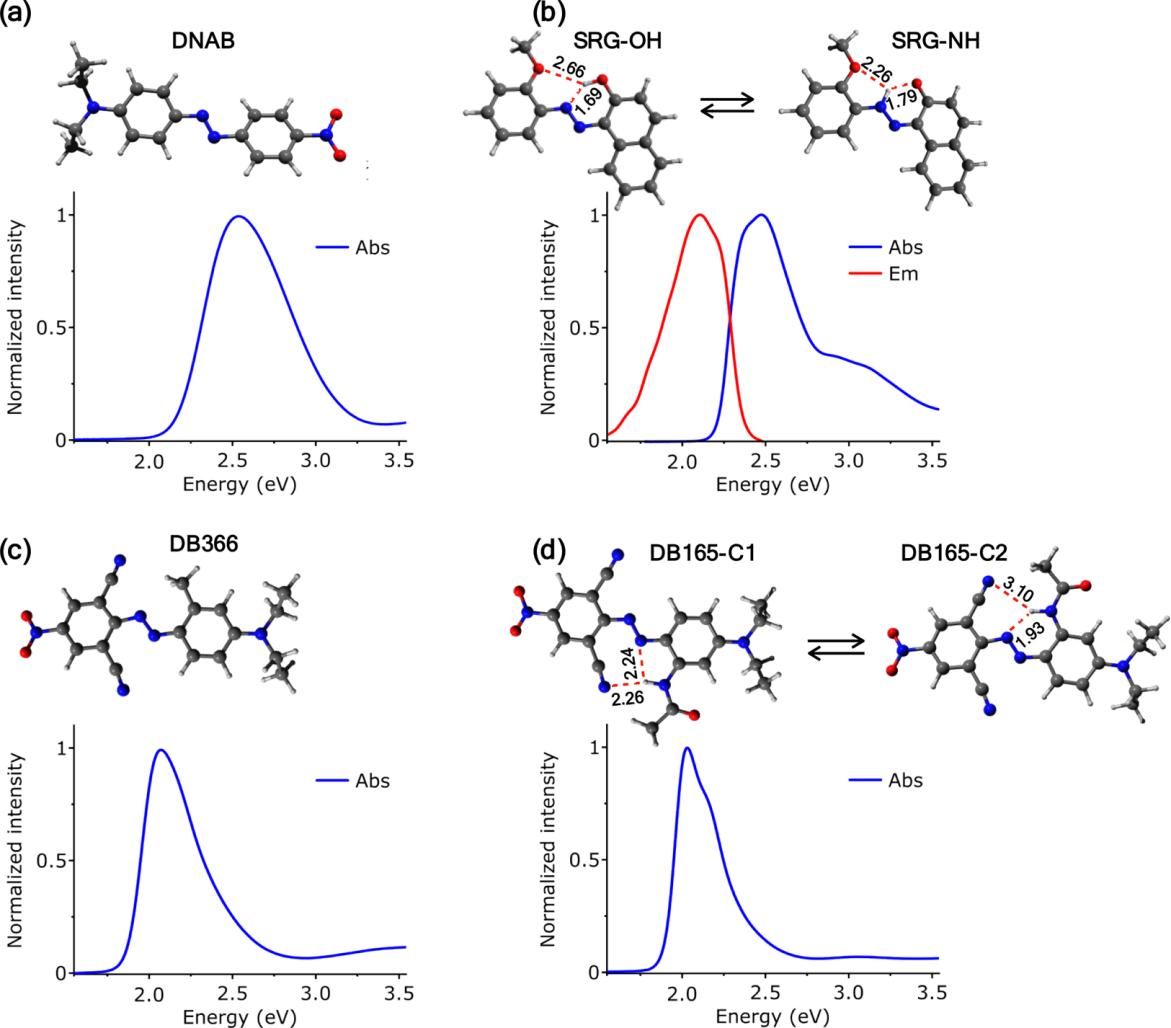
Linear absorption spectra and M06/6-31G* optimized structures of (a) DNAB, (b) SRG, (c) DB366, and (d) DB165. The red dashed lines label intramolecular hydrogen bonds (distances in angstroms). For SRG, the emission spectrum is also reported.

Figure 1 shows the linear absorption spectra of the four studied chromophores in methanol. In the linear absorption spectrum of DNAB (Figure 1a), a broad and featureless band is observed, peaking at 2.54 eV. As mentioned earlier, the introduction of donor–acceptor substitutions in push–pull azobenzene derivatives significantly enhances the charge transfer (CT) character of the ππ* electronic transition, causing a redshift in the bright ππ* transition.^37^ The class of Disperse Blue dyes with additional electron donor and acceptor substitutions exhibits increased CT character, leading to a considerable redshift in their absorption maxima. For instance, DB366 displays a broad absorption peak at 2.07 eV (Figure 1c), while DB165 shows a structured absorption peaking at 2.03 eV and a shoulder around 2.18 eV (Figure 1d). Fluorescence was not detected in the three abovementioned compounds, possibly due to the well-known conical intersection (CI) pathways, transitioning the bright ππ* state to the dark *n*π* state on an ultrafast time scale. In contrast, SRG exhibits a well-structured steady-state absorption spectrum with a main peak at 2.47 eV and a shoulder at 2.95 eV (Figure 1b). Interestingly, the emission spectrum peaks at 2.10 eV and has a mirror-image structure with respect to the main absorption peak of SRG, resulting in a Stokes shift of 2984 cm^–1^. This indicates that the 2.95 eV shoulder is not a vibrational replica of the 2.47 eV peak from which it is blueshifted by ≈3900 cm^–1^, but it rather represents a different electronic transition (see below). Furthermore, the 2.47 eV absorption and 2.10 eV emission peaks arise from the bright ππ* state, and its possibly longer lifetime allows for detectable emission compared with the other compounds. The emission quantum yield was calculated to be 0.1%, using rhodamine B in water as a reference sample (see the Supporting Information).

For the computational characterization of DNAB and DB366, only one structure was considered (Figure 1a,c). On the other hand, two different hydrogen bond patterns were identified for SRG and DB165. The former is subject to keto–enol tautomerism, yielding the two structures SRG-OH and SRG-NH (Figure 1b). In the case of DB165, the rotation of the phenyl ring bearing the −NHCOCH_3_ substituent yields the two conformers DB165-C1 and DB165-C2 (Figure 1d) showing a different pattern of hydrogen bonds. For each structure, we have characterized QM/MM excited state minima to identify possible bright transitions that match the observed experimental signals. In addition, we have run a QM/MM nuclear trajectory starting from the bright state at the FC point without initial velocity (0K dynamics, details in the Supporting Information) to investigate the most relevant initial reactive coordinates and locate the most accessible CIs. The presence and accessibility of S_1_/S_0_ CIs were also studied by means of PES exploration in implicit solvent (PCM). Because the chromophores show a different time evolution of the transient signals, their spectral interpretation and proposed deactivation mechanism will be discussed separately in the following.

### 4.1 4-diethyl-4′-nitroazobenzene (DNAB)

#### 4.1.1 Origin of the Transient Signals

**Figure 2:**
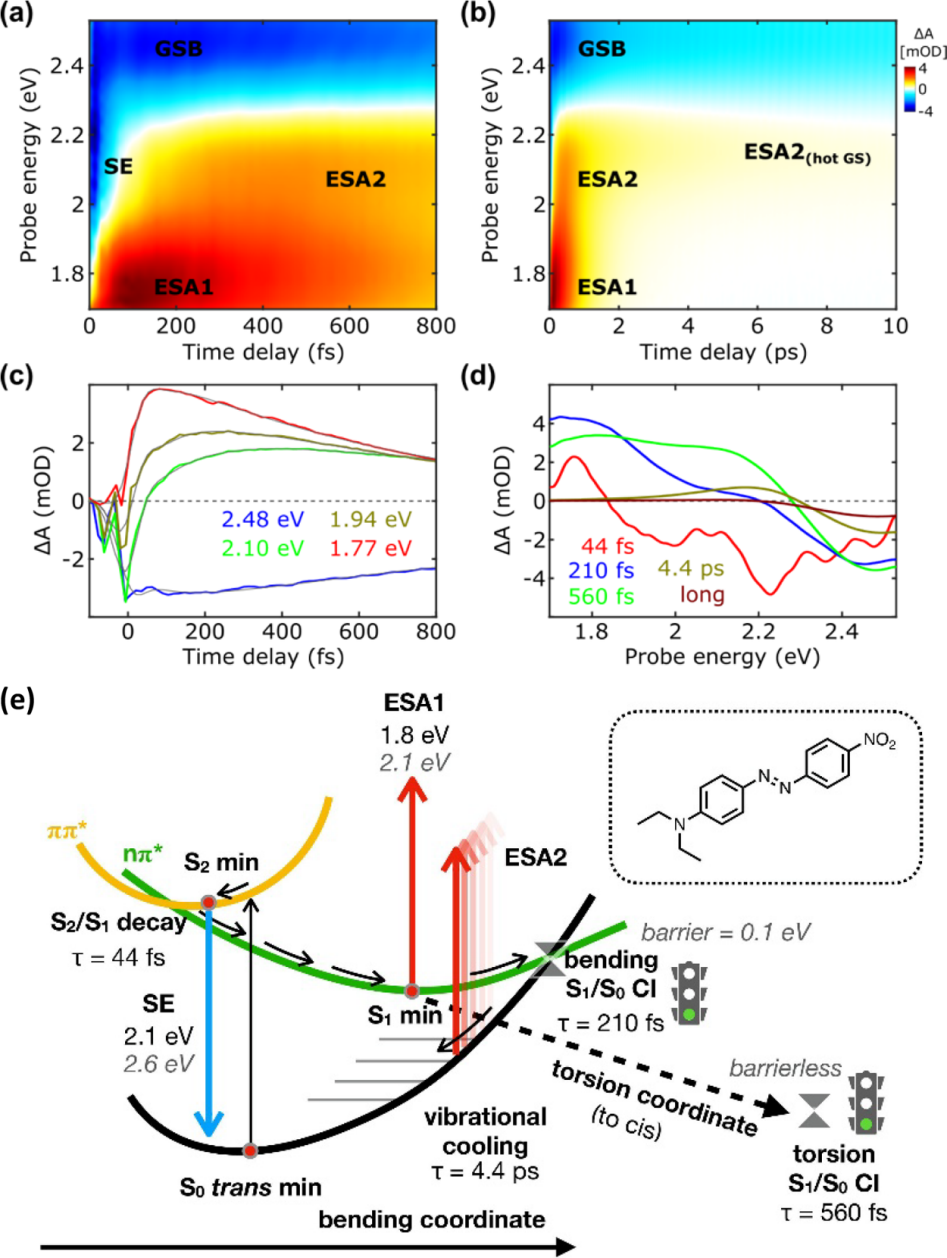
TA maps in the (a) first 800 fs and (b) until 10 ps of DNAB. (c) Selected kinetic traces, focusing on the sub-800 fs time scale, for selected probe wavelengths superimposed by the fitting obtained by global analysis. (d) The EAS and their corresponding time constants were retrieved by global analysis. (e) A schematic representation of the deactivation mechanism is proposed based on the experimental observations (values in black) and computational results (gray, italics).

The time evolution of the TA signals is depicted in the maps and kinetics presented in Figure 2a–c. Figure 2d shows the evolution associated spectra (EAS) and corresponding time constants obtained through multiexponential global fitting of the experimental data sets (see methods section for details). The results of the computational characterization of DNAB are collected in Table 1. We note that the linear absorption spectrum of this compound shows a broad band peaking at 2.54 eV (Figure 1a) that corresponds to the S_0_ → S_2_ (ππ*) excitation (2.61 and 2.67 eV for TDDFT and RASPT2, respectively; see Table 1), overlapping partially with the pump spectrum (1.85–2.43 eV). At 50 fs pump–probe delay, right after photoexcitation, the observed signatures of the ground state bleaching (GSB) and stimulated emission (SE) signals in the TA map are associated with the population of the bright state (S_2_). The S_2_ minimum geometry is very similar to the FC geometry (see CNNC and CNN values in Table 1) and corresponds to a quasi-degeneracy point between S_2_ (ππ*) and S_1_ (*n*π*, dark). Indeed, the bright state lies close to S_1_ already at the FC point (Δ*E*_S2–S1_ = 0.30 and 0.14 eV at the TDDFT and RASPT2 level, respectively). Thus, the system is expected to oscillate on S_2_ for a very short time before an ultrafast IC to the lower-lying dark state takes place, as confirmed by 0K dynamics (see the Supporting Information). This scenario is perfectly in line with the ultrafast disappearance of the SE (2.1 eV, τ = 44 fs) signal associated with S_2_. The TDDFT S_2_–S_0_ gap at the S_2_ minimum (2.24 eV) is in good agreement with the experimental SE energy, while RASPT2 is overestimating the SE position (2.62 eV), probably because the excited state was optimized at the TDDFT level.

**Table 1:**
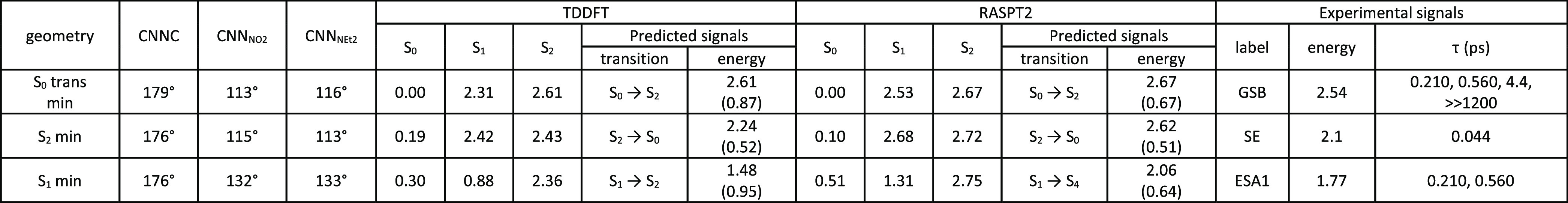
Relevant Geometrical Parameters and Electronic State Energies (in eV) at the QM/MM S_0_, S_2_, and S_1_ Minima of DNAB^a^

a The last two rows report the calculated bright transitions (energy in eV, oscillator strength in parentheses) and the peak position (eV) of the corresponding experimental signals (the associated time constants are reported in parentheses).

The fast decay of SE is associated with the rise of an excited state absorption (ESA) signal at 1.77 eV (labeled ESA1), whose amplitude peaks at 100 fs and decays with two time constants of 210 and 560 fs (Figure 2c,d). Following the above considerations about the S_2_/S_1_ vicinity, ESA1 is thus likely to be a signature of S_1_. The S_1_ minimum is a planar structure with wider CNN angles compared to the FC geometry (113°/116° vs 132°/133° for CNN_NO2_/CNN_NEt2_ at the S_0_ vs S_1_ minima, respectively, see Table 1). This planar and symmetric S_1_ minimum was already reported for the parent azobenzene molecule^26,27^ and endorses the similarity between the PES of the two compounds. From the planar S_1_ minimum, a bright transition toward another *n*π* state was identified both at TDDFT and RASPT2 level (Table 1), although the two predictions are almost equally shifted by ∼0.3 eV (to the red for TDDFT and to the blue for RASPT2) from the experimental ESA1 peak (see Table 1). However, the RASPT2 arrival state (S_4_) shows a significant contribution from a doubly excited configuration, and it is therefore probable that TDDFT lacks accuracy in its description. Overall (considering also that the geometry was optimized at the TDDFT level), RASPT2 shows a notable accuracy and validates the association of the ESA1 signal with the population of the S_1_ state.

The biexponential decay of ESA1 indicates the presence of two distinct depopulation processes for S_1_. Because DNAB is a typical push–pull azobenzene derivative (with no other substituents that might bring any new unexpected behavior), it is very likely that the two time constants are associated with the bending (210 fs) and torsion (560 fs) pathways already known for azobenzene. Starting from 100 fs onward, the decay of ESA1 coincides with the emergence of ESA2, as evidenced by the dynamics observed at 2.1 eV in Figure 2c and the decays of blue and green EAS spectral components in Figure 2d. The ESA2 signal reaches its peak intensity at 400 fs and initially experiences a blueshift of its maximum (see Figure S16). Around 3 ps, the blueshifted ESA2 signal becomes localized at approximately 2.17 eV, and it is characterized by a prolonged lifetime (τ = 4.4 ps). The spectral shape and the associated time scales strongly suggest that ESA2 may originate from ground state vibrational cooling following S_1_/S_0_ decay (i.e., from a ππ* transition far from the equilibrium resulting in a broadening of the red-edge ground-state absorption). Indeed, the position and lifetime of ESA2 are in line with the “hot” ground state recovery reported for other azo compounds.^39−43^ Interestingly, at later time scales, ESA2 undergoes a redshift until 20 ps, resulting in a positive signal around 2.08 eV with small amplitude. In similar azo compounds, the redshifted residual ESA signal has been attributed to the cis isomer.^41,42^ However, our calculations exclude the possibility that the redshifted ESA2 comes from the cis-DNAB photoproduct (which absorbs outside the probe window; see Table S1). Furthermore, ESA2 completely vanishes within 40 ps, contrasting the scenario of ESA2 stemming from long-lived species. Consequently, we favor the hypothesis that ESA2 exclusively originates from the hot ground state, with the redshift resulting from the competing filling of the red-edge GSB transitions. After ESA2 extinction, at 30–40 ps, 84% of the initially observed GSB signal has recovered, where it reaches a plateau level, remaining persistent up to 1.2 ns (Figures S15 and S16). This long-living signal is attributed to the cis isomer, suggesting that photoisomerization is taking place with significant yields (see the Supporting Information for details). The TDDFT and RASPT2 excited state calculations at the cis equilibrium geometry agree on the absence of bright transitions in the detection window from this isomer (see Table S1), thus indirectly endorsing this hypothesis. It must be noted, however, that our quantum yields should not be considered as absolute values and should not be directly compared as such. Instead, they should be interpreted as relative quantum yields that allow to compare the photoisomerization productivity within our set of chromophores, as explicitly mentioned in the Supporting Information.

#### 4.1.2 Presence and Accessibility of Conical Intersections

The exploration of the S_1_ PES along the CNNC torsion and CNN_NO2_/CNN_NEt2_ bending coordinates was conducted with implicit solvation as described in the computational details, and the electronic energies at the relevant points are reported in Table 2. Both the torsional and symmetric bending deactivation routes typical of the azo group were confirmed for DNAB. Both these crossings are accessible after S_2_ excitation (i.e., lying below the excitation energy). However, in contrast to the parent azobenzene molecule, S_2_ and S_1_ are already very close at the trans S_0_ minimum, and a S_2_/S_1_ crossing is in the immediate vicinity of the FC point (as discussed before). Therefore, even though the “bending CI” is energetically accessible after photoexcitation to S_2_ (as demonstrated also by 0K dynamics, see the Supporting Information), the CNN_NO2_/CNN_NEt2_ bending oscillations are expected to be damped in DNAB compared to azobenzene. At the same time, the torsional S_1_/S_0_ CI is found to be at lower energy than the bending one (see Table 2), being also the absolute minimum on S_1_. These findings suggest that the energy transfer to the torsional motion is enhanced in DNAB, exactly as expected for a typical push–pull azobenzene.^23^

**Table 2:**
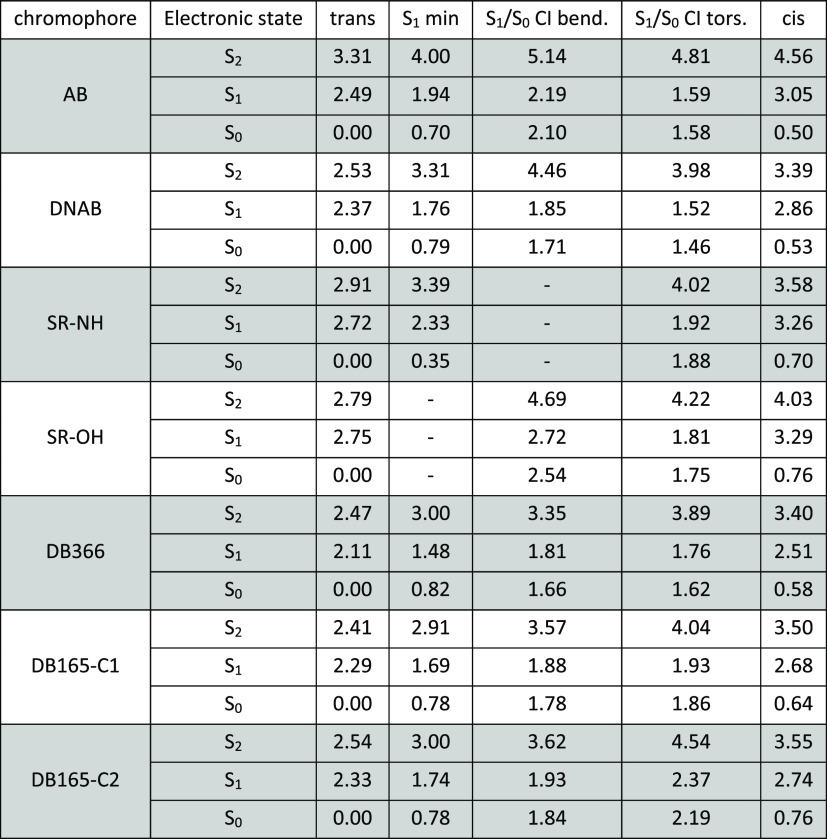
Relevant Points on the PCM (methanol) S_1_ Potential Energy Surfaces of Azobenzene (AB) and of the Investigated Chromophores^a^

a All energies are reported in eV.

#### 4.1.3 Deactivation Model

Figure 2e shows a scheme for the deactivation model proposed for DNAB. After S_2_ excitation, the system undergoes ultrafast IC to the dark S_1_ state (τ = 44 fs), causing the decay of the SE signal. Subsequently, the system relaxes on S_1_ along the symmetric bending coordinate to reach the S_1_ planar minimum from which a bright transition to a higher lying state originates ESA1. Over time, part of the S_1_ population remains in the planar region, and the symmetric bending oscillations lead to unproductive S_1_/S_0_ crossing (τ = 210 fs). This represents the faster deactivation channel, which triggers the rise of ESA2 as a fingerprint of vibrational cooling on the ground state. Another significant part of the population survives longer on S_1_, allowing internal vibrational energy redistribution to the slower (productive) torsional coordinate (τ = 560 fs) and producing the cis photoproduct, as testified by the GSB signal surviving until the end of the our temporal pump-probe window (1.2 ns).

### 4.2 Sudan Red G

#### 4.2.1 Origin of the Transient Signals

The linear absorption spectrum of SRG (Figure 1b) shows a main peak at 2.47 eV with a shoulder around 2.95 eV. Looking at the vertical excitation energies of the two tautomers (2.63 vs 3.31 at the RASPT2 level, see excitation energies at the S_0_ minima in Tables 3 and S3), the main absorption peak can be associated with the bright S_0_ → S_1_ (ππ*) transition of SRG-NH (RASPT2 being more accurate than TDDFT). In contrast, the bright ππ* transition for SRG-OH is toward S_2_ and its higher energy matches very well the shoulder in the absorption spectrum. This hypothesis is further strengthened by the shape of the emission spectrum (Figure 1b), which shows the mirror replica of the main peak (emissive S_1_ of SRG-NH) but without any shoulder (S_1_ of SRG-OH is dark). In addition, the ratio between the RASPT2 oscillator strengths (NH/OH = 0.43/0.14) is in perfect agreement with the peak/shoulder intensity ratio of the experimental spectrum for an almost 1:1 mixture of tautomers. Only the main peak falls within the spectral range of the pump pulse (1.85–2.43 eV), while the shoulder is outside the excitation window; therefore, we assume that only the SRG-NH tautomer is excited in the TA experiments. Moreover, no bright transitions matching the experimental signals were found from the S_2_ or S_1_ minima of the SRG-OH tautomer. For this reason, only SRG-NH QM/MM results will be discussed in the following (details on the characterization and 0K dynamics of SRG-OH are found in the Supporting Information).

**Table 3:**
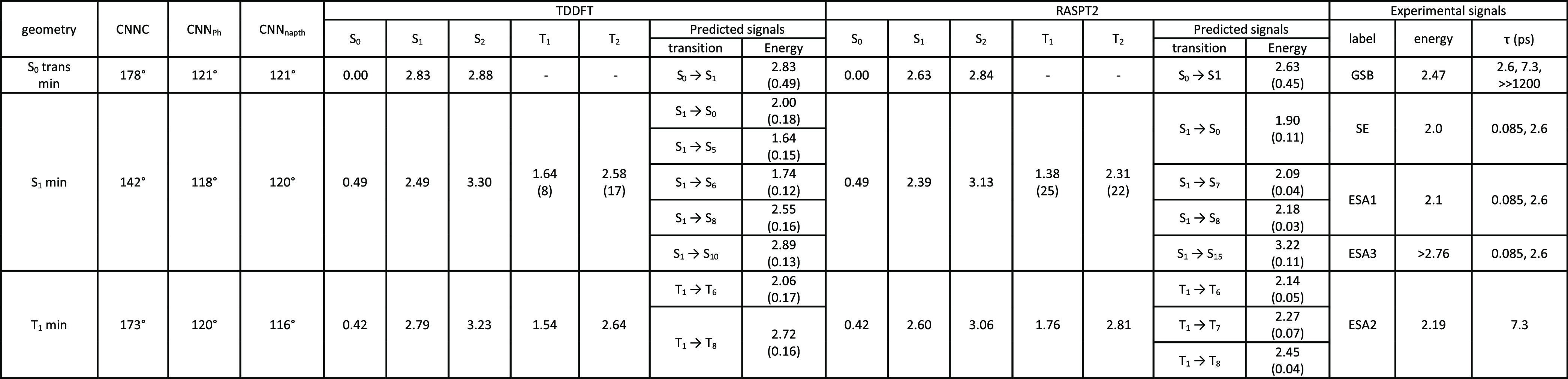
Relevant Geometrical Parameters and Electronic State Energies (in eV) at the QM/MM S_0_, S_1_, and T_1_ Minima of SRG-NH^a^

a For the triplet states, the SOC magnitude from S_1_ is also reported in parentheses. The last two rows report the calculated bright transitions (energy in eV, oscillator strength in parentheses) and the corresponding peak position (eV) of the corresponding experimental signals (the associated time constants are reported in parentheses). For S_1_ and T_1_ minima, the QM/MM energy gaps were added to the S_0_ energy in PCM (relative to S_0_ trans min in PCM).

**Figure 3:**
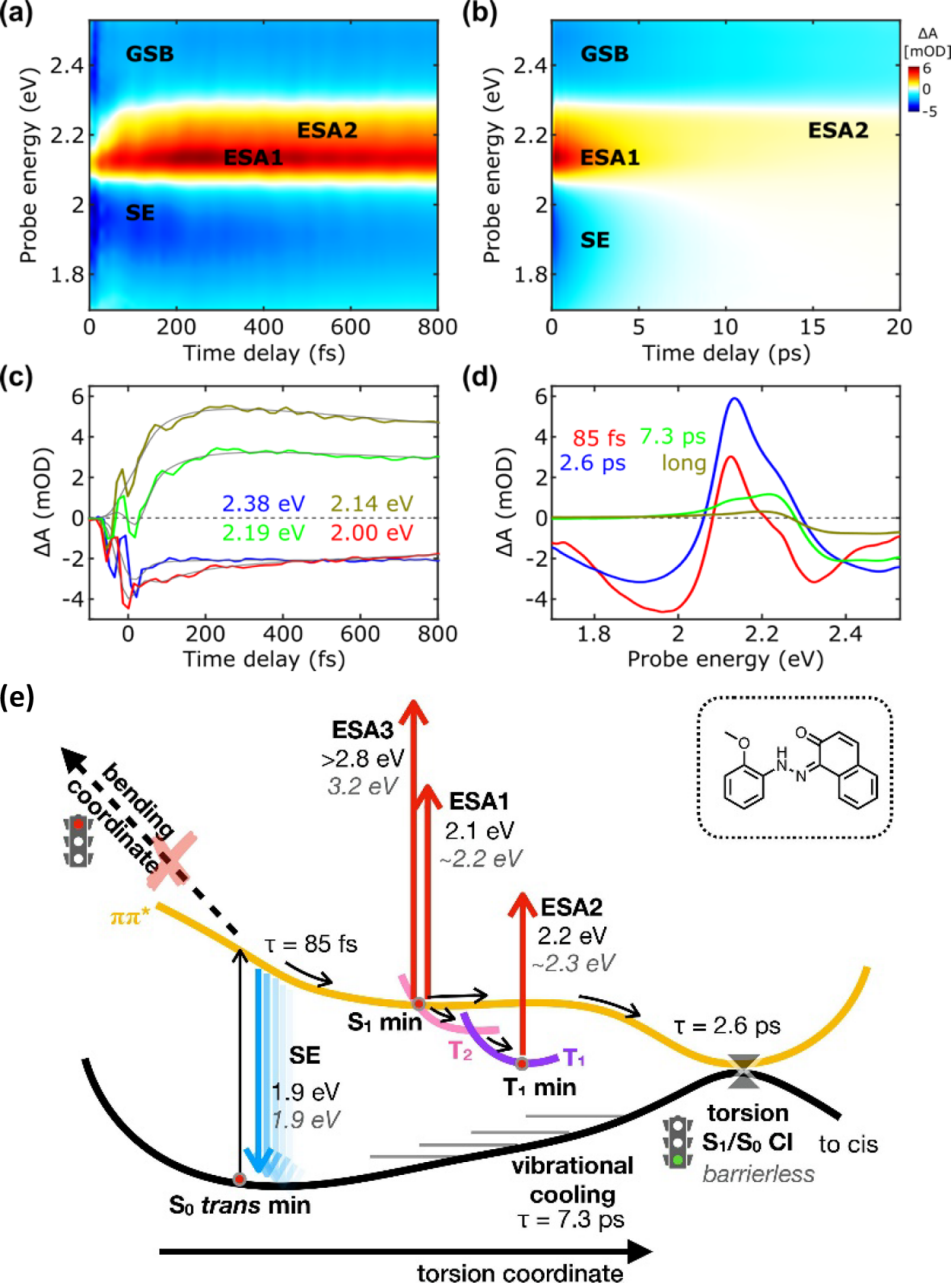
TA maps in the (a) first 800 fs and (b) until 20 ps of SRG. (c) Selected kinetic traces, focusing at the sub-800 fs dynamics, for selected probe wavelengths superimposed by the fitting obtained by global analysis. (d) The EAS and their corresponding time constants as retrieved by global analysis. (e) A schematic representation of the deactivation mechanism is proposed for SRG-NH tautomer based on the experimental observations (values in black) and computational results (gray, italics).

Figure 3a–d shows the time evolution of the TA signals and the corresponding lifetimes extracted by global analysis. As shown in Figure 3c, oscillatory signals are superimposed on the slowly varying population dynamics. These features, also observed in other samples, represent coherent molecular vibrations, impulsively excited by the ultrashort pump pulse and not further discussed here. The population of S_1_ in SRG-NH by the pump pulse is manifested by the immediate presence of GSB (2.47 eV), SE (2.0 eV), and ESA1 (2.14 eV) spectral signatures. We observe that the SE signal, which initially peaks at 2.0 eV and experiences a redshift with a time constant of 85 fs, exhibits a significant shift when compared to the steady-state fluorescence at 2.1 eV. At later time intervals, it reaches a peak at 1.91 eV, as evident in the blue EAS spectrum presented in Figure 3d. This substantial mismatch between SE and fluorescence signals is likely due to the spectral overlap of the competing SE and ESA1 signals in the 2.15 eV energy region. Indeed, as SE undergoes the redshift with an 85 fs time constant, ESA1 concurrently rises, reaching its maximum amplitude, and experiences a slight blue-shift (refer to the dynamics in Figure 3d and the shifted signatures between the first two reported EAS components in Figure 3d). The spectral responses of SE and ESA1 are consistent with a movement across the ππ* PES toward an excited state minimum. Another transition associated with the ππ* state, exhibiting a similar spectral response, is the experimentally resolved ESA3 located at 2.76 eV. Notably, ESA3 is visible primarily in the difference spectra (as shown in Figure S18), where a wider probe energy window is displayed. In addition, SE and ESA1 (see Figure S18 for ESA3) decay simultaneously with a time constant of 2.6 ps, highlighting that all of these transitions are associated with the same state (S_1_, ππ*). At longer times, another ESA signal becomes pronounced (ESA2, 2.19 eV), which survives up to 1.2 ns (see Figures S17 and S18), together with GSB.

To assess the origin of the observed signals, S_1_ was optimized for SRG-NH (QM/MM) yielding a partially rotated geometry in which the central CNNC dihedral angle has reached 142° (Table 3). The presence of this out-of-plane minimum was also confirmed by subsequent PCM calculations (see below), suggesting that this is a real feature of the S_1_ PES, and not a QM/MM artifact due to droplet conformations. Both TDDFT and RASPT2 suggest that S_1_ is still bright in its minimum (ππ*), with a predicted SE energy (S_1_ → S_0_) in very good agreement with the experiment (2.00 and 1.90 eV at TDDFT and RASPT2 level, respectively, see Table 3). However, the relaxation along CNNC torsion causes the mixing between ππ* and nπ* state character, lowering the oscillator strength. The decrease in the S_1_ → S_0_ brightness with respect to the FC point agrees with the continuous decay of the SE signal after photoexcitation (see Figure 3c). Concerning the positive signals, TDDFT fails to accurately reproduce ESA1 and ESA3: four bright transitions from S_1_ were identified (S_1_ → S_5_, S_1_ → S_6_ of ππ* nature and S_1_ → S_8_, S_1_ → S_10_ of nπ* nature, see Table 3), but most of them fall in a region that does not show any experimental ESA signal. In contrast, RASPT2 is remarkably accurate not only for the SE prediction but also for the two ESAs: two close-lying weak but bright transitions are found around 2.1 eV (S_1_ → S_7_ of nπ* nature and S_1_ → S_8_ of mixed nπ*/ππ* nature), which could add up to produce ESA1, and at the same time the tail of the S_1_ → S_15_ band at higher energy could originate ESA3 (which is only detected as a tail in the TA spectrum, see Figure S18). The higher excited states appear to be much multiconfigurational in nature, with contributions from doubly excited configurations, thus endorsing the higher accuracy of RASPT2 predictions. The GSB recovers with an additional lifetime of 7.3 ps, due to the vibrational cooling of the hot ground state.

At later time scales, 79% of the initially observed GSB has decayed and a long component (≫1.2 ns) is needed to properly fit the remaining long-lived signal. This indicates that part of the population is not returning to the initially excited trans isomer. This might be due to photoisomerization with formation of the cis isomer. However, also in this case, the predicted cis excitation energy (both at TDDFT and at RASPT2 level, see Table S1) falls outside the detection window; thus, we have no possible signals to confirm this hypothesis.

Looking at the EAS difference spectra of Figure 3d extracted by global analysis, the ESA2 signal is also surviving until very long times (≫1.2 ns, see also the additional spectra in Figures S17 and S18). This suggests that there might be another long-living species in which part of the population gets trapped. The long ESA2 lifetime suggests that this species might be in a triplet state. In order to assess the probability of intersystem crossing (ISC) from the S_1_ minimum, we have evaluated the lowest triplet energies of SRG-NH at this geometry, as well as the SOC magnitude for each S_1_ → T_n_ transition, which are reported in Table 3. The T_2_ state turns out to be very close in energy to S_1_ (Δ*E* = 0.09 and 0.08 eV at TDDFT and RASPT2 levels, respectively) and with a small but non-negligible SOC value. To assess the possibility of a triplet origin of ESA2, both T_2_ and T_1_ were optimized. Indeed, after a possible ISC to T_2_, the electronic population is expected to be soon transferred to the lowest triplet state T_1_, and any long-living signal is likely to come from this state. Indeed, no matching bright transitions were identified from the T_2_ minimum, while a group of three close-lying bright transitions around 2.2 eV were identified from the T_1_ minimum (RASPT2, see Table 3), from which ESA2 could originate.

Overall, RASPT2 calculations suggest that the short-living SE, ESA1, and ESA3 signals are associated with the initially populated S_1_ state, while the long-living ESA2 is probably associated with the population of a triplet state (T_2_ and then T_1_). TDDFT on the other hand is very accurate for what concerns SE, while its accuracy is much lower in predicting ESAs. This is a known pitfall of this method, which results from its deteriorated accuracy in predicting high-energy electronic states and the transition dipole moment between them.^44^

#### 4.2.2 Presence and Accessibility of Conical Intersections

The results of the PCM study for both SRG-NH and SRG-OH are reported in Table 2. The main difference between the two tautomers is the fact that SRG-OH shows both the bending and torsional CIs typical of the azo dyes (both accessible after S_2_ excitation, see Table 2) while the PES of SRG-NH is not showing any crossing along the symmetric CNN/NNC bending. Indeed, the S_1_ state in this case has a different character (ππ*), and the proton shift to the N atom makes this structure an “improper” azobenzene derivative (characterized by a single rather than double NN bond); therefore, it is not surprising that it shows some differences with respect to the typical behavior of the azo group. Instead, the torsional CI is preserved and is accessible from the FC point (Table 2). The absence of the bending deactivation channel might further enhance the quantum yield of this molecule. The S_1_ minimum reported in Table 2 is a partially rotated structure very similar to the QM/MM minimum discussed before, further suggesting that the torsional motion is favored on S_1_ for SRG-NH. The enhanced accessibility of the torsional decay pathway is also confirmed by 0K dynamics (see the Supporting Information), endorsing the hypothesis of photoisomerization with formation of the cis isomer (GSB survival). On the other hand, the impossibility to include ISC and triplet states in the simulation did not allow the confirmation of the triplet hypothesis (long-living ESA2).

#### 4.2.3 Deactivation Model

Figure 3e is a scheme for the deactivation model of SRG-NH. After photoexcitation, the system immediately relaxes in the bright S_1_ state (τ = 85 fs). The absence of lower lying excited singlets allows the SE, ESA1, and ESA3 signals to survive longer compared to the other azo dyes (τ = 2.6 ps). However, in the vicinity of the S_1_ minimum, the system might undergo ISC toward T_2_ and then T_1_ from which a bright transition toward higher triplets originates ESA2 (τ = 7.3 ps). The part of the population that does not undergo ISC is prone to continue along the torsional coordinate to reach the S_1_/S_0_ CI (corresponding to the absolute minimum on S_1_). As the latter is a potentially productive deactivation path, some cis photoproduct is formed (as proved by GSB survival). The relatively lower percentage of GSB recovery for SRG (79%) in comparison to DNAB (84%), within the margin of error in our approach, can be attributed to both the absence of the unproductive pathway along the bending coordinate and the activation of the channel of triplet states in the case of SRG-NH.

### 4.3 Disperse Blue 366

#### 4.3.1 Origin of the Transient Signals

**Figure 4:**
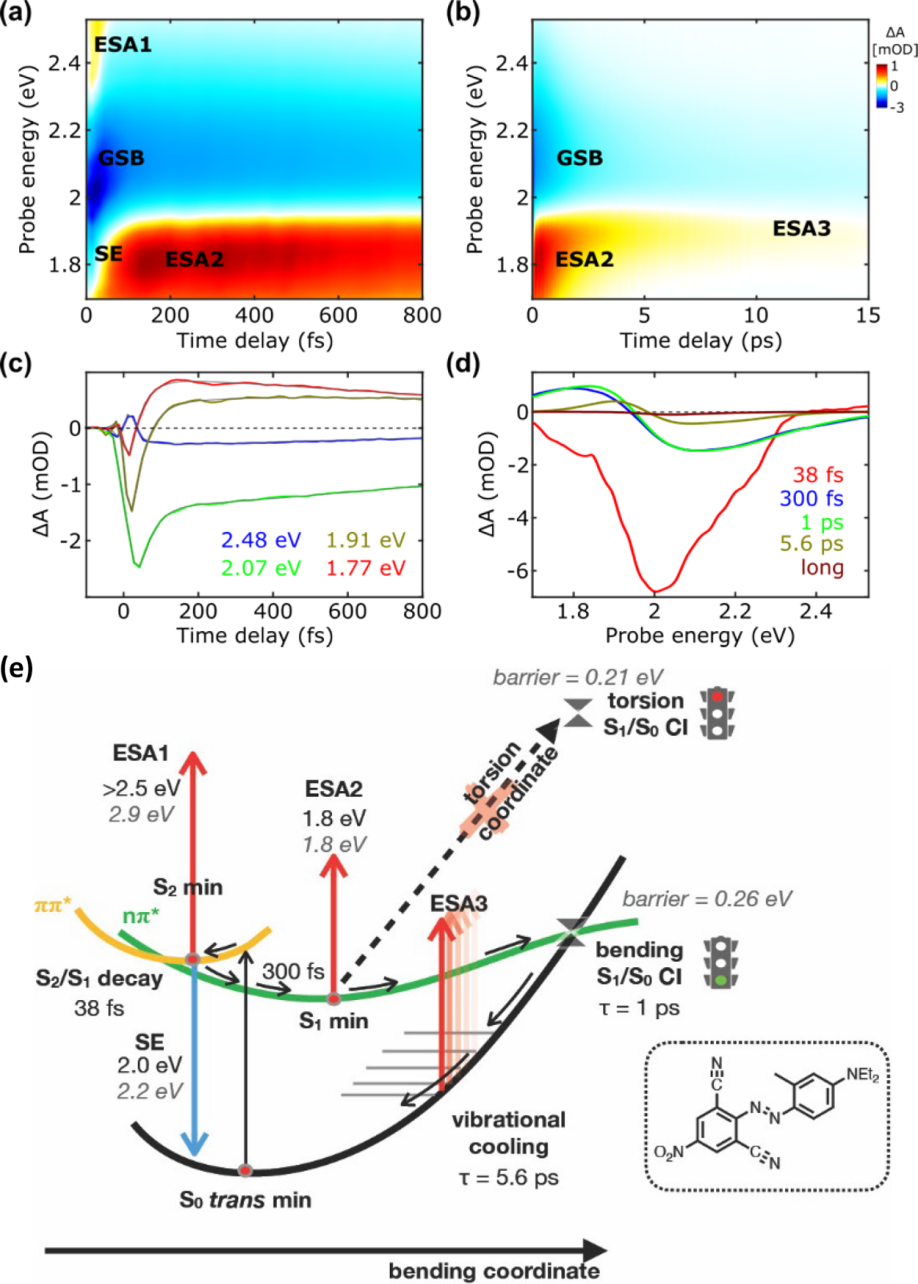
TA maps in the (a) first 800 fs and (b) until 15 ps of DB3GG. (c) Selected kinetic traces, focusing on the sub-800 fs dynamics, for selected probe wavelengths superimposed by the fitting obtained by global analysis. (d) The EAS and their corresponding time constants as retrieved by global analysis. (e) A schematic representation of the deactivation mechanism is proposed based on the experimental observations (values in black) and computational results (gray, italics).

The linear absorption spectrum of DB366 (Figure 1c) consists of a broad and unstructured band peaking at 2.07 eV, showing an excellent overlap with the pump pulse spectrum. Both TDDFT and RASPT2 predict that the populated bright state corresponds to S_2_, although both methods overestimate the excitation energy. The TDDFT error is particularly large (0.5 eV, see Table 4), while the RASPT2 error is in line with that of the computational method (0.2 eV), considering also that the geometry optimization was performed at the DFT level. The TA spectrum (Figure 4a,b) shows a negative (SE, 1.85 eV) and a positive (ESA1, 2.45 eV) signal rising immediately after photoexcitation that disappear at the limits of our temporal resolution. These two signals are associated with the initially populated S_2_, and their lifetime (38 fs as viewed in Figures 4c,d) suggests an ultrafast depopulation of this state. Indeed, the bright state is already very close to S_1_ both at the FC point (according to RASPT2, see Table 4) and at the nearby S_2_ minimum, suggesting a high probability of S_2_ → S_1_ transfer immediately after photoexcitation.

**Table 4:**
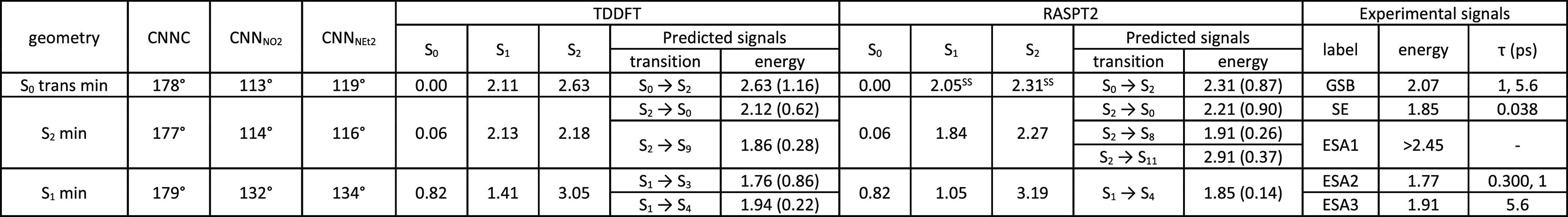
Relevant Geometrical Parameters and Electronic State Energies (in eV) at the QM/MM S_0_, S_2_, and S_1_ Minima of DB366^a^

a The last two rows report the calculated bright transitions (energy in eV, oscillator strength in parentheses) and the corresponding peak position (eV) of the corresponding experimental signals (the associated time constants are reported in parentheses). For S_2_ and S_1_ minima, the QM/MM energy gaps were added to the S_0_ energy in PCM (relative to S_0_ trans min in PCM). ^SS^ = SS-RASPT2 energy.

At the S_2_ minimum, the calculated SE energy matches the detected signal (for both TDDFT and RASPT2). The assignment of ESA1, instead, requires more care. Both TDDFT and RASPT2 find a bright transition toward another ππ* state around 1.9 eV (see Table 4), which is not visible in the experimental spectrum. However, the oscillator strength associated with this ESA signal is much weaker compared to that of SE and GSB (see Table 4) and it is therefore likely that this signal gets buried under the stronger negative GSB. No other bright transitions from S_2_ are found by TDDFT, while RASPT2 predicts a second ESA at higher energy toward a doubly excited ππ* state (S_2_ → S_11_ = 2.91 eV, see Table 4) that could not be detected by TDDFT and that peaks just outside of our experimental detection window. The tail of this transition might correspond to ESA1 in the experimental TA spectrum (which is indeed visible as a tail; see Figure 4a).

After 60 fs, SE and ESA1 disappear, while the ESA2 signal rises at 1.77 eV (Figure 4c,d). This signal is therefore likely to be originated by S_1_. The decay of the ESA2 signal is experimentally associated with two distinct time constants: 300 fs and 1 ps, indicating the presence of two different pathways on the S_1_ surface. Over time, ESA2 exhibits a slight blueshift toward 1.82 eV with a 300 fs time constant, as seen in the transition from the blue to green EAS in Figure 4d. Interestingly, this shift occurs without an accompanying GSB signal recovery, suggesting a movement across the PES without the presence of population decay. On the other hand, ESA2 experiences decay with a time constant of 1 ps, leading to the transition from green to gold-colored EAS in Figure 4d. This decay is attributed to the nonradiative relaxation back to the ground state, as manifested by the GSB recovery.

The time evolution of EAS2 eventually results in the formation of a localized peak at 1.91 eV, denoted as ESA3, displaying spectral characteristics previously associated with the hot ground state. Notably, the ESA3 (1.91 eV) peak vanishes with a time constant of 5.6 ps along with the majority of the GSB signal (approximately 98% of the initial GSB). This indicates that the population has nearly fully returned to the starting trans S_0_ minimum without undergoing photoisomerization (for additional details, refer to the Supporting Information section). Overall, the findings suggest a fast relaxation on S_1_ (τ = 300 fs), followed by S_1_/S_0_ decay (τ = 1 ps) and hot ground state cooling (τ = 5.6 ps). Indeed, the energy and long lifetime of the 650 nm peak are in line with the “hot” ground state recovery reported for other azo derivatives.^39−43^

Similarly to the previously discussed DNAB (and other azobenzene derivatives), the S_1_ optimization from the trans FC point yielded a planar structure, reached by opening the CNN bending angles (see Table 4). From here, we found a group of close-lying bright transitions toward higher *n*π* states (Table 4) whose energy matches very well with that of ESA2 and whose rise might be associated with the relaxation on S_1_. At later times, S_1_/S_0_ decay takes place and the S_1_ ESA2 might start to overlap with (and in time to be replaced by) the “hot” ground state recovery (ESA3). The concurrent ESA3 and GSB disappearance suggests that the S_1_/S_0_ decay must take place through a nonreactive channel, because the full population returns to the initial trans isomer within 10 ps. This could mean that only the “bending pathway” (i.e., deactivation through the planar S_1_/S_0_ CI already documented for azobenzene^25−28^) is accessed in DB366, although a full set of dynamics simulations is required to confirm this hypothesis.

#### 4.3.2 Presence and Accessibility of Conical Intersections

Like in the case of DNAB and any typical azobenzene derivative, both bending and torsional CIs were located (Table 2). However, in contrast to the parent compound, the two CIs are almost isoenergetic and they both lie higher in energy with respect to the S_1_ minimum. Even if they are isoenergetic and in principle both accessible after S_2_ excitation, the bending CI is expected to be kinetically favored, because bending oscillations are immediately activated after photoexcitation (see also 0K dynamics in the Supporting Information), while the torsional motion requires time for internal vibrational energy to be redistributed.

To further investigate the accessibility of the two CIs, a relaxed scan in PCM was conducted along CNNC torsion and CNN bending (Figures S23 and S24) to explore the PES topology along the path connecting the FC point to the crossing points. The bending CI lies 0.26 eV above the S_1_ planar minimum, which is less than half the gap observed in azobenzene (i.e., 0.6 eV^25^). On the other hand, the energy gap between the S_1_ minimum and the torsional CI was found to be 0.21 eV, with a small barrier of 0.11 eV along the path connecting them (at CNNC = 140°) that enhances the negative torsional slope in the vicinity of S_1_ minimum. This might be a consequence of the interaction between one of the methyl hydrogens and the nitrogen lone pair (methyl H bond^45,46^), which is broken along the torsional coordinate, originating a barrier. Compared with azobenzene, whose “torsional” CI is located 0.10 eV below the S_1_ minimum,^25^ it is reasonable to think that the torsional CI in DB366 will not be easily accessible.

#### 4.3.3 Deactivation Model

Figure 4e shows the proposed deactivation model for DB366. The population of the bright S_2_ state produces SE and ESA1 signals, which decay in less than 60 fs as a consequence of the ultrafast IC to the close-lying S_1_. After this, the population relaxes on S_1_ (τ = 300 fs) along symmetric bending, causing the rise of ESA2. On S_1_, the wavepacket is probably not prone to transfer part of the vibrational energy to the torsional motion, as the presence of a methyl H-bond stabilizes the planar structure and creates a barrier along CNNC torsion. In contrast, the symmetric bending oscillations lead primarily to the planar (unproductive) region of the S_1_/S_0_ crossing seam (τ = 1 ps), preventing isomerization and causing GSB disappearance in some ps. The longer bending lifetime compared to the less substituted DNAB (τ = 210 fs, see before) can be addressed to a combination of both the higher barrier connecting the S_1_ minimum and the bending CI (0.1 eV in DNAB vs 0.2 eV in DB366) and the increased inertia brought by the additional substituents of DB366. Moreover, it has been demonstrated by previous studies that the space-demanding motion of large molecular fragments in condensed phase is subject to a larger “dynamical” energy barrier, originated by the need to displace a large number of solvent molecules in order to allow the molecular motion.^47^ After the population transfer to S_0_, vibrational cooling originates the long-living component of ESA3 (“hot” ground state absorption, τ = 5.6 ps).

### 4.4 Disperse Blue 165

#### 4.4.1 Origin of the Transient Signals

**Figure 5:**
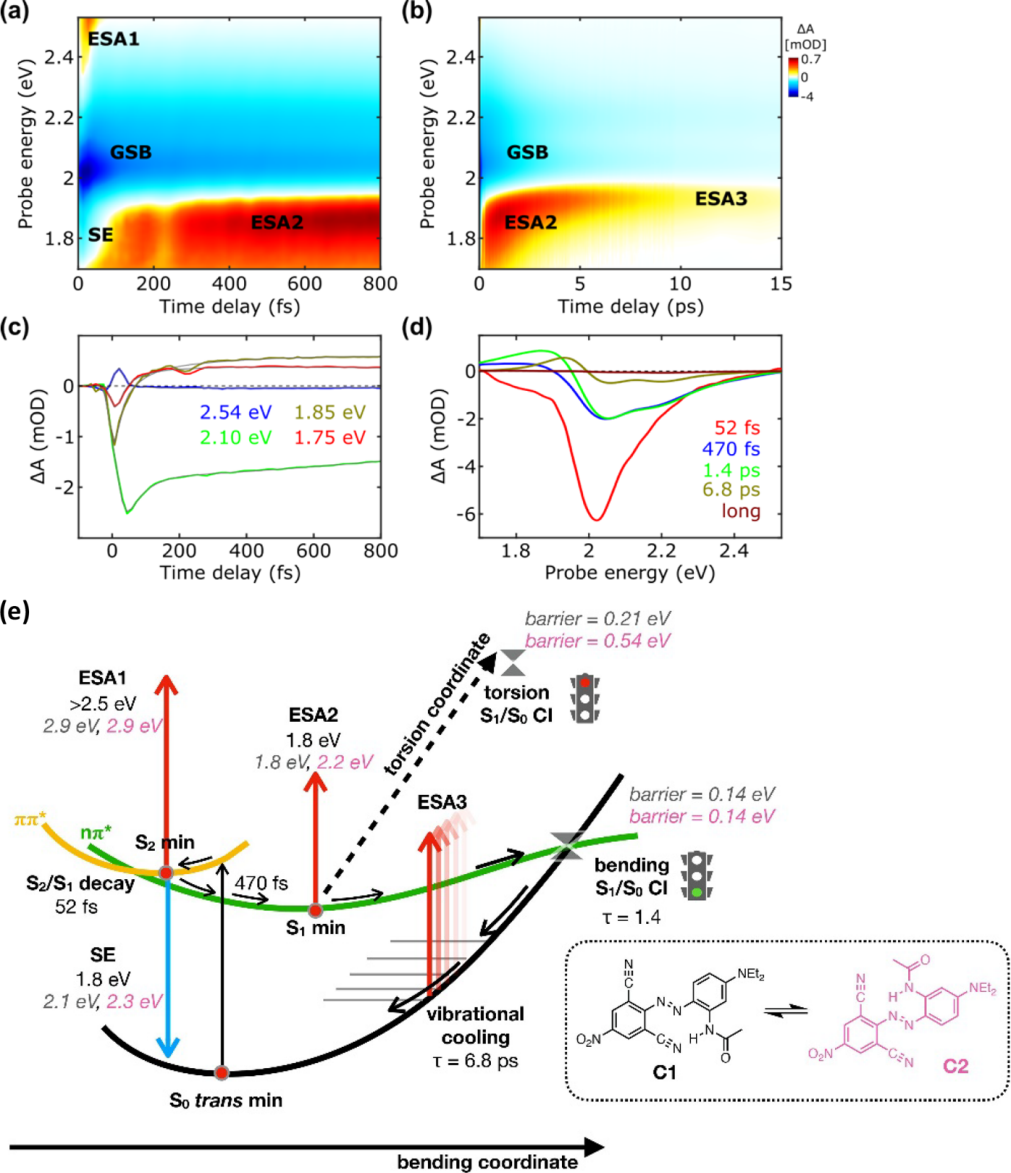
TA maps in the (a) first 800 fs and (b) until 15 ps of DB3GG. (c) Selected kinetic traces, focusing on the sub-800 fs dynamics, for selected probe wavelengths superimposed on the fittings obtained by global analysis. (d) The EAS and their corresponding time evolution as retrieved by global analysis. (e) A schematic representation of the deactivation mechanism is proposed for both C1 and C2 conformers based on the experimental observations (values in black) and computational results (gray, italics).

The linear absorption spectrum of DB165 (Figure 1d) shows a broad band peaking at 2.03 eV with a shoulder around 2.18 eV, both falling within the energy range of the pump spectrum. The energy of the absorption maximum is overestimated by all levels of theory (see vertical excitation energies of both conformers in Tables 5 and 6). Despite this, RASPT2 shows to be more accurate (with an error of 0.2–0.3 eV) and suggests that the main peak at lower energy might be associated with the S_0_ → S_2_ excitation in DB165-C1, while the higher energy shoulder could be originated by the S_0_ → S_2_ excitation in DB165-C2. In any case, both conformers can be excited by the pump pulse, and they will both be discussed in the following. The TA map (Figure 5a,b) shows the same trends and signals as DB366: an SE (1.85 eV) and an ESA (ESA1, visible as a tail around 2.45 eV) associated with the bright state that appear immediately after photoexcitation and disappear with a time constant of 52 fs, followed by the rise of ESA2 (1.75 eV). Similar to the vertical excitation, the SE energy (S_2_–S_0_ energy gap at the S_2_ minimum) is also overestimated both by RASPT2 and TDDFT. Concerning ESA1, only RASPT2 predicts a bright transition from S_2_ that could originate the detected tail (S_2_ → S_14_ in DB165-C1 and S_2_ → S_13_ in DB165-C2, see Tables 5 and 6), while no bright transitions were found by TDDFT. For both conformers, RASPT2 also predicts the presence of a second ESA at lower energy, but this is possibly buried under the very strong GSB signal. As observed in the previous cases, S_2_ and S_1_ are quite close already at the FC point (especially at the RASPT2 level, see Tables 5 and 6), and they get even closer at the S_2_ minima. Therefore, S_2_ is expected to decay to S_1_ soon after photoexcitation, in agreement with the SE and ESA1 ultrafast decays in the TA map.

**Table 5:**
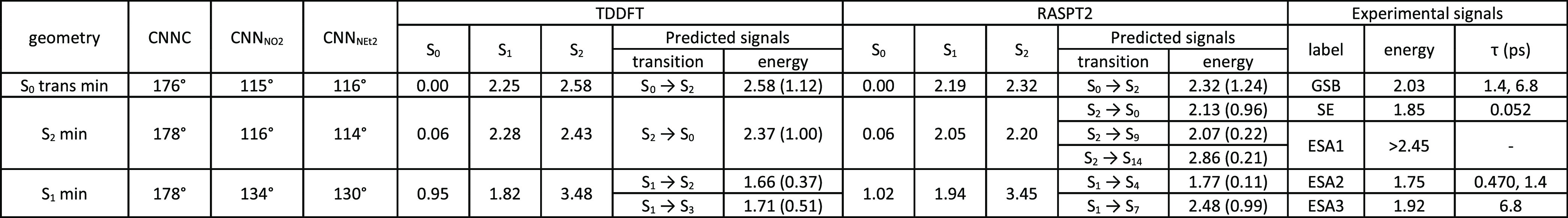
Relevant Geometrical Parameters and Electronic State Energies (in eV) at the QM/MM S_0_, S_2_, and S_1_ Minima of DB165-C1^a^

a The last two rows report the calculated bright transitions (energy in eV, oscillator strength in parentheses) and the corresponding peak position (eV) of the corresponding experimental signals (the associated time constants are reported in parentheses). For the S_2_ minimum, the QM/MM energy gaps were added to the S_0_ energy in PCM (relative to S_0_ trans min in PCM).

**Table 6:**
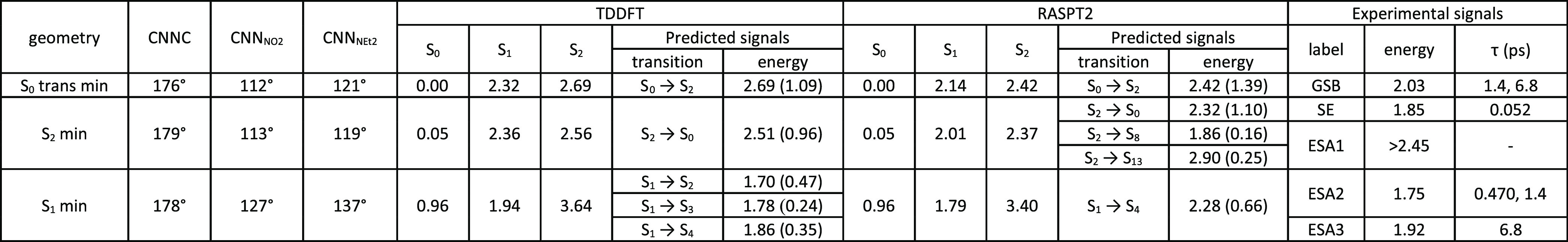
Relevant Geometrical Parameters and Electronic State Energies (in eV) at the QM/MM S_0_, S_2_, and S_1_ Minima of DB165-C2^a^

a The last two rows report the calculated bright transitions (energy in eV, oscillator strength in parentheses) and the corresponding peak position (eV) of the corresponding experimental signals (the associated time constants are reported in parentheses). For S_2_ and S_1_ minima, the QM/MM energy gaps were added to the S_0_ energy in PCM (relative to S_0_ trans min in PCM).

At later times, the population of S_1_ is associated with the rise of ESA2 (1.75 eV) that evolves with two time constants: a faster component of 470 fs and a slower component of 1.4 ps. Similar to DB366, the overall contribution of ESA2 likely arises from two distinct processes: (i) a peak initially centered around 1.77 eV that undergoes a blueshift toward 1.88 eV with a time constant of 470 fs (transition from blue to green colored EAS in Figure 5d). Importantly, there is no population recovery associated with this shift, indicating a relaxation across the S_1_ surface. (ii) A further blueshift of ESA2 that transforms gradually to the ESA3 peak located at 1.92 eV, corresponding to the “hot” ground state, which exhibits an intensity increase until approximately 1.5 ps (see Figure 5b), followed by decay with a time constant of 1.4 ps (transition from green to gold colored EAS in Figure 5d). Eventually, it completely disappears along with the GSB signal, accounting for approximately 99% of the initial bleached signal (Supporting Information). This observation implies the full recovery of the S_0_ trans population within this time frame.

Once again, the obtained S_1_ minima are planar structures with wide bending angles. From here, our calculations (both RASPT2 and TDDFT, see Tables 5 and 6) find a bright transition that matches very well with the experimental ESA2 signal. Only in the case of the S_1_ minimum in DB165-C1, a second bright transition is predicted at the border of the spectral window (S_1_ → S_7_ at 2.48 eV) that is not visible in the experiment. However, considering the computational error, this transition might fall outside the detection window.

#### 4.4.2 Presence and Accessibility of Conical Intersections

The presence of both the torsional and the bending deactivation pathways is confirmed by PCM calculations in both DB165 conformers (Table 2). Similar to DB366, both CIs lie at energies higher than the S_1_ minimum. However, while in DB165-C1 (and DB366, see before), the two crossings are almost isoenergetic (lying approximately 0.2 and 0.3 eV above the S_1_ minimum for bending and torsional CI, respectively), and the torsional CI is much destabilized in DB165-C2 compared to the bending one (approximately 0.2 vs 0.6 eV above the S_1_ minimum for bending and torsional CI, respectively). This is likely a consequence of the higher hydrogen bond strength of DB165-C2 with respect to C1 (see N–H distances in Figure 1d), which stabilizes more the planar geometry and increases the torsional barrier (see also CNN and CNNC scans in Figures S25–S28). These findings further suggest that the (unproductive) bending pathway is dominant in DB165, especially for most stable conformer 2.

#### 4.4.3 Deactivation Model

Figure 5e shows a scheme of the deactivation model proposed for DB165 (for both C2 and C1). The scenario is similar to that depicted for DB366 in all aspects, apart from the torsional barrier, which is more pronounced in DB165 (C2 in particular) due to the presence of a real and stronger H-bond (compared to the methyl H-bond of DB366).

## 5 Conclusions

The possibility to tune the photochemical and photophysical properties of molecules by engineering their structure allows the design of photoactive materials that fulfill key requirements for photocontrolled applications (e.g., photopharmacology, optoelectronics, etc.). In the present work, we have combined high-temporal-resolution (sub-15 fs) TA experiments with accurate QM/MM simulations to show how different kinds of substitutions affect the relative energies of the two decay pathways already known for the parent azobenzene molecule (i.e., unproductive symmetric bending vs productive torsion). The origin of the observed TA signals has been identified by means of static QM/MM calculations on the critical points on the ground and excited state PES, while the presence and accessibility of the bending/torsion pathways was determined combining QM/MM 0K dynamics with PCM calculations across the S_1_ PES.

The examined compounds span the space of possible substitutions, including a typical 4–4′ push–pull functionalization with an electron donor and an electron acceptor withdrawing groups (DNAB), a commercial dye in which one phenyl ring has been replaced with a naphthyl and in which the azo group is involved in keto–enol tautomerism (SRG) and two poly-substituted blue dyes (DB366 and DB165) showing an increasing strength of intramolecular hydrogen bonds that can stabilize the planar (trans) geometry.

Our results suggest that DNAB behaves exactly like other previously investigated push–pull derivatives:^23^ the ππ* stabilization brought about by the substituents reduces the S_2_ lifetimes, and at the same time, it reduces the amount of potential energy that can be transferred to symmetric bending oscillations, thus favoring the internal vibrational energy redistribution and the activation of the torsional motion more than in azobenzene. Photoisomerization is indeed observed for this compound, as testified by the GSB signal surviving for ≫1.2 ns. In the case of SRG, both keto and enol forms are expected to be present in the experimental mixture (almost barrierless proton transfer), but only the keto form is resonantly excited by the experimental pump pulse. This experimentally tracked keto tautomer shows an “improper” azo behavior, due to the different bond order with respect to a traditional azobenzene derivative. This causes the loss of the bending deactivation pathway, which suggests a further enhancement of the photoisomerization quantum yield. On the other hand, our simulations suggest that the enol-tautomer behaves similarly to DNAB and any other traditional push–pull azobenzene, with both bending and torsional decay pathways (torsion being more accessible than in the parent compound). Eventually, the study of Disperse Blue dyes suggests that the presence of an intramolecular hydrogen bond might stabilize the planar trans isomer enough to create a barrier along torsion that prevents isomerization (as testified by the GSB disappearance in both dyes). The barrier height might be tuned by increasing the hydrogen bond strength (e.g., replacing a methyl H-bond with a real H-bond).

## Supporting Information Available

Details on the preliminary calculations on all dyes, further details on the QM/MM setup, active space orbitals and RASPT2 details, QM/MM excitation energies calculated for the cis isomers, 0K dynamics, SRG-OH characterization, additional TA maps, disperse blue and SRG-NH potential energy scans, synthesis and purification of the DB366 and DB165 dyes, fluorescence quantum yield measurements, estimation of photoisomerization quantum yield from transient measurements, and Cartesian coordinates of the reported structures (PDF)
