# Supplementary material for: Engineering Azobenzene Derivatives to Control the Photoisomerization Process
Source: J Phys Chem A. 2023 Dec 5;127(49):10435–49. doi: 10.1021/acs.jpca.3c06108 (PMC10726365; doi:10.1021/acs.jpca.3c06108)
Supplement: Supplementary file 1 — jp3c06108_si_001.pdf [file jp3c06108_si_001.pdf]

Engineering Azobenzene Derivatives to Control the Photoisomerization Process

Flavia Aleotti[a], Vasilis Petropoulos[b], Hannah Van Overeem[c], Michele Pettini[d], Michele Mancinelli[a], Daniel Pecorari[a], Margherita Maiuri[b], Riccardo Medri[a], Andrea Mazzanti[a], Fabrizio Preda[e], Antonio Perri[e], Dario Polli[b,f], Irene Conti[a], Giulio Cerullo[b,f] and Marco Garavelli[a]

[a] Dipartimento di Chimica Industriale ``Toso Montanari", Università di Bologna, Viale del Risorgimento 4, 40136 Bologna, Italy
[b] Dipartimento di Fisica - Politecnico di Milano, Piazza Leonardo da Vinci 32, Milano, 20133, Italy
[c] van't Hoff Institute for Molecular Sciences, Universiteit van Amsterdam, Science Park 904, 1098 XH Amsterdam, The Netherlands
[d] Dipartimento di Chimica "Giacomo Ciamician", Università di Bologna, Via F. Selmi 2, 40126 Bologna, Italy
[e] NIREOS s.r.l, Via Giovanni Durando 39, 20158 Milan, Italy
[f] CNR - Institute for Photonics and Nanotechnologies (IFN), Piazza Leonardo da Vinci 32, 20133 Milan, Italy

SUPPORTING INFORMATION

CONTENTS

1) Preliminary calculations on the azo dyes
2) QM/MM setup details
3) RASSCF active space orbitals
4) Cis isomers: vertical excitation energies
5) Molecular dynamics at 0K
6) Characterization of SRG-OH dye
7) TA maps (long timescale)
8) Disperse blue dyes: bending and torsional scans (PCM)
9) Sudan Red G (NH tautomer): torsional scan
10) Synthesis and purification of DNAB and disperse blue dyes
11) Fluorescence quantum yield measurements
12) Relative estimation of the photoproduct yields from transient absorption measurements
13) Cartesian coordinates

1. PRELIMINARY CALCULATIONS

1.1. 4-DIETHYLAMINO-4'-NITROAZOBENZENE (DNAB)

Optimization of DNAB in methanol (PCM) with the relative orientation of the ethyl groups either *cis* (Figure S1a) or *trans* (Figure S1b) revealed these conformers to be almost isoenergetic (ΔE of 0.83 kcal/mol in favor of the *trans*-structure). Even though this indicates that both structures are present in the experimental mixture, it was chosen to solely investigate *trans*-DNAB with the ethyl groups orientated *trans* with respect to each other as it represents the most stable structure.

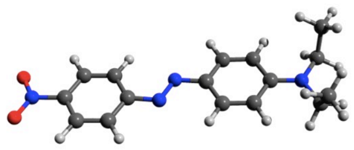

(a)

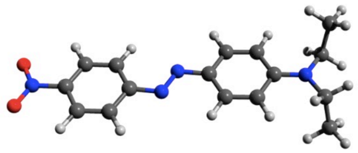

(b)

Figure S1. *cis* (a) and *trans* (b) disposition of the ethyl groups in DNAB.

1.2. SUDAN RED G

A preliminary investigation was conducted on SRG-OH and SRG-NH in order to determine the relative position of the methoxy substituent on the phenyl ring and the carbonyl/hydroxy group, respectively (compare Figure S2a with Figure S2b and Figure S2d with Figure S2e). The comparison of the M06/6-31G* energies in methanol (PCM) revealed that the relative *cis* orientation is more stable for both SRNG-NH (ΔE of 4.8 kcal/mol) and SRG-OH (ΔE of 3.5 kcal/mol). Secondly, after fixing the *cis* position of methoxy and hydroxy groups, the orientation of the latter in SRG-OH could either form an intramolecular hydrogen-bond with one of the central nitrogen atoms (Figure S2a), or with a solvent molecule (Figure S2c). Optimization of the QM/MM systems (using the setup described in the computational details) showed that the intramolecular hydrogen-bonding network results in a stabilization of the chromophore of 5 kcal/mol compared

to the intermolecular network. Based on these results, the (*trans*-)SRG-OH and (*trans*-)SRG-NH are defined as the planar structures illustrated in Figure S2a and Figure S2d, respectively.

In order to investigate the relative abundance of SRG-NH and SRG-OH in the experimental sample, the stability of the two structures was compared. The energy difference for the two optimized tautomers in methanol (PCM) at the MP2/6-31G* level was found to be of 2.19 kcal/mol in favor of the NH structure. In addition, a rigid scan along the hydrogen shift coordinate in PCM indicates the hydrogen-transfer barrier to be only 0.54 kcal/mol (which is probably an upper estimate, considering that other degrees of freedom were not relaxed). This suggests that SR interchanges easily between SRG-NH and the SRG-OH at room temperature, and it is therefore assumed that both tautomers are present in the experimental mixture. As the signals from the spectroscopy experiments could be (a mix of) fingerprints of both structures, it was chosen to further investigate the excited-state characteristics of both.

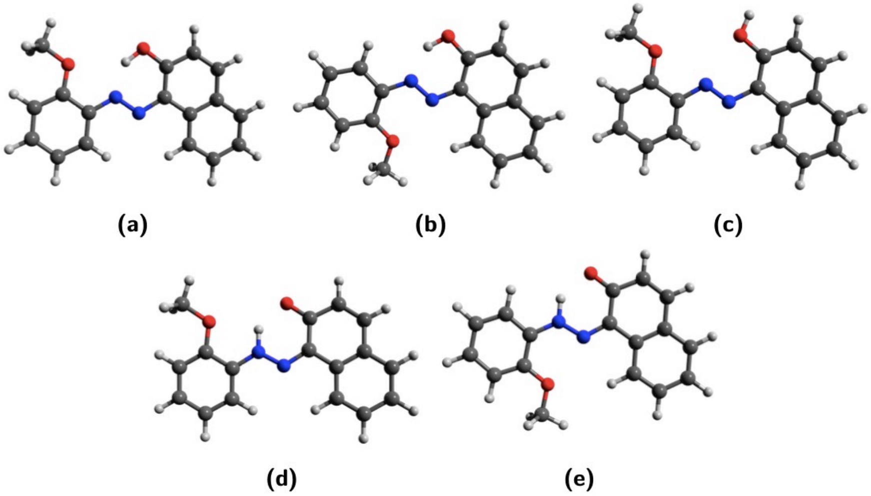

Figure S2. Possible conformers for *trans*-SRG-OH (a-c) and *trans*-SRG-NH (d,e).

1.3. DISPERSE BLUE 366

The correct conformer of *trans*-DB366 was chosen from a geometry analysis resulting from DFT dynamic computations on more than 20 possible conformational isomers. A scan of all possible dihedrals angles has been done running at the B3LYP/6-31G(d) level of theory. The geometries that have energy lower than 2 kcal/mol were taken into consideration.

1.4. DISPERSE BLUE 165

Refining the two conformers ground state minimum energies by DFT optimization in implicit solvent (PCM) we found that the C2 conformer is more stable respect to C1 by just 0.16 eV (3.60 kcal/mol). A scan of all possible dihedrals angles has been done running at the B3LYP/6-31G(d) level of theory. The geometries that have energy lower than 2 kcal/mol were taken into consideration. As solvent was used chloroform.

2. QM/MM SETUP - FURTHER DETAILS

The setup of the methanol droplets containing the various chromophores was accomplished in 3 steps using the automated tools implemented in the suite COBRAMM[1] as described in the main text. It was found that the default GAFF2 force field[2] (FF) used by AMBER[3] in the MM/MD steps performed insufficient, underestimating the rotational barrier of the central CNNC and of the two CCNN dihedrals, resulting in partially rotated (incorrect) structures for the optimized solvated chromophores. The inaccuracy of the rotated structures was assessed based on the observations that, if the setup was repeated forcing the chromophores to remain planar (i.e., keeping them frozen at their gas-phase QM optimized structure), the final droplets were always more stable, and also showed vertical excitation energies in better agreement with the experimental linear absorption. Therefore, by comparison with an accurate in-house parametrized azobenzene FF available in the research group, the following parameters were modified in the FF: the rotational barrier of the central CNNC dihedral was increased from 2.8 kcal/mol to 30 kcal/mol, and the barrier of the CCNN dihedrals was raised from 0 kcal/mol to 1.7 kcal/mol. In the case of SRG-NH, the system cannot be well described by the adjusted FF developed for classical azo-compounds, because the central nitrogen atoms are connected via a single bond. For that reason, it was chosen to keep SRG-NH frozen at its gas-phase QM optimized geometry during the entire QM/MM set up procedure. All DB dyes were also kept frozen at their PCM optimized geometry during the QM/MM setup.

3. RASSCF ACTIVE SPACE AND TYPE OF RASPT2 CORRECTION

For all RASSCF calculations (independent of the active space size) all the active orbitals which are doubly occupied in the HF configuration were placed in RAS1, while all HF virtual orbitals were placed in RAS3, allowing a maximum of 4 excitations. This "empty RAS2" technique has already been used and validated both for azobenzene and for its push-pull derivatives.[4,5] The active space composition, number of RASSCF roots as well as type of RASPT2 correction is discussed in the following for each compound.

3.1. 4-DIETHYLAMINO-4'-NITROAZOBENZENE

All the RASPT2/RASSCF calculations on DNAB were performed using an active space made up of 24 electrons in 19 orbitals (see Figure S3). The RASSCF state-averaging included the lowest six roots in all calculations except for the S_1 minimum geometry, where the need to evaluate a possible ESA signal required the calculation over 10 roots.

Concerning the RASPT2 correction, both single state (SS), multistate (MS) and extended multistate (XMS) RASPT2 energies were evaluated. Eventually, SS-RASPT2 results showed to give predictions in best agreement with the experimental signals (therefore, all RASPT2 results reported in the main text correspond to SS-RASPT2 values).

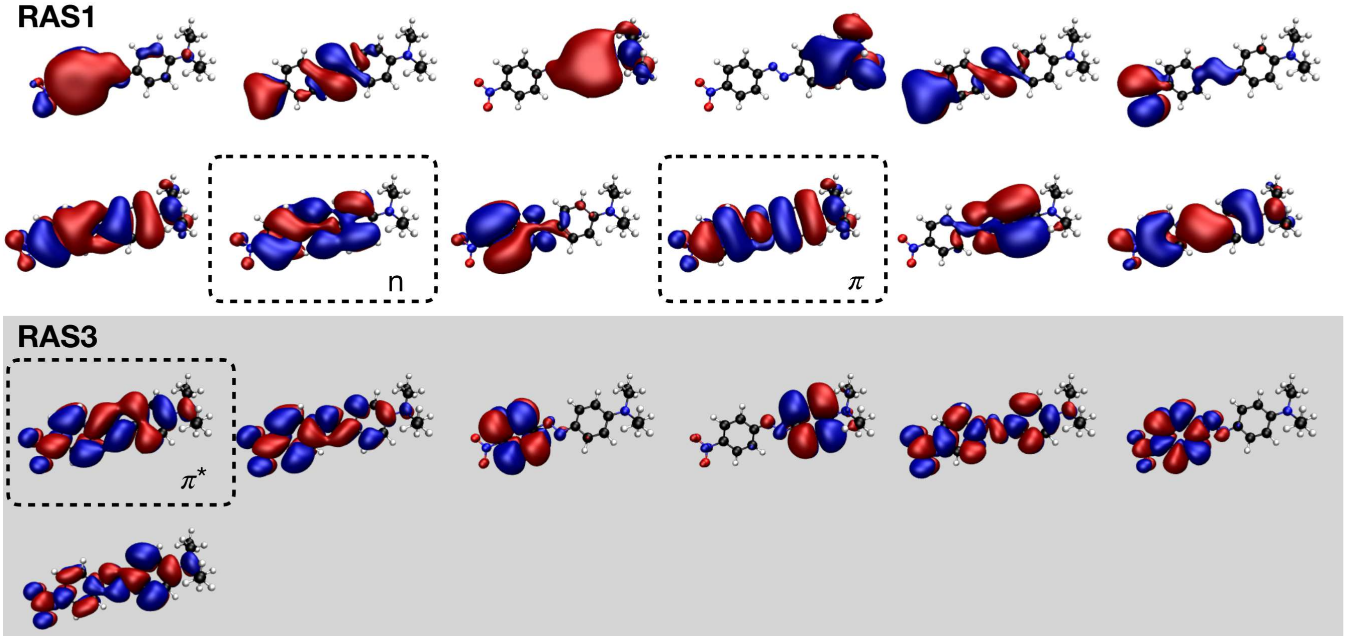

Figure S3. SA6-RASSCF(4,12|0,0|4,7)/6-31G* active space orbitals for DNAB in its S_0 *trans* minimum. Corresponding orbitals were used for all other geometries.

3.2. SUDAN RED G – NH TAUTOMER

All the RASPT2/RASSCF calculations on SRG-NH were performed using an active space made up of 24 electrons in 20 orbitals (see Figure S4). The RASSCF state-averaging included the lowest six roots in all calculations except for S_1, T_1 and T_2 minima, where the need to evaluate a possible ESA signals required the calculation over a different number of roots: 20 singlet states at the S_1 minimum geometry, 15 triplet states at the T_1 and T_2 minima. Only at the S_1 minimum, we performed two additional RASSCF/RASPT2 calculations using the same active space but averaging over only two roots (one calculation for singlet states and one for triplet states): the resulting wavefunctions were used to calculate the spin-orbit coupling and estimate the $S_1 \rightarrow T_2$ ISC probability.

Concerning the RASPT2 correction, both single state (SS), multistate (MS) and extended multistate (XMS) RASPT2 energies were evaluated. Eventually, SS-RASPT2 results showed to give predictions in best agreement with the experimental signals (therefore, all RASPT2 results reported in the main text correspond to SS-RASPT2 values).

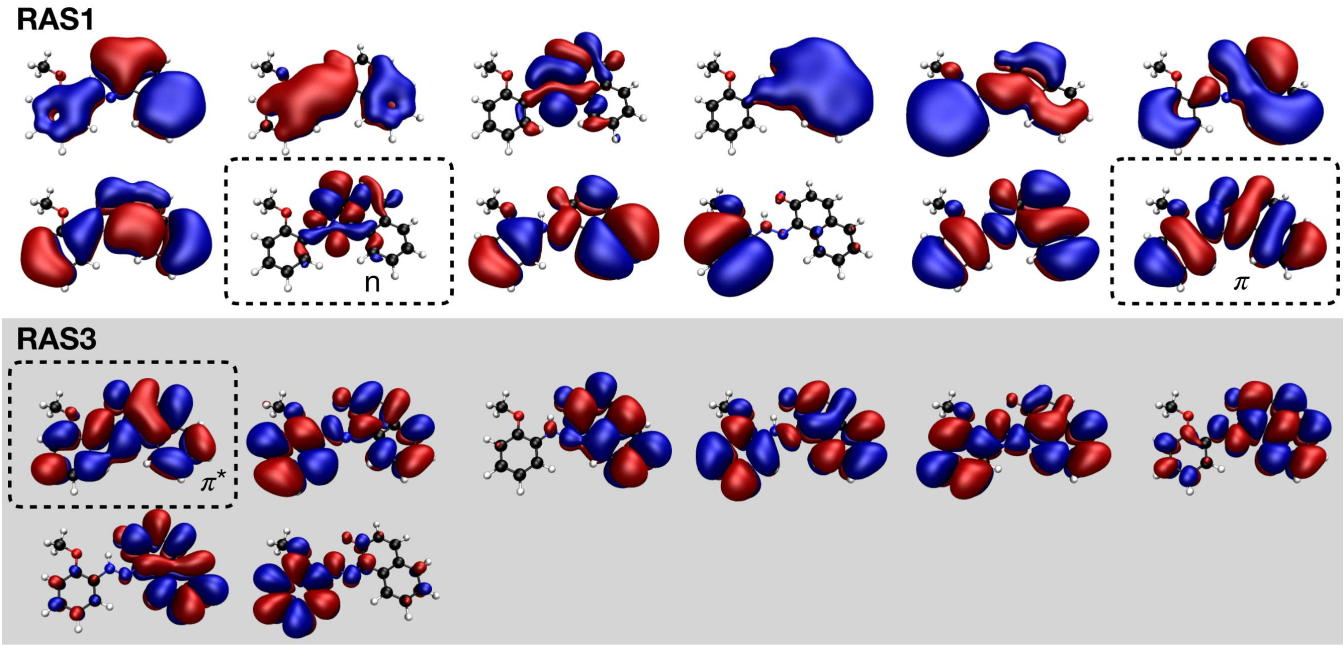

Figure S4. SA6-RASSCF(4,12|0,0|4,8)/6-31G* active space orbitals for SRG-NH in its S_0 *trans* minimum. Corresponding orbitals were used for all other geometries.

3.3. SUDAN RED G – OH TAUTOMER

All the RASPT2/RASSCF calculations on SRG-OH were performed using an active space made up of 22 electrons in 19 orbitals (see Figure S5). The RASSCF state-averaging included the lowest six roots in all calculations except for the S_1 minimum geometry, where the need to evaluate a possible ESA signal required the calculation over 15 roots. At the same geometry, also a SA4 calculation over triplet states was performed using the same active space.

Concerning the RASPT2 correction, both single state (SS), multistate (MS) and extended multistate (XMS) RASPT2 energies were evaluated. Eventually, SS-RASPT2 results showed to give predictions in best agreement with the experimental signals (therefore, all RASPT2 results reported in the main text correspond to SS-RASPT2 values).

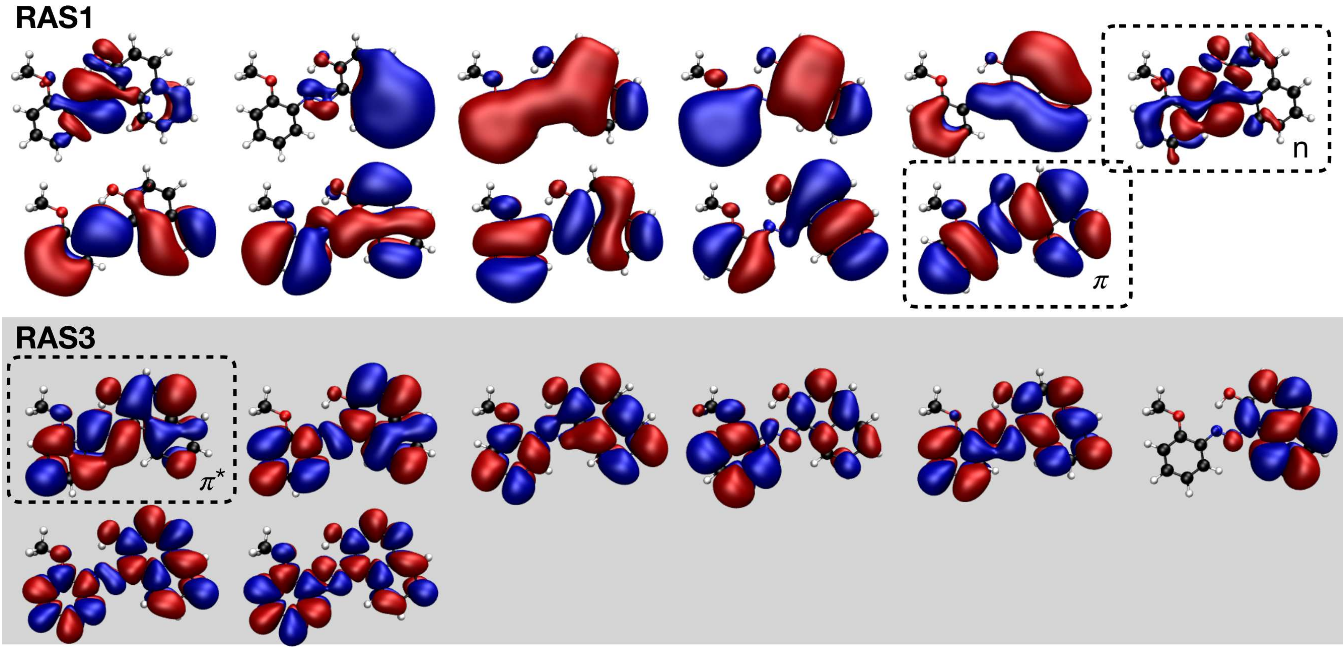

Figure S5. SA6-RASSCF(4,11|0,0|4,8)/6-31G* active space orbitals for SRG-OH in its S_0 *trans* minimum. Corresponding orbitals were used for all other geometries.

3.4. DISPERSE BLUE 366

All the RASPT2/RASSCF calculations on DB366 were performed using an active space made up of 18 electrons in 16 orbitals (see Figure S6). The RASSCF state-averaging included the lowest six roots in all calculations except for S_1 and S_2 minima, where the need to evaluate a possible ESA signals required the calculation over 15 roots.

Concerning the RASPT2 correction, both single state (SS), multistate (MS) and extended multistate (XMS) RASPT2 energies were evaluated. Eventually, SS-RASPT2 showed to be the most accurate far from strong coupling regions (and was eventually chosen for vertical transition energies at the FC point), while MS-RASPT2 was more accurate elsewhere.

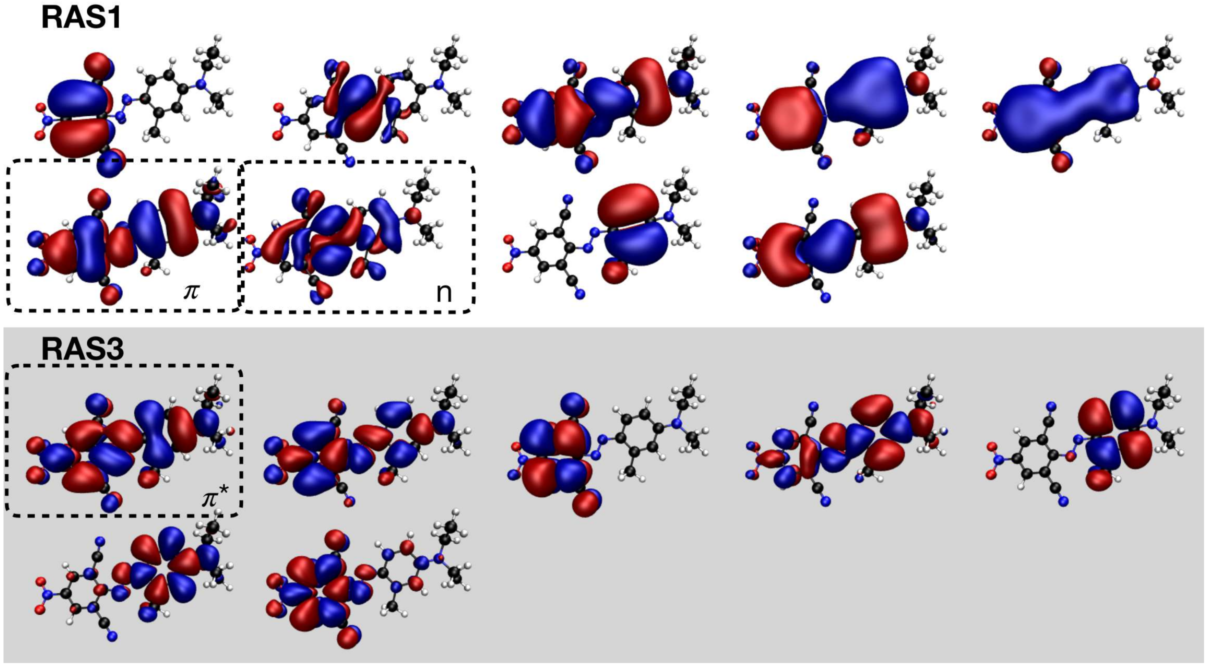

Figure S6.SA6-RASSCF(4,9|0,0|4,7)/6-31G* active space orbitals for DB366 in its S_0 *trans* minimum. Corresponding orbitals were used for all other geometries

3.5. DISPERSE BLUE 165 – CONFORMER 1

All the RASPT2/RASSCF calculations on DB165-C1 were performed using an active space made up of 18 electrons in 16 orbitals (see Figure S7). The RASSCF state-averaging included the lowest six roots in all calculations except for S_1 and S_2 minima, where the need to evaluate a possible ESA signals required the calculation over 15 roots.

Concerning the RASPT2 correction, both single state (SS), multistate (MS) and extended multistate (XMS) RASPT2 energies were evaluated. Eventually, MS-RASPT2 results showed to give predictions in best agreement with the experimental signals (therefore, all RASPT2 results reported in the main text correspond to MS-RASPT2 values).

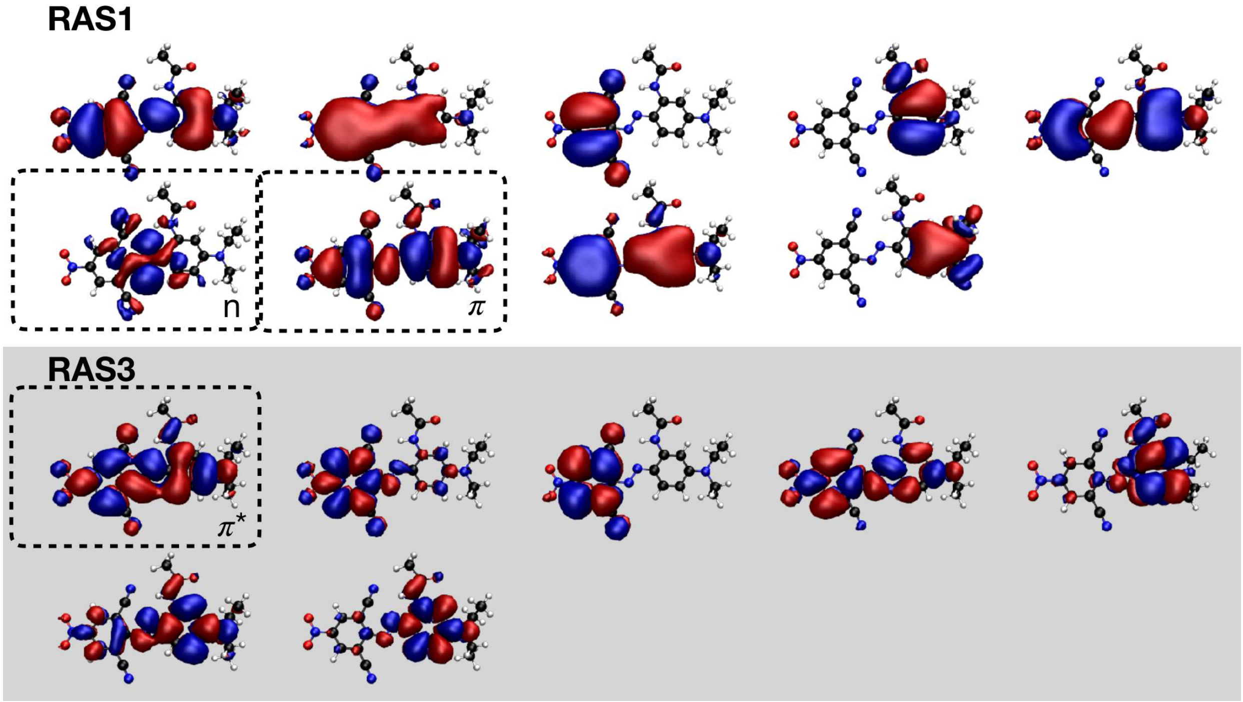

Figure S7.SA6-RASSCF(4,9|0,0|4,7)/6-31G* active space orbitals for DB165-C1 in its S_0 *trans* minimum. Corresponding orbitals were used for all other geometries

3.6. DISPERSE BLUE 165 – CONFORMER 2

All the RASPT2/RASSCF calculations on DB165-C2 were performed using an active space made up of 18 electrons in 16 orbitals (see Figure S8). The RASSCF state-averaging included the lowest six roots in all calculations except for S_1 and S_2 minima, where the need to evaluate a possible ESA signals required the calculation over 15 roots.

Concerning the RASPT2 correction, both single state (SS), multistate (MS) and extended multistate (XMS) RASPT2 energies were evaluated. Eventually, MS-RASPT2 results showed to give predictions in best agreement with the experimental signals (therefore, all RASPT2 results reported in the main text correspond to MS-RASPT2 values).

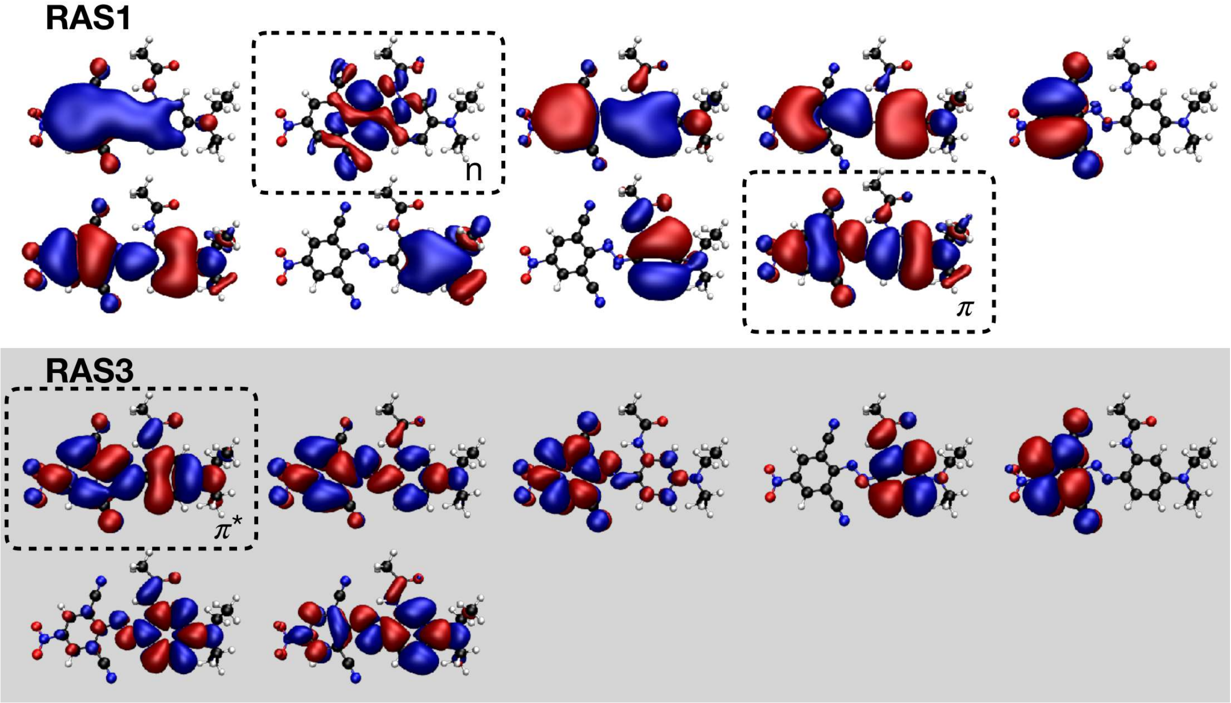

Figure S8. SA6-RASSCF(4,9|0,0|4,7)/6-31G* active space orbitals for DB165-C2 in its S_0 *trans* minimum. Corresponding orbitals were used for all other geometries

4. GROUND STATE CIS ISOMERS

Table S1. QM/MM vertical excitation energies (relative to the corresponding *cis* S_0 isomer) at the *cis* S_0 minimum (AMBER/M06/6-31G* optimization) for DNAB, SRG-NH and SRG-OH.

		DNAB		SRG-NH		SRG-OH	
		energy	f	energy	f	energy	f
TD-DFT	S_1	2.57	0.04	2.39	0.04	2.58	0.04
	S_2	2.91	0.02	3.00	0.16	3.42	0.03
	S_3	3.29	0.03	3.34	0.03	3.67	0.06
	S_4	3.81	0.06	3.60	0.04	3.97	0.04
	S_5	4.02	0.06	3.95	0.01	4.13	0.02
RASPT2	S_1	2.93	0.01	2.26	0.01	2.85	0.02
	S_2	3.68	0.03	3.33	0.04	3.75	0.02
	S_3	4.08	0.02	3.38	0.04	3.92	0.00
	S_4	4.42	0.03	3.55	0.04	4.14	0.01
	S_5	4.44	0.06	4.79	0.02	4.47	0.04

5. DYNAMICS AT 0 KELVIN

Table S2 - Geometrical parameters and energy gaps between the involved electronic states at the hopping geometries from the TDDFT 0K dynamics of the considered structures.

	S_2/S_1 decay					S_1/S_0 decay				
	CNNC	$CNN_{(a)}$	$CNN_{(b)}$	TDDFT gap (eV)	RASPT2 gap (eV)	CNNC	$CNN_{(a)}$	$CNN_{(b)}$	TDDFT gap (eV)	RASPT2 gap (eV)
DNAB	174°	115°	112°	0.09	0.09	177°	139°	139°	0.13	0.14
SR-NH	-	-	-	-	-	99°	123°	114°	0.04	0.42
DB366	177°	115°	116°	0.06	0.51	-	-	-	-	-
DB165-C1	179°	118°	116°	0.09	0.22	-	-	-	-	-
DB165-C2	173°	113°	118°	0.06	0.03	-	-	-	-	-

DNAB, DB366, DB165-C1, DB165-C2: (a) = NO2, (b) = NEt2; SRG-NH: (a) = Ph, (b) = napth.

5.1. 4-DIETHYLAMINO-4'-NITROAZOBENZENE (DNAB)

Figure S9 shows the electronic state energies and the most relevant geometrical parameters along the nuclear 0K trajectory, while details about the decay points are collected in Tab. The TDDFT trajectory initially follows some in-plane deformations, which are mostly addressable to small symmetric CNN_{NO2}/CNN_{NEt2} bending oscillations. These lead to the $S_2 \rightarrow S_1$ crossing after 173 fs, in agreement with the experimental se disappearing in 80 fs (considering that this is an upper estimate of the real lifetime due to the lack of initial momentum). The S_2/S_1 crossing geometry is very similar to the S_2 minimum identified by geometry optimization. From this point, the trajectory moves towards the planar S_1 minimum, as clearly indicated by the wide symmetric bending oscillations triggered by the decay to S_1 (Figure S9). The trajectory then moves on the S_1 surface until 803 fs, when it reaches a planar crossing point with S_0 characterized by wide CNN_{NO2}/CNN_{NEt2} angles (Tab). This "in-plane" deactivation channel belongs to the unproductive part of the S_1/S_0 crossing seam in azo compounds[4,6–8] and represents a faster, unproductive alternative to the slower torsional mechanism (leading to isomerization).[4,6,9] Although the lack of initial momentum and of a statistical trajectory ensemble does not allow to compare the decay time to the experimental S_1 time constants (210, 560 fs), the TDDFT decay geometry was validated by RASPT2 calculations (Tab), confirming the presence of the bending decay path. As previously mentioned, some preliminary studies suggested that the (slower) productive torsional route might be favored in push-pull azobenzenes[5] (due to the lower initial potential energy of the S_2 state, which reduces the amplitude of the bending oscillations leading to the planar crossing). Although the 0K trajectory did not show it, the presence of the torsional crossing at lower energy was confirmed by subsequent PSM calculations (see main text).

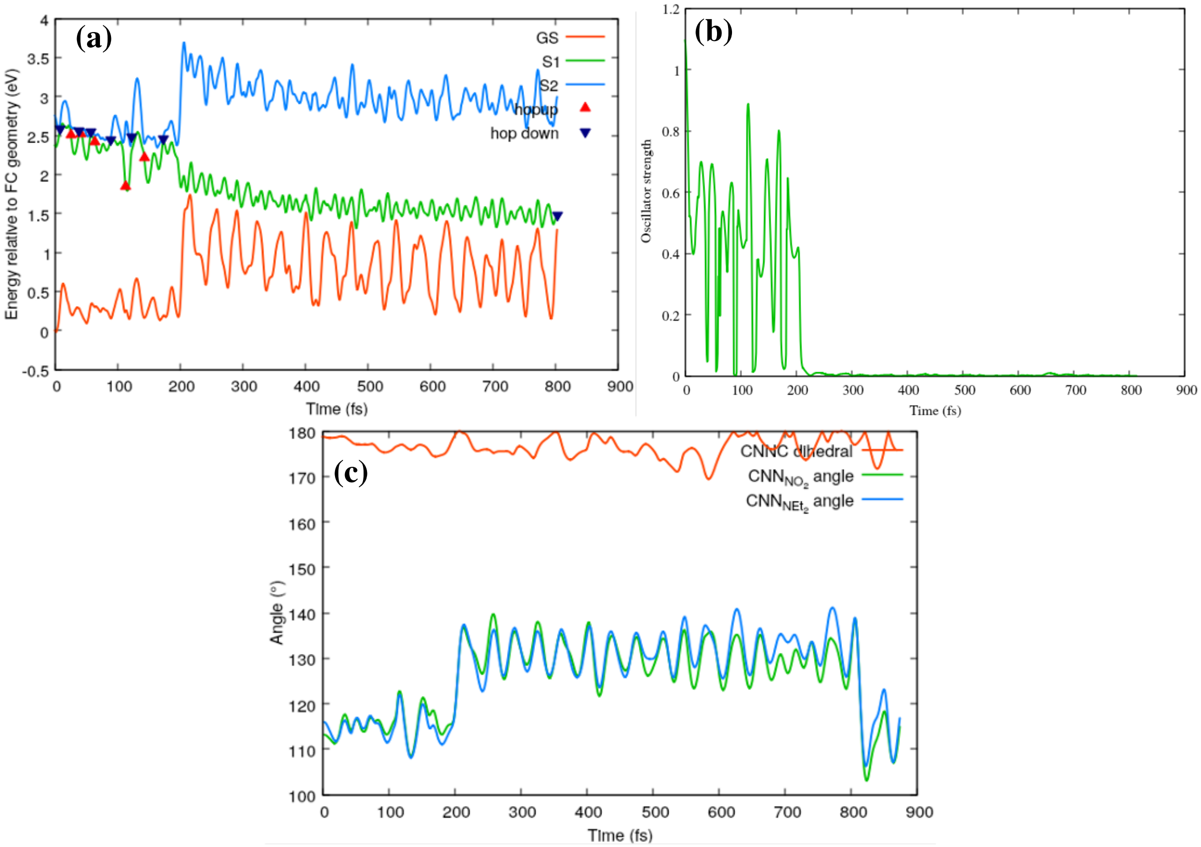

Figure S9. 0K dynamics of excited *trans*-DNAB in methanol (QM/MM): (a) Energy of S_0, S_1 and S_2 relative to S_0 at the FC geometry. Transitions to lower-lying and higher-lying states are illustrated with blue triangles pointing down and red triangles pointing up, respectively. (b) Oscillator strength between the active state and S_0. (c) CNN_{NO2} angle (green), CNN_{NEt2} angle (blue) and CNNC dihedral (orange).

5.2. SUDAN RED G – NH TAUTOMER

Figure S10 shows the electronic state energies and the most relevant geometrical parameters along the nuclear 0K trajectory, while details about the decay points are collected in Table S2. After photoexcitation, SRG-NH moves on S_1 for the few hundreds of fs before the S_1/S_0 crossing region is visited. The torsional motion is immediately activated from the FC point, confirming that the CNNC rotation is leading the S_1 gradient towards the S_1 minimum. However, the torsional momentum gained in the dynamics brings the molecule past the minimum geometry, towards a fully-rotated S_1/S_0 CI from which it decays on S_0 in 538 fs (see geometrical parameters in Table S2). Indeed, a relaxed scan along the CNNC torsion coordinate (Figure S29) showed that the S1 PES is substantially flat between the S_1 minimum and CNNC = 130°, with only a very small barrier caused by the elongation of the intramolecular H bond. Past 130° torsion, the H-bond is broken, causing a high S_0 destabilization and leading to the S_1/S_0 crossing. The torsional decay pathway identified by this exploratory trajectory endorses the hypothesis of photoisomerization with formation of the cis isomer (GSB survival). On the other hand, the impossibility to include ISC and triplet states in the dynamics does not allow to confirm the triplet hypothesis (long living ESA2).

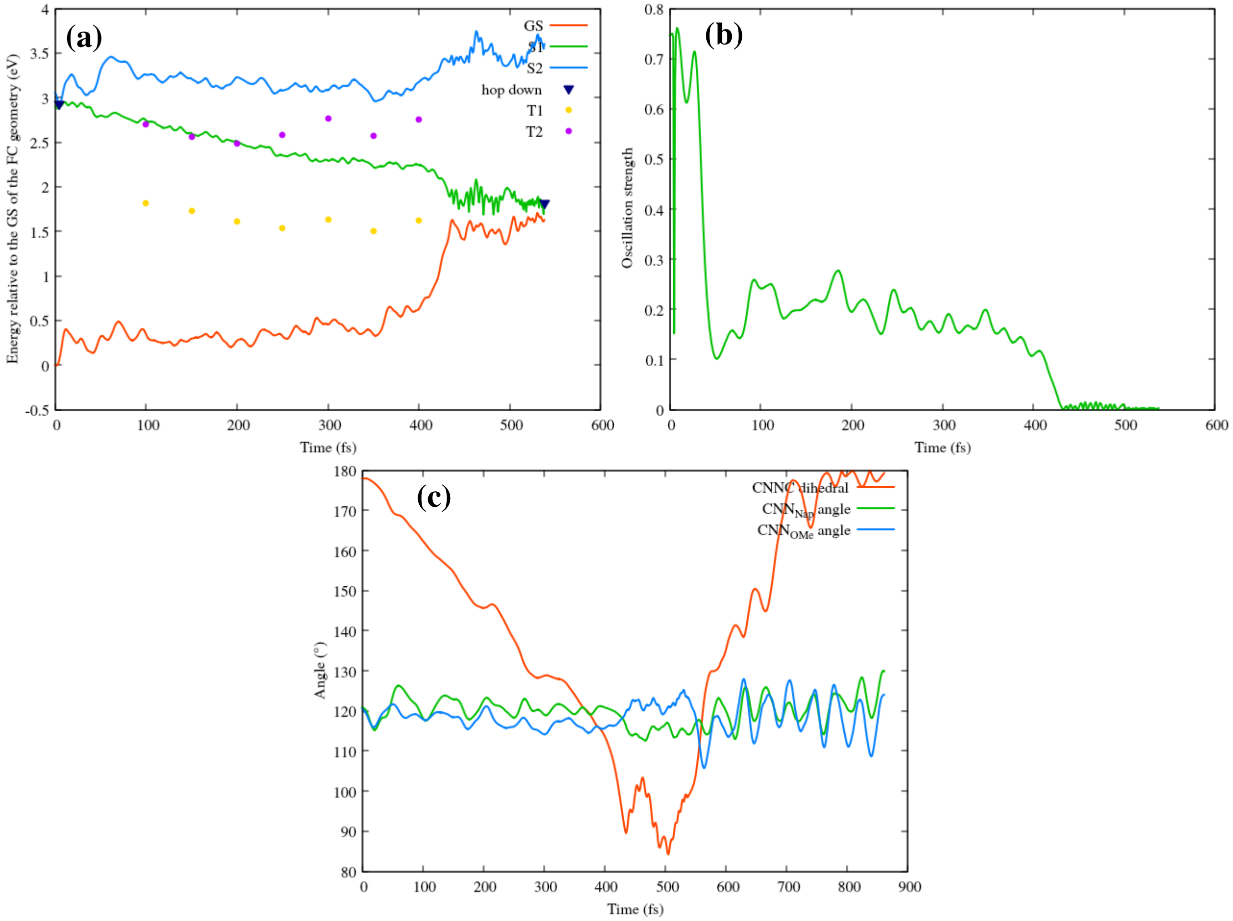

Figure S10. 0K dynamics of excited *trans*-SRG-NH in methanol (QM/MM): (a) Energy of S_0, S_1 and S_2 relative to S_0 at the FC geometry. The energy of T_1 and T_2 are also reported at regular intervals along the trajectory. Transitions to lower-lying states are illustrated with blue triangles pointing down. (b) Oscillator strength between the active state and S_0. (c) CNNC dihedral (orange), CNN_{napht} angle (green), and CNN_{Ph} angle (blue).

5.2. SUDAN RED G – OH TAUTOMER

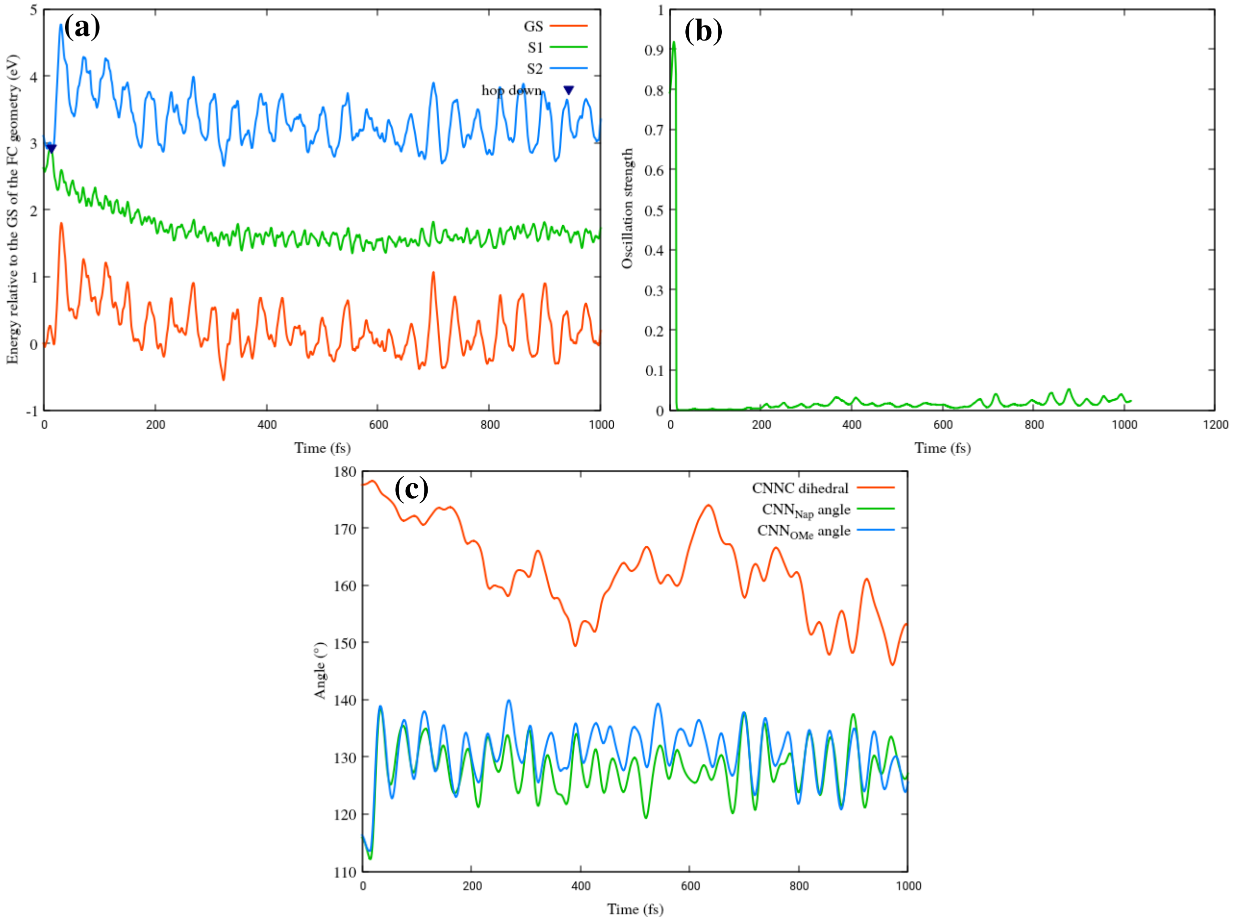

Figure S11. 0K dynamics of excited *trans*-SRG-OH in methanol (QM/MM): (a) Energy of S_0, S_1 and S_2 relative to S_0 at the FC geometry. Transitions to lower-lying states are illustrated with blue triangles pointing down. (b) Oscillator strength between the active state and S_0. (c) CNNC dihedral (orange), CNN$_{napht}$ angle (green), and CNN$_{Ph}$ angle (blue).

5.2. DISPERSE BLUE 366

Figure S12 shows the electronic state energies and the most relevant geometrical parameters along the nuclear 0K trajectory, while details about the decay points are collected in Table 2. The trajectory was started at the FC point on the bright S_2 state, and it hops down to S_1 after 17 fs close to the S_2 minimum (compare geometrical parameters in Table 2 and Table 5). Afterwards, it resides on S_1 until the end of the simulation (1 ps). Although no hop to S_0 is observed, it is interesting to notice that the CNNC dihedral remains close to planarity for the full simulation time (oscillating between 170° and 190°) and that the $S_2 \rightarrow S_1$ decay triggers wide and symmetric oscillations of the CNN/NNC angles. These findings further endorse the hypothesis that the bending pathway is preferred in DB366.

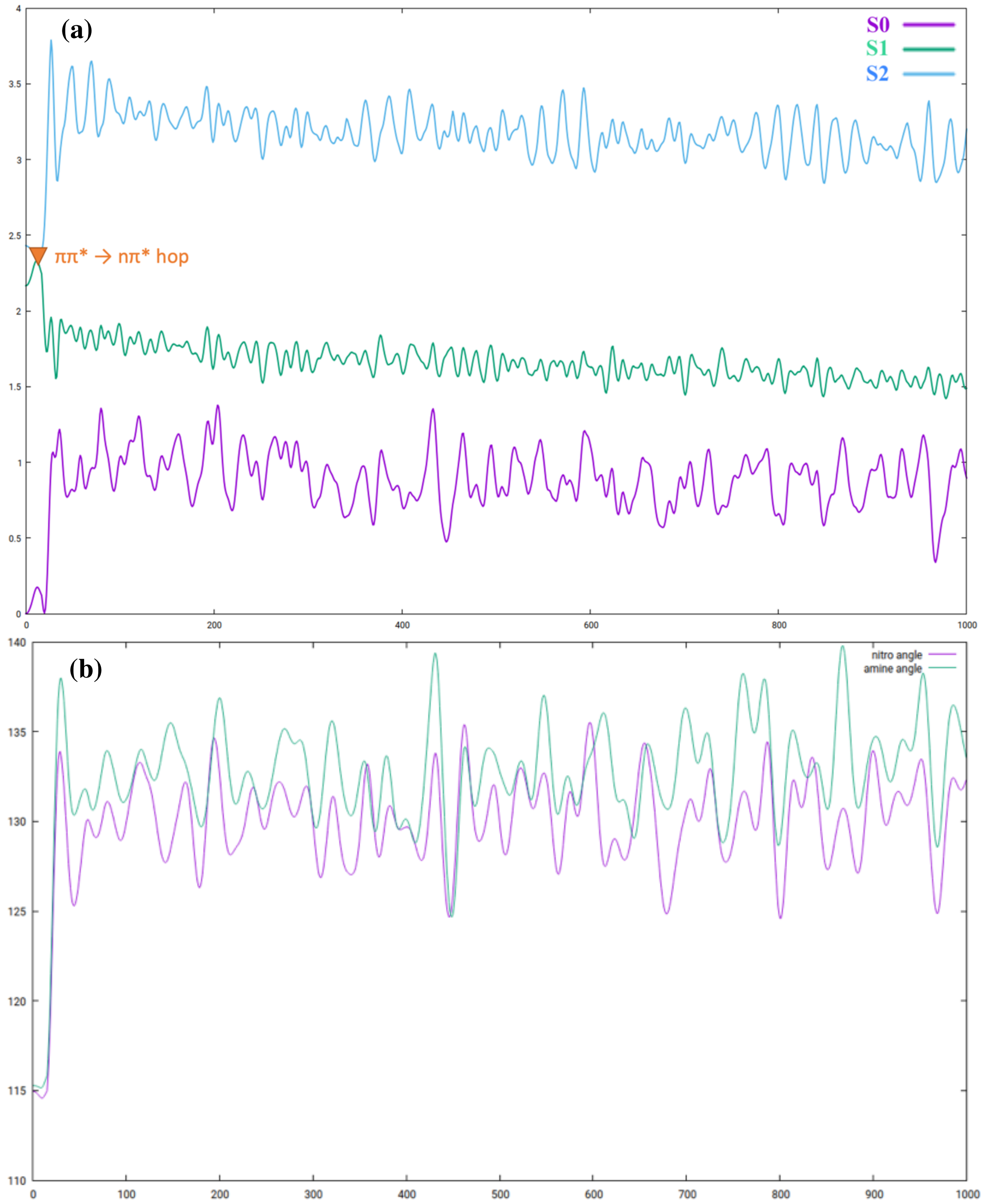

Figure S12. 0K dynamics of excited *trans*-DB366 in methanol (QM/MM): (a) Energy of S_0, S_1 and S_2 relative to S_0 at the FC geometry. Transitions to lower-lying states are illustrated with orange triangles pointing down. (b) CNN_{NO2} angle (violet), and CNN_{NEt2} angle (green).

5.2. DISPERSE BLUE 165

Figures S13 and S14 shows the electronic state energies and the most relevant geometrical parameters along the nuclear 0K trajectory, while details about the decay points are collected in Table 2. The dynamical behavior of the two DB165 is qualitatively similar to that observed for DB366, with an initial S_2/S_1 hop, followed by planar and symmetric bending oscillations on S_1 until the end of the simulation (1 ps). However, in contrast to DB366, the decay to S_1 is observed at longer times (17, 94 and 430 fs for DB366, DB165-C1 and C2, respectively). Considering that the S_2-S_1 gap at the FC point for the two DB165 dyes is overestimated by TDDFT (compared to RASPT2, see Table 6 and Table 7 in main text), the S_2/S_1 decay time is expected to be overestimated. As discussed for DB366, the retained planarity and the wide, symmetric bending oscillations triggered by S_2/S_1 decay suggest that the "bending path" (no photoisomerization) could be favored also in DB165, in agreement with the GSB disappearance in the TA map.

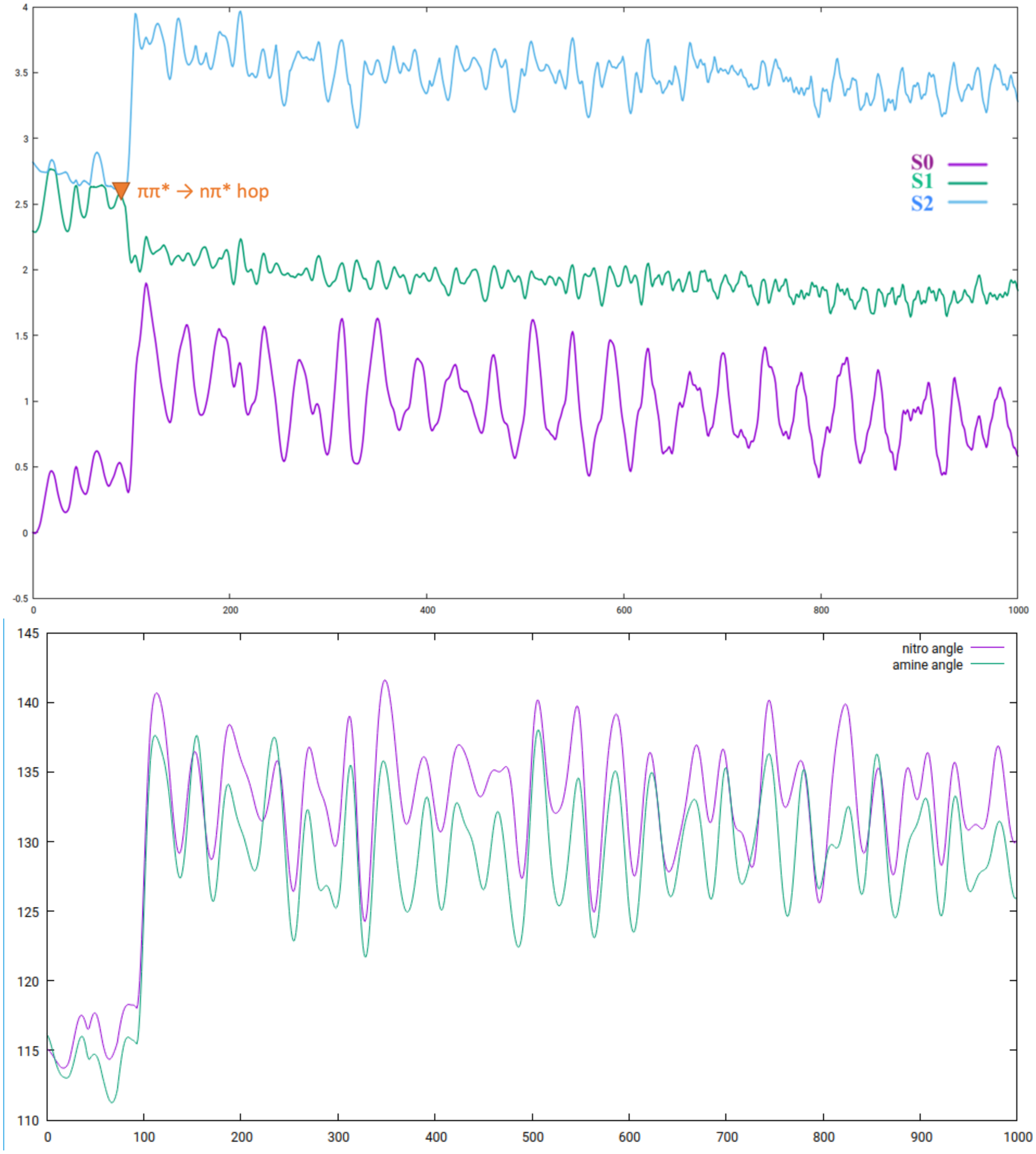

Figure S13. 0K dynamics of excited *trans*-DB165-C1 in methanol (QM/MM): (a) Energy of S_0, S_1 and S_2 relative to S_0 at the FC geometry. Transitions to lower-lying states are illustrated with orange triangles pointing down. (b) CNN_{NO2} angle (violet), and CNN_{NEt2} angle (green).

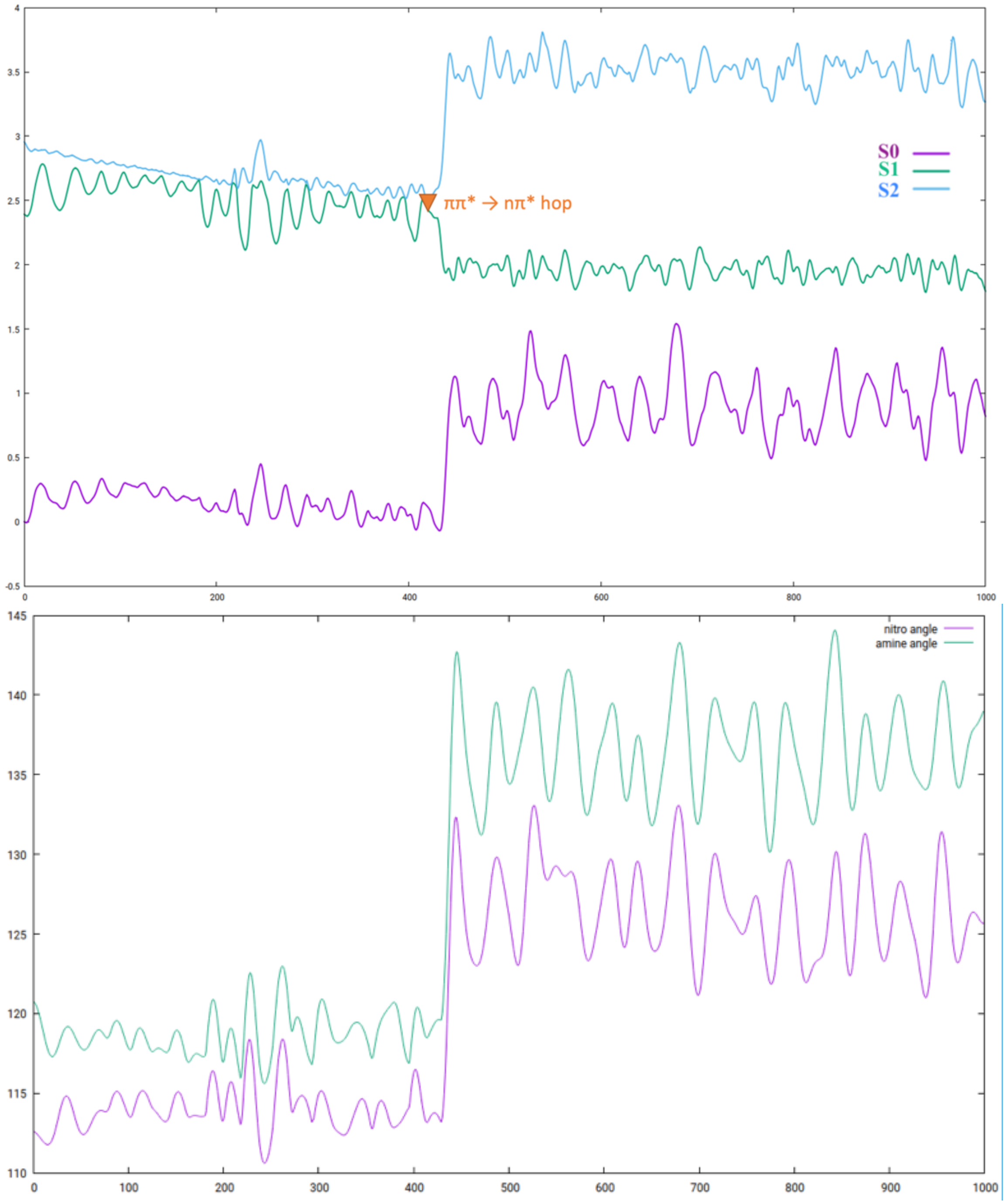

Figure S14. 0K dynamics of excited *trans*-DB165-C2 in methanol (QM/MM): (a) Energy of S_0, S_1 and S_2 relative to S_0 at the FC geometry. Transitions to lower-lying states are illustrated with orange triangles pointing down. (b) CNN_{NO2} angle (violet), and CNN_{NEt2} angle (green).

6. CHARACTERIZATION OF SUDAN RED G – OH ISOMER

The bright state of SRG-OH is S_2 ($\pi\pi^*$), whose TDDFT optimization rapidly leads to a crossing with S_1 (dark, $n\pi^*$) very close to the FC point (see geometrical parameters in Table S3). The presence of this crossing rules out the possibility to address the long living SE signal to this species. The minimum on S_1 could originate longer-living signals, but this state is not bright (excluding the possibility to observe SE). Moreover, no bright transitions matching the experimental ESA1 and ESA3 (showing the same dynamics as SE) were identified.

The probability of ISC from S_1 to T_2 is also very low in SRG-OH, both because of the large energy gap between the states and because of the low SOC (Table S3).

Table S3. Relevant geometrical parameters and electronic state energies (eV, relative to S_0 at the corresponding structure) at the QM/MM S_0, S_2 and S_1 minima of SRG-OH. The SOC magnitude from S_1 is also reported in parentheses for triplet states. The last two rows report the calculated bright transitions (eV, oscillator strength in parentheses) and the corresponding experimental signal (eV).

	S_0 min		S_2 min		S_1 min	
CNNC	177°		172°		147°	
CNN_{Ph}	117°		116°		130°	
CNN_{napht}	116°		113°		127°	
	TDDFT	RASPT2	TDDFT	RASPT2	TDDFT	RASPT2
S_0	0.00	0.00	0.00	0.00	0.00	0.00
S_1	2.56	2.59	2.56	2.56	1.24	1.26
S_2	2.92	3.31	2.59	2.85	2.92	2.75
T_1	-	-	-	-	0.65	0.53
T_2	-	-	-	-	1.72 (SOC_{S1} = 4 cm^{-1})	1.81 (SOC_{S1} = 4 cm^{-1})
Bright transition	$S_0 \rightarrow S_2$ = 2.92 (0.56)	$S_0 \rightarrow S_2$ = 3.31 (0.14)	$S_2 \rightarrow S_1$ = 2.59 (0.58)	$S_2 \rightarrow S_0$ = 2.85 (0.21)	$S_1 \rightarrow S_3$ = 2.10 (0.30)<br>$S_1 \rightarrow S_{10}$ = 3.35 (0.18)	$S_1 \rightarrow S_{13}$ = 3.48 (0.11)
Exp. signal	GSB = 2.47 eV		SE = 2.0<br>ESA1 = 2.14<br>ESA3 > 2.76<br>ESA2 = 2.19			

7. TRANSIENT ABSORPTION MAPS

7.1. 4-DIETHYLAMINO-4'-NITROAZOBENZENE (DNAB)

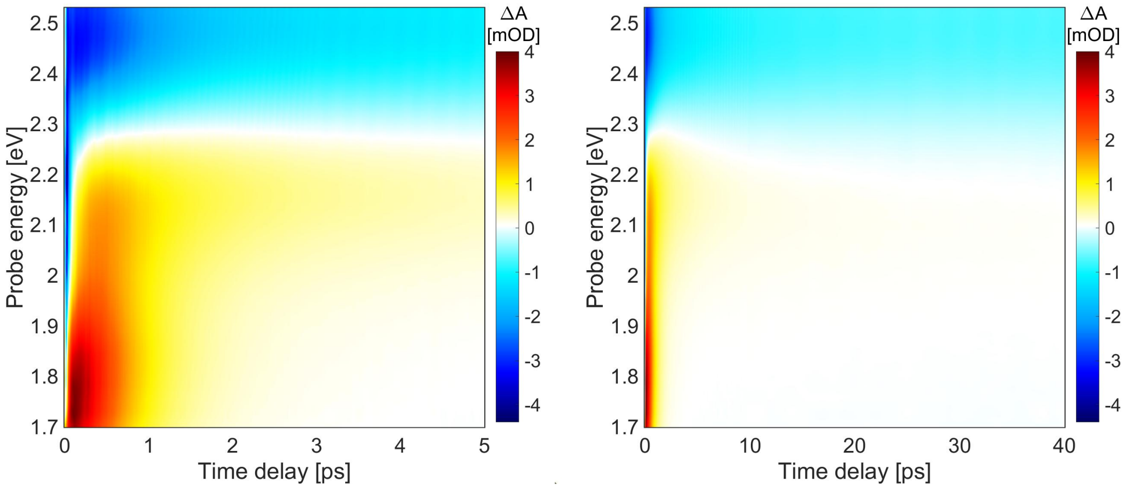

Figure S15. Transient absorption maps of DNAB until 5 ps (left) and 40 ps (right).

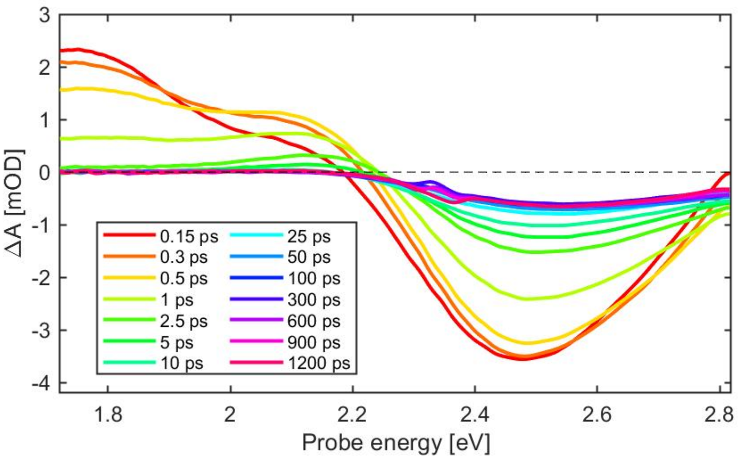

Figure S16. Selected transient absorption spectra of DNAB up to 1.2 ns.

7.2. SUDAN RED G

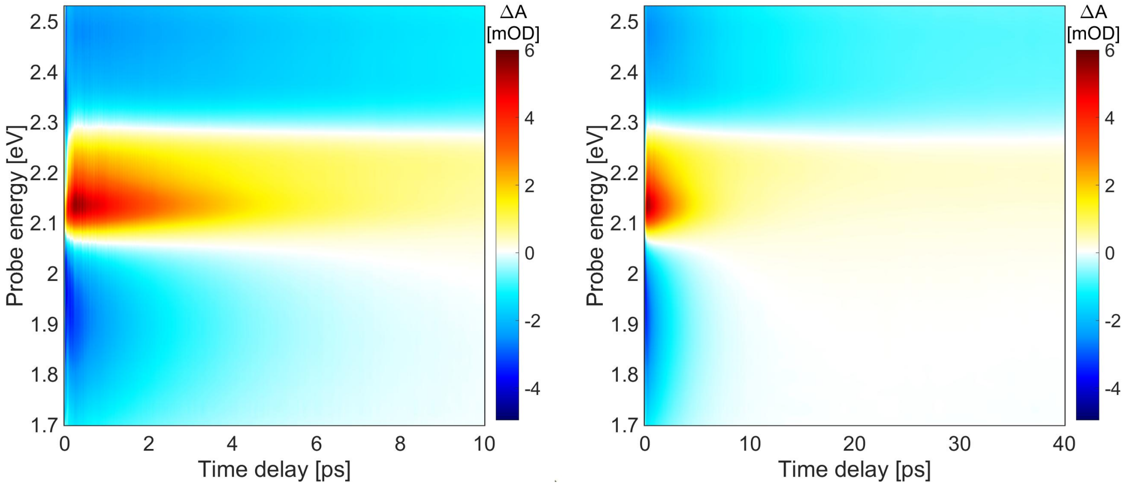

Figure S17. Transient absorption maps of SRG until 10 ps (left) and 40 ps (right).

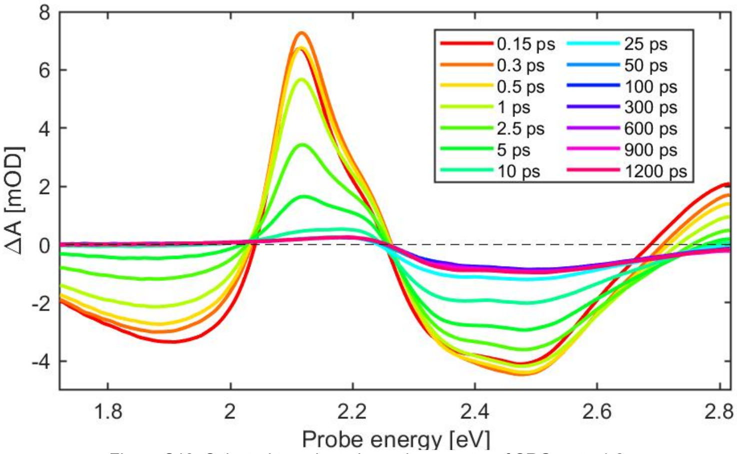

Figure S18. Selected transient absorption spectra of SRG up to 1.2 ns

7.3. DISPERSE BLUE 366

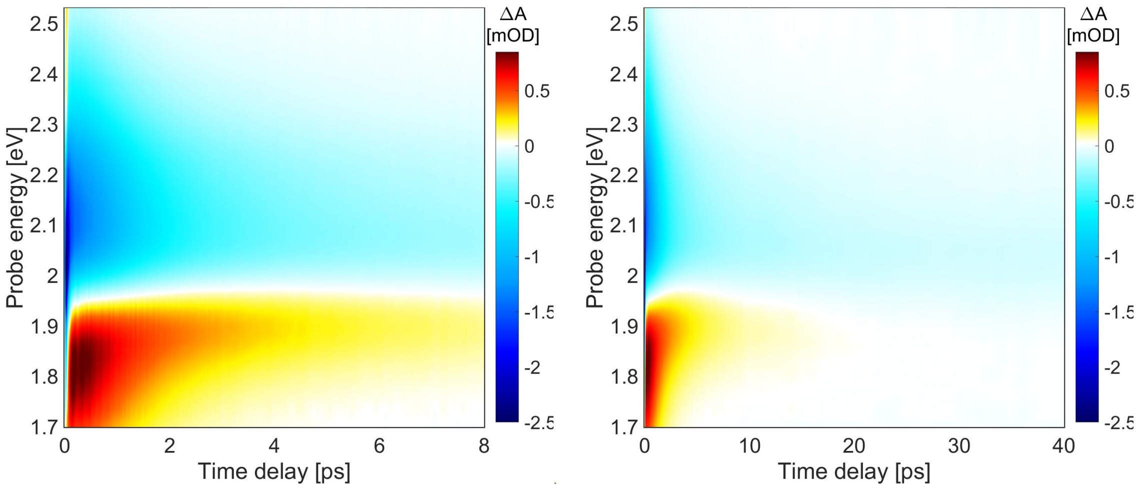

Figure S19. Transient absorption maps of DB366 until 6 ps (left) and 40 ps (right).

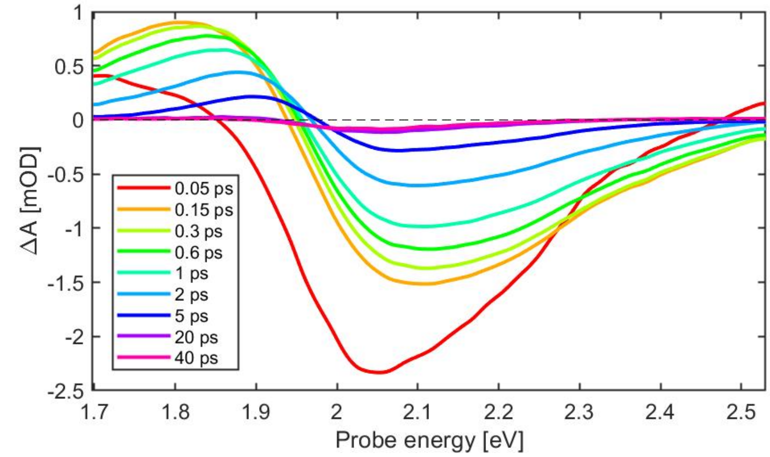

Figure S20. Selected transient absorption spectra of DB366 up to 40 ps.

7.4. DISPERSE BLUE 165

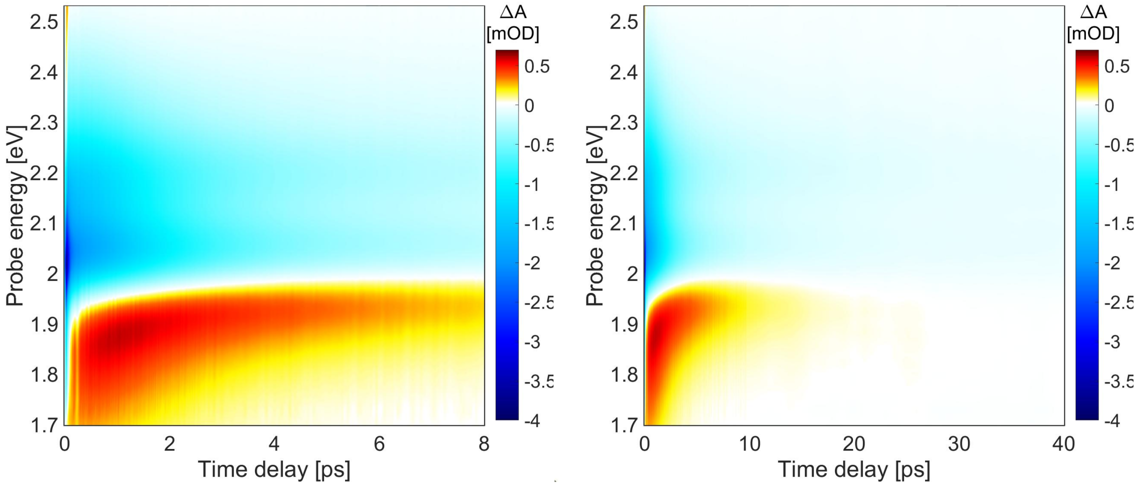

Figure S21. Transient absorption maps of DB165 until 10 ps (left) and 40 ps (right).

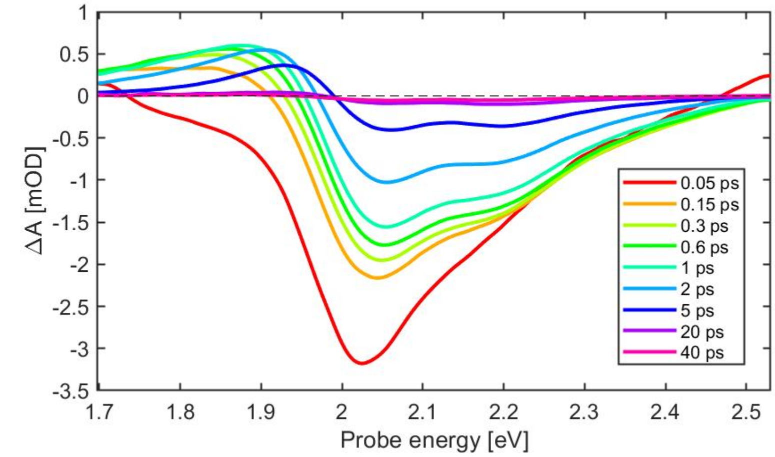

Figure S22. Selected transient absorption spectra of DB165 up to 40 ps.

8. DISPERSE BLUE DYES: BENDING AND TORSIONAL SCANS (PCM)

The bending S_1/S_0 CI reachable through C-N-N bending motions ($CI_{bend\text{-}n\pi^*/GS}$) was characterized through an optimized scan along the symmetric bending coordinate, starting from the S_1 minimum and increasing the C-N-N angles with steps of 5°. Once the CI was found, a total of 5 structures were interpolated between the S_1 minimum and the $CI_{n\pi^*/GS}$.

Concerning the torsional pathway, a relaxed scan was performed in PCM by along the CNNC dihedral by steps of 20° from the planar S_1 structure until 100°. Free optimizations of the nπ* state (without any constraint) were run starting from CNNC values of 120° and 100°: these computations end up in a $CI_{n\pi^*/GS}$ along the torsional path, which is documented in Figures S24 and S26.

8.1 DB366

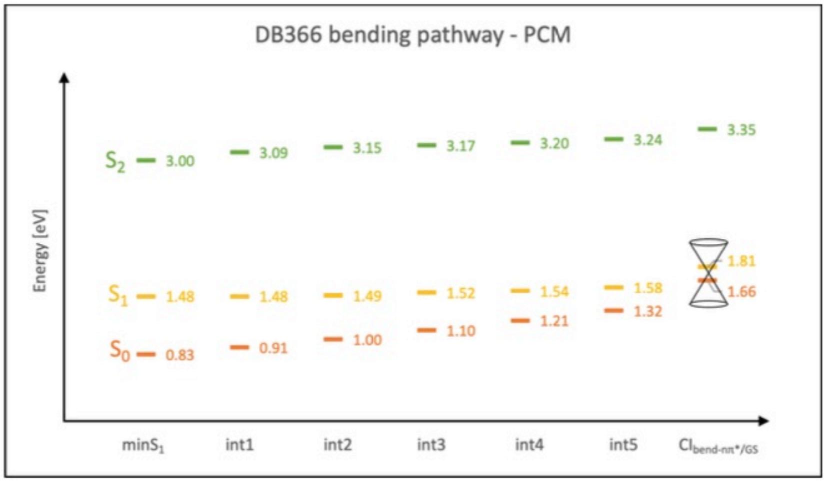

Parameter	$minS_1$	int1	int2	int3	int4	int5	$CI_{bend\text{-}n\pi^*/GS}$
CNNC	180°	179°	179°	178°	177°	177°	176°
CNN_{NO2}	130°	132°	133°	135°	136°	137°	139°
CNN_{NEt2}	135°	136°	137°	138°	139°	140°	140°

Figure S23. Bending pathway for DB366 computed with polarizable continuum model (PCM).

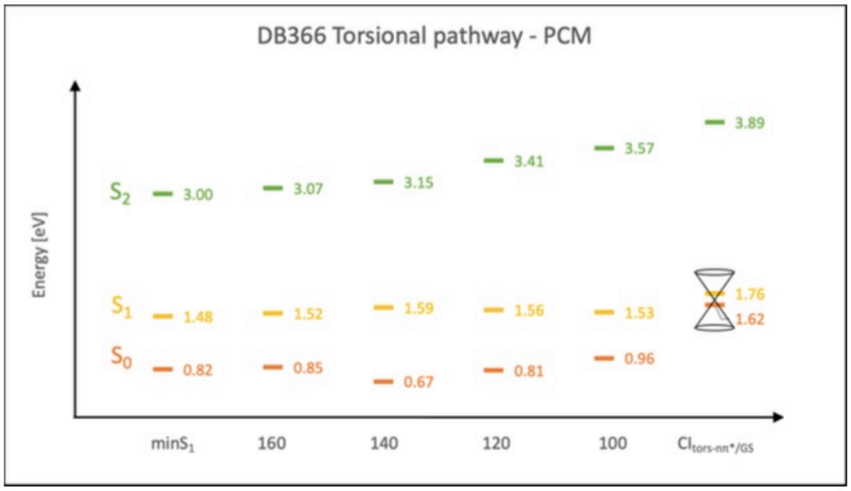

Parameter	$minS_1$	Intermediate structures				$CI_{tors\text{-}n\pi^*/GS}$
CNNC	180°	-160°	-140°	-120°	-100°	-105°
CNN_{NO2}	130°	129°	121°	120°	119°	117°
CNN_{NEt2}	135°	134°	132°	131°	132°	140°

Figure S24. Torsional pathway for DB366 computed with polarizable continuum model (PCM).

8.2 DB165-C1

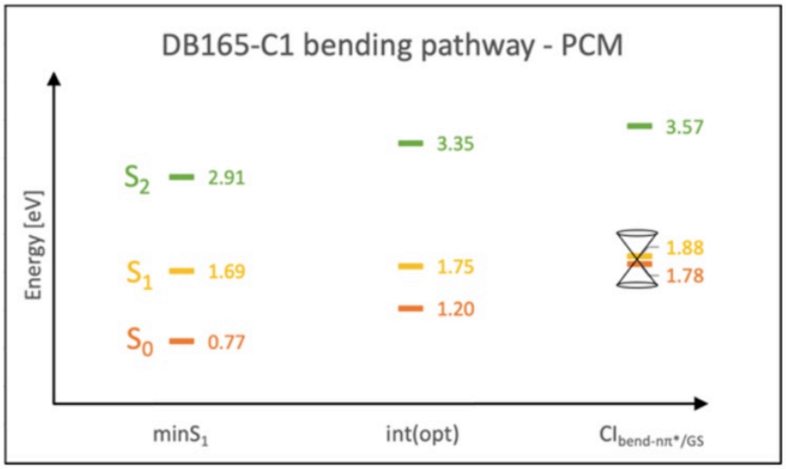

Parameter	$minS_1$	int(opt)	$CI_{bend\text{-}n\pi^*/GS}$
CNNC	179°	174°	-180°
CNN_{NO2}	133°	138°	143°
CNN_{NEt2}	129°	135°	140°

Figure S25. Bending pathway for DB165-C1 computed with polarizable continuum model (PCM).

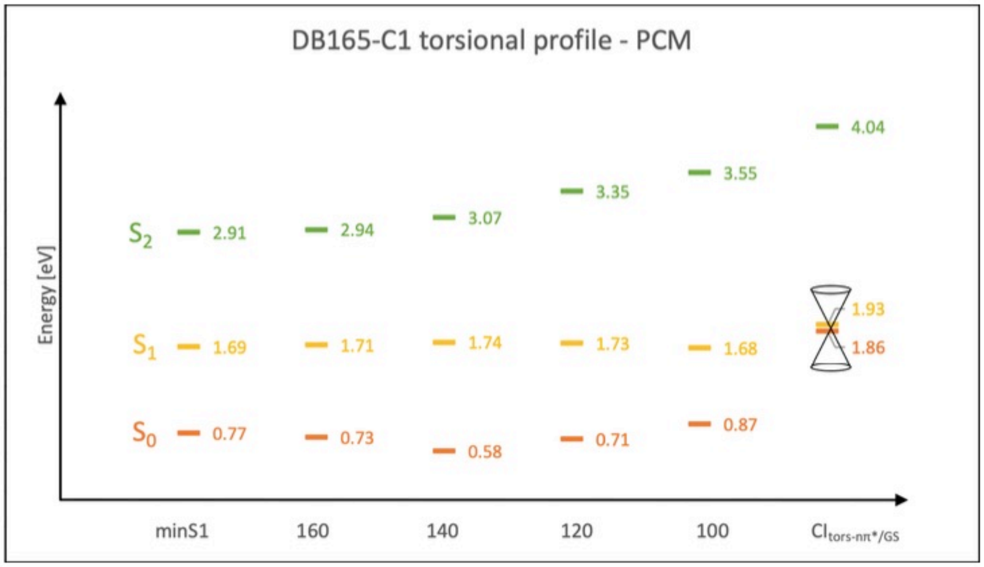

Parameters	$minS_1$	Intermediate structures				$CI_{tors\text{-}n\pi^*/GS}$
CNNC	179°	-160°	-140°	-120°	-100°	-104°
CNN_{NO2}	133°	130°	122°	119°	118°	121°
CNN_{NEt2}	129°	129°	127°	126°	124°	134°

Figure S26. Torsional pathway for DB165-C1 computed with polarizable continuum model (PCM).

8.3 DB165-C2

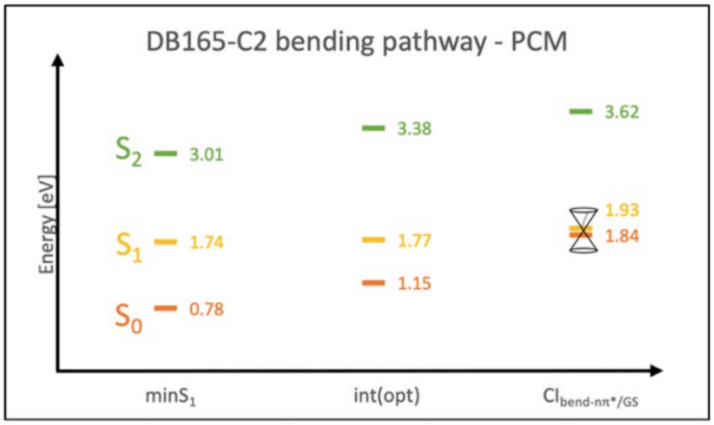

Parameter	$minS_1$	int(opt)	$CI_{bend\text{-}n\pi^*/GS}$
CNNC	174°	178°	174°
CNN_{NO2}	128°	133°	137°
CNN_{NEt2}	137°	141°	147°

Figure S27. Bending pathway for DB165-C2 computed with polarizable continuum model (PCM).

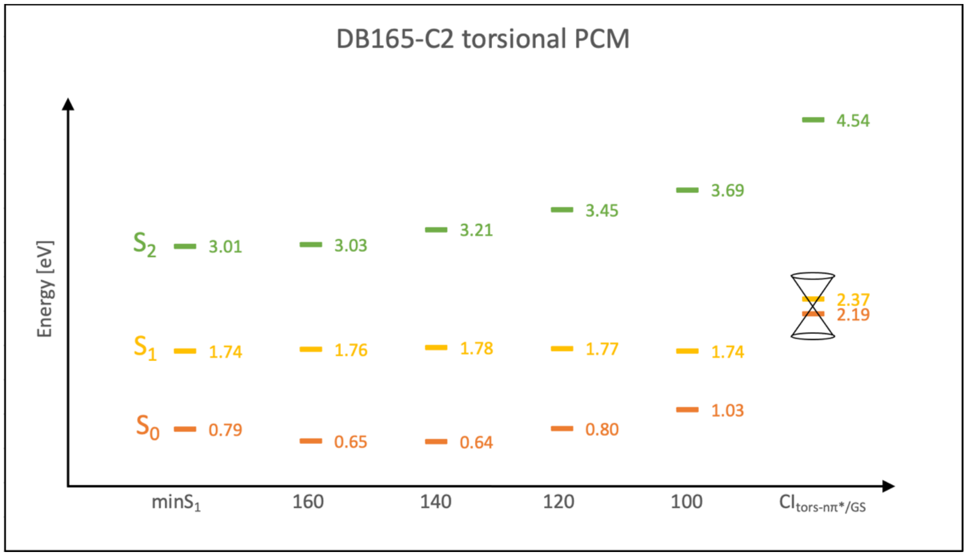

Parameters	minS1	Intermediate structures				$CI_{tors\text{-}n\pi^*/GS}$
CNNC	-179°	-160°	-140°	-120°	-100°	-104°
CNN_{NO2}	129°	124°	120°	119°	118°	120°
CNN_{NEt2}	135°	134°	132°	131°	130°	140°

Figure S28. Torsional pathway for DB165-C2 computed with polarizable continuum model (PCM).

9. SUDAN RED G - NH TAUTOMER: TORSIONAL SCAN

The S_1 potential energy surface of SRG-NH was explored by means of a relaxed scan along the CNNC torsion coordinate. Calculations were conducted at the M06/6-31G* level of theory with implicit solvation (PCM, methanol).

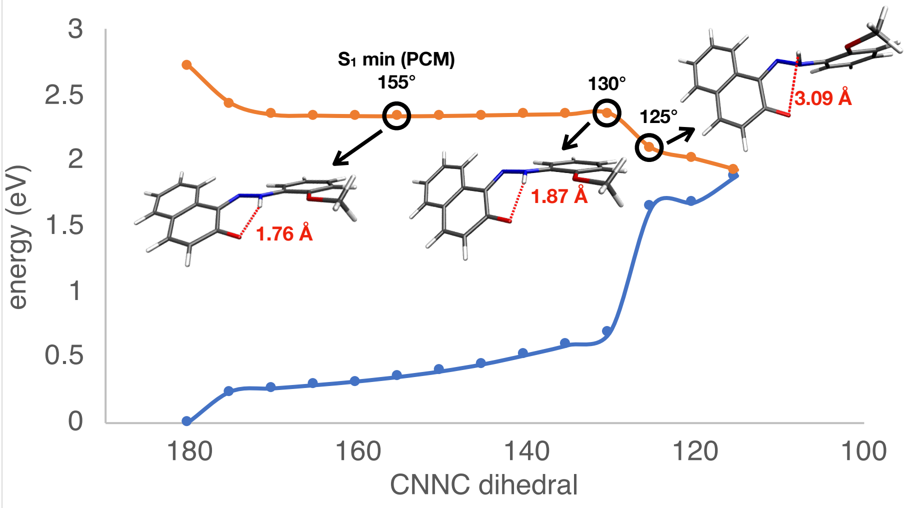

Figure S29. CNNC torsion scan of SRG-NH. The hydrogen bond break beyond 130° causes a significant S_0 destabilization leading to the crossing with S_1.

10. SYNTHESIS AND PURIFICATION OF DISPERSE BLUE DYES

Analytical-grade solvents and commercially available reagents were used as received. The commercially available reagents used were: 2,6-dicyano-4-nitro-anilin (**1**); N-(3-(diethylamino)phenyl)acetamide (**3b**); N,N-diethyl-3-methylaniline (**3a**); sodium nitrite; sodium chloride; sodium sulfate; sodium hydroxide; sulfuric acid; phosphoric acid; sulfamic acid. The following solvents were used: *n*-hexane; petroleum ether; ethyl acetate; demineralized water; acetonitrile. Deuterated solvents for NMR spectra are commercially available.

^{1}H-NMR and ^{13}C-NMR spectra were recorded using a spectrometer Varian Unity-Inova 600 (600MHz), Varian Mercury-Plus 400 (400 MHz).

TLC: Silica gel on aluminum foils, 60 Å (Sigma-Aldrich).

Column chromatography: Silica gel (Merck Grade 9385) 60 Å (230-400 mesh, Sigma-Aldrich).

High Performance Liquid Chromatography (HPLC): Waters 600 HPLC pump and a Waters 2487 UV detector with a wavelength set at 214 nm and column Luna C18 (10 µ, 100 Å, 250x21.20 mm, 20 mL/min).

Disperse blue 366 (**4a**) and disperse blue 165 (**4b**) have been synthetized following two consecutive steps (Scheme 1).

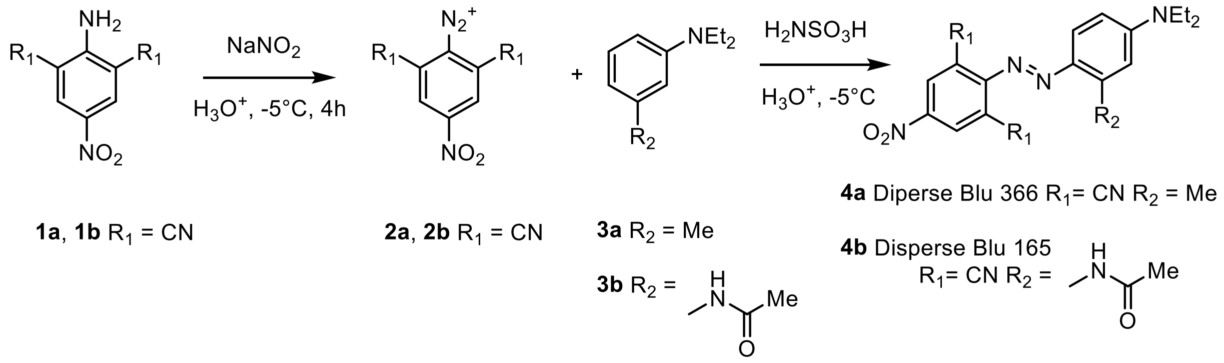

Scheme 1

The first step was the synthesis of diazonium salt **2** from the substituted aniline **1**. The aniline has been reacted with an equivalent of sodium nitrite in sulfuric acid 96% w/w and phosphoric acid 85% w/w at -5 °C for 4 hours. The second step was the slowly addiction of the diazonium salt **2** into a solution of **3a** or **3b** in an acid solution at -5 °C. The process led to the formation of numerous side-products. The crudes were crystallized in acetone and then they were separated with chromatography column on silica gel. In order to obtain high purity of Disperse Blu 366 (**4a**) and Disperse Blue 165 (**4b**), a semi-preparative HPLC separation was necessary after the ordinary steps of purification. A column Luna C18 (10 µ, 100 Å, 250x21.20 mm, 20 mL/min) and acetonitrile/water (90:10) as eluent were used.

DISPERSE BLUE 366 (**4a**): 2,6-dicyano-4-nitro-anilin (**1**, 0.7 g; 3.72 mmol) was dissolved in a stirred solution H_2SO_4 96% w/w (2.45 mL; 46.1 mmol) and H_3PO_4 85% w/w (1.30 mL; 7.14 mmol). The mixture was cooled to -5 °C and then nitrosylsulfuric acid (260 mg of $NaNO_2$ in 1.41 mL of H_2SO_4 96% w/w) was slowly added. The resulting mixture (**2**) was kept under stirring at -5 °C for 4h and was slowly added to a stirred and cooled at -5 °C solution of N,N-diethyl-3-methylaniline **3a** (0.607 g; 3.72 mmol), distilled water (12 mL),

H_2SO_4 96% w/w (0.20 mL; 3.75 mmol) and H_2NSO_3H (37 mg; 0.38 mmol). The reaction took place immediately. The resulting mixture was neutralized (pH = 7) with NaOH to quench the reaction. Then the crude product was transferred in a separating funnel and a brine solution and EtOAc was added. The resulting organic layer was dried over Na_2SO_4 and concentrated in vacuo. The crude solid was firstly purified by precipitation in acetone. Then it was treated over a chromatography column with eluent *n*-hexane/EtOAc 75:25 and finally the pure product was obtained by separation with semi-preparative HPLC (luna-C18, ACN/H_2O 90:10, 20 mL/min, r.t. 6.3 min). The dye **4a** is a blue solid. 15 mg (0.041 mg) of pure product was obtained (yield 1.1%).

^1H NMR (600 MHz, $CDCl_3$) δ 8.61 (s, 2H), 8.07 (d, *J* = 9.5 Hz, 1H), 6.57 (dd, *J* = 9.6, 2.9 Hz, 1H), 6.52 (dd, *J* = 2.9, 1.0 Hz, 1H), 3.50 (q, *J* = 7.2 Hz, 4H), 2.68 (d, *J* = 0.8 Hz, 3H), 1.25 (t, *J* = 7.2 Hz, 6H).

^{13}C NMR (151 MHz, $CDCl_3$) δ 158.5 (Cq), 153.8 (Cq), 143.0 (Cq), 142.3 (Cq), 132.4 (CH), 115.0 (Cq), 111.6 (CH), 110.6 (CH), 106.1 (Cq), 44.6 (CH_2), 18.5 (CH_3), 11.9 (CH_3).

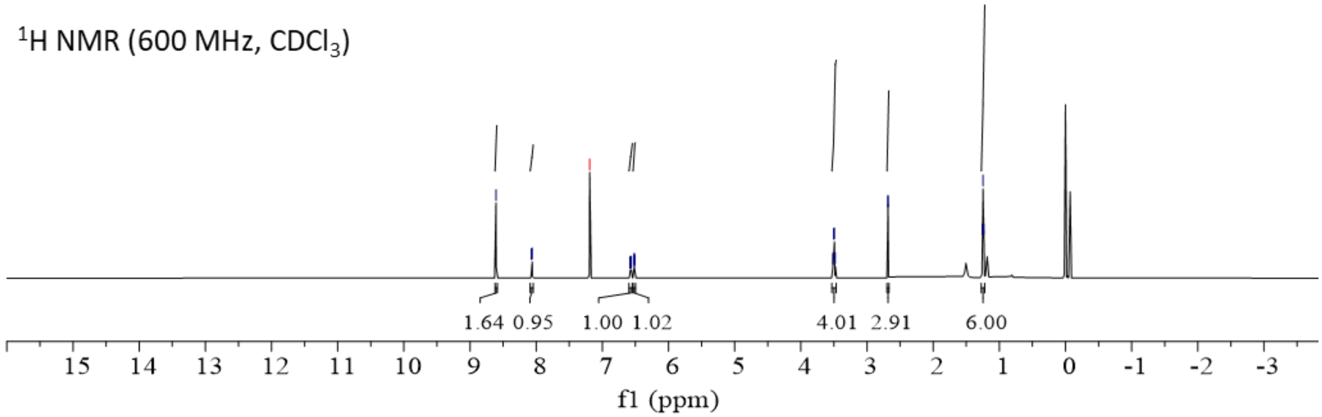

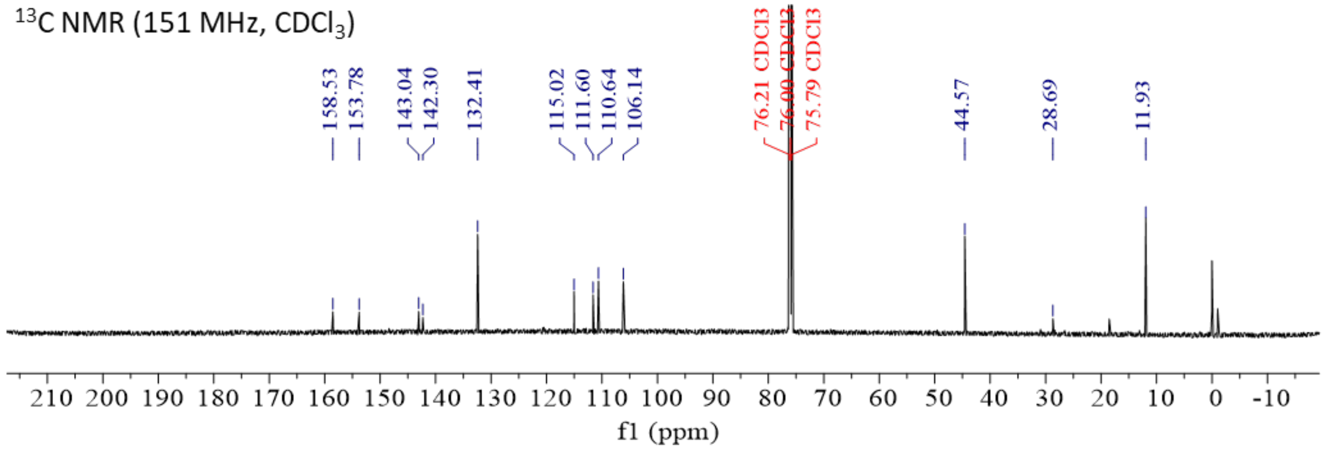

DISPERSE BLUE 165 (**4b**): 2,6-dicyano-4-nitro-anilin (**1**, 1.00 g; 5.32 mmol) was dissolved in a stirred solution H_2SO_4 96% w/w (3.51 mL; 65.8 mmol) and H_3PO_4 85% w/w (1.86 mL; 10.2 mmol). The mixture was cooled to -5 °C and then nitrosylsulfuric acid (372 mg of $NaNO_2$ in 2.02 mL of H_2SO_4 96% w/w) was slowly added. The resulting mixture (**2**) was kept under stirring at -5 °C for 4h and was slowly added to a stirred and cooled at -5 °C solution of N-(3-(diethylamino)phenyl)acetamide **3b** (1.1 g; 5.32 mmol), distilled water (16 mL), H_2SO_4 96% w/w (0.27 mL; 5.06 mmol) and H_2NSO_3H (53 mg; 0.55 mmol). The reaction took place immediately. The resulting mixture was neutralized (pH = 7) with NaOH to quench the reaction. Then the crude product was transferred in a separating funnel and a brine solution and EtOAc was added. The resulting organic layer was dried over Na_2SO_4 and concentrated in vacuo. The crude solid was firstly purified by precipitation in acetone at room temperature. Then it was treated over a chromatography column with a mixture of *n*-hexane/EtOAc 60:40 and finally the pure product was obtained by separation with semi-preparative HPLC (luna-C18, ACN/H_2O 90:10, 20 mL/min, r.t. 5.7 min). The dye **4b** is a blue solid with green reflexes. 10 mg (0.025 mmol) of pure product was obtained (yield 0.5%).

^1H NMR (600 MHz, $CDCl_3$) δ 9.35 (s, 1H), 8.65 (s, 2H), 8.22 (d, *J* = 2.7 Hz, 1H), 8.15 (d, *J* = 9.8 Hz, 1H), 6.58 (dd, *J* = 9.8, 2.8 Hz, 1H), 3.62 (q, *J* = 7.2 Hz, 4H), 2.33 (s, 3H), 1.36 (t, *J* = 7.2 Hz, 6H).

^{13}C NMR (151 MHz, $CDCl_3$) δ 170.8 (Cq), 159.1 (Cq), 157.4 (Cq), 145.3 (Cq), 142.3 (Cq), 135.0 (Cq), 133.7 (CH), 121.3 (CH), 117.5 (Cq), 111.0 (CH), 105.8 (Cq), 100.6 (CH), 46.4 (CH_2), 25.5 (CH_3), 13.0 (CH_3).

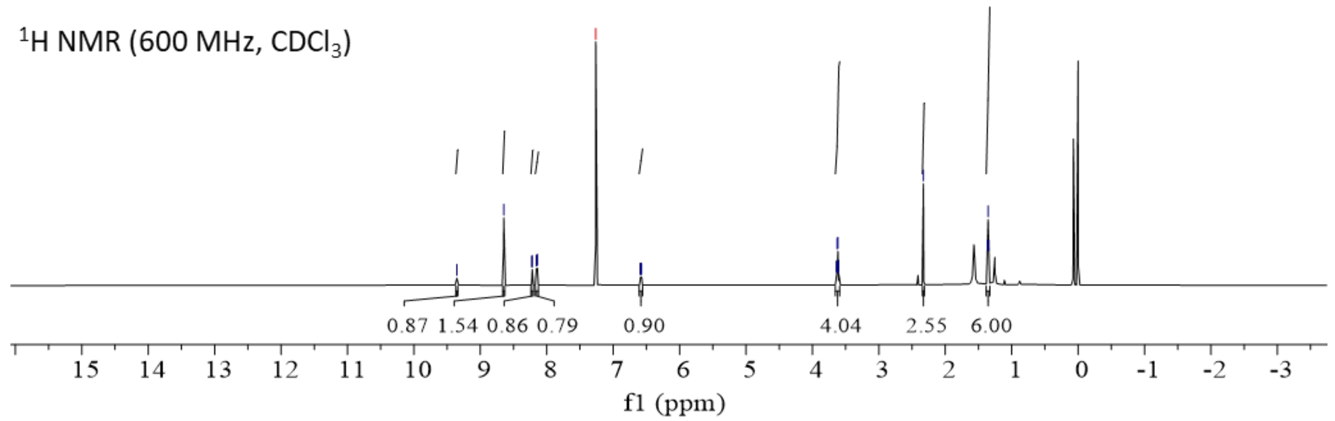

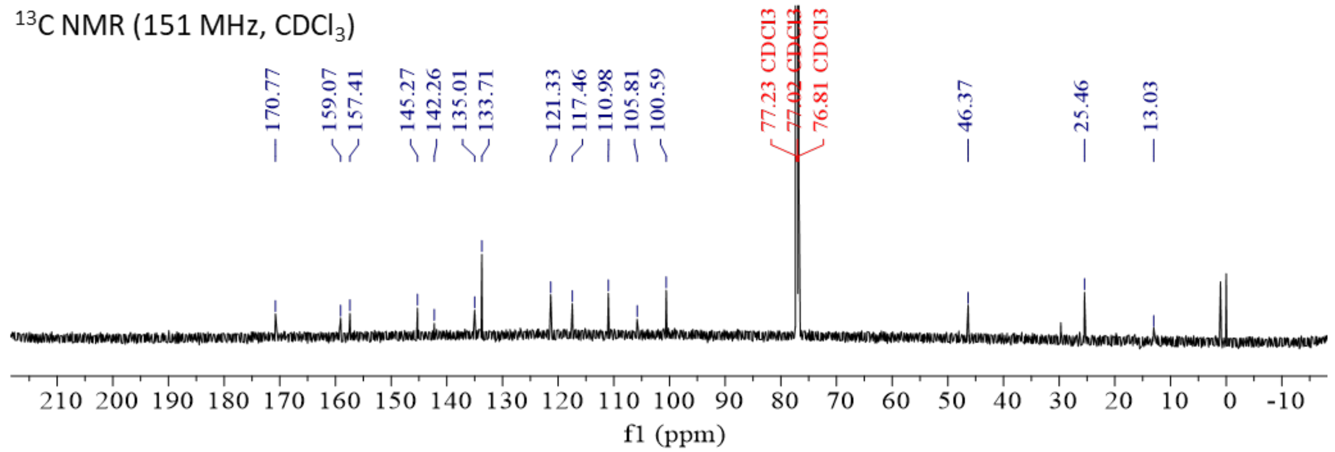

11. FLUORESCENCE QUANTUM YIELD MEASUREMENTS

A solution of Rhodamine B in water was used as a fluorescence quantum yield standard (QY_{REF}=31%).[10] The data of Rhodamine B in water were recorded under the same experimental conditions used for Sudan Red G, while the OD was less than 0.3 OD at the excitation wavelength.

The emission quantum yield of Sudan red G was calculated using the equation:[11]

$$QY = QY_{REF} \frac{n^2 I A_{ref}}{n_{ref}^2 I_{ref} A}$$

Where QY_{REF} is the quantum yield of the reference sample, Rhodamine B in water. A is the absorbance of the solution at the excitation wavelength λ, n is the average refractive index of the solution and I is the integrated area under the emission spectrum. The subscript REF refers to the standard sample.

12. RELATIVE ESTIMATION OF THE PHOTOPRODUCT YIELDS FROM TRANSIENT ABSORPTION MEASUREMENTS

A relative estimation of the photoproduct yields can be made by monitoring the recovery of the ground state bleaching signal (GSB) in the case of the four studied samples. In transient absorption spectroscopy, the pump pulse photoexcites the trans isomer, and the probe pulse monitors its excited state relaxation processes. The area of the GSB signal corresponds to the transitions observed in the linear absorption spectrum of the trans isomers because the pump excitation depletes the ground state population. We calculate the integrated area under the GSB peak, with a bandwidth of ±25 nm, at a pump-probe delay time of 50 fs (right after the coherent artifacts) to estimate the percentage of the excited population. This approach is confirmed by theoretical calculations, which show that the GSB transitions do not overlap with competing transitions of high oscillator strength, ensuring the accuracy of our calculation. The recovery of the GSB signal reflects the excited population returning to the ground state. Therefore, complete recovery of the GSB signal indicates low yields in excited state pathways leading to photoproducts, such as cis isomers and/or triplet states. We monitor the decay of the integrated GSB bandwidth until it reaches a plateau level at 50 ps, which allows us to calculate the relative population that has returned to the ground state after photoexcitation.

For the DNAB and SRG samples, we observe a recovered population of 84% and 79%, respectively, indicating decent isomerization yields. In contrast, for the DB366 (98%) and DB165 (99%) samples, the GSB almost completely recovers, suggesting that these samples block the isomerization pathways to a significant extent.

13. CARTESIAN COORDINATES

DNAB S_0 trans minimum (QM/MM)

C	25.153106	27.671074	25.931667
C	25.056469	26.299217	25.641085
C	23.939509	25.804289	24.998030
C	22.920791	26.685331	24.631627
C	23.009282	28.053316	24.878070
C	24.131379	28.539144	25.523452
H	25.863045	25.635907	25.936020
H	23.847411	24.745290	24.769211
N	21.724522	26.167423	23.995129
H	22.191703	28.706937	24.591903
H	24.230677	29.595989	25.759095
N	26.221804	28.271844	26.623148
N	26.959888	27.434987	27.225737
C	28.016026	27.966341	27.948899
C	28.373025	29.326301	28.010725
C	29.409070	29.745845	28.810924
C	30.124517	28.835418	29.635154
C	29.769233	27.467024	29.549984
C	28.751051	27.055716	28.721779
H	27.813209	30.044369	27.419259
H	29.648550	30.805229	28.829120
N	31.112699	29.259753	30.482427
H	30.270023	26.722810	30.160989
H	28.472464	26.007680	28.695301
O	21.539082	24.951867	24.013146
O	20.955766	26.961630	23.470767
C	31.680441	28.355015	31.479045
C	31.453006	30.672635	30.602197
C	30.738183	28.101119	32.643986
H	32.609314	28.813248	31.840645
H	31.979092	27.412345	31.001399
H	30.508361	29.038004	33.166033
H	29.788401	27.680803	32.291705
H	31.180195	27.398661	33.358795
H	30.543581	31.284228	30.589328
H	31.894694	30.818182	31.595497
C	32.430594	31.131504	29.534001
H	33.377552	30.579327	29.611449
H	32.024602	30.964257	28.527613
H	32.642937	32.201875	29.635252

DNAB S_1 minimum (QM/MM)

C	25.054311	27.785111	25.932484
C	24.847573	26.395901	25.719362
C	23.723516	25.976427	25.054251
C	22.783646	26.912116	24.587472
C	22.993435	28.289667	24.781036
C	24.114491	28.722311	25.443549
H	25.588435	25.680456	26.068648
H	23.546816	24.921951	24.865249
N	21.613265	26.464328	23.926311
H	22.247537	28.993263	24.425610
H	24.269940	29.780540	25.631684
N	26.126485	28.242846	26.627444
N	27.033722	27.662069	27.207670
C	28.079708	28.097316	27.978229
C	28.440122	29.451998	28.106525
C	29.485137	29.812891	28.928513
C	30.194103	28.857438	29.700166
C	29.823871	27.501155	29.541594
C	28.805146	27.134274	28.695439
H	27.895737	30.210712	27.551158
H	29.733725	30.867006	28.998801
N	31.193323	29.223289	30.565075
H	30.313396	26.718992	30.113564
H	28.525267	26.091555	28.628345
O	21.394210	25.237281	23.870483
O	20.854791	27.306554	23.421324
C	31.720501	28.267544	31.536511
C	31.544465	30.625351	30.758298
C	30.755613	27.995079	32.678162
H	32.654660	28.687580	31.928295
H	32.005607	27.336032	31.029353
H	30.534803	28.919744	33.226368
H	29.804697	27.597241	32.305282
H	31.180947	27.268290	33.379530
H	30.637141	31.240332	30.803102
H	32.007869	30.709264	31.748922
C	32.496030	31.143630	29.694738
H	33.432291	30.570078	29.703255
H	32.059827	31.055604	28.691177
H	32.730732	32.200015	29.865255

DNAB S_2 minimum (QM/MM)

C	25.108097	27.657754	25.927265
C	24.954882	26.301725	25.619408
C	23.816483	25.874420	24.955271
C	22.831257	26.802636	24.602955
C	22.992342	28.160142	24.880791
C	24.140222	28.581603	25.541311
H	25.728896	25.594989	25.908139
H	23.662434	24.829798	24.702089
N	21.633780	26.351488	23.949001
H	22.209108	28.854343	24.597434
H	24.286044	29.630997	25.788936
N	26.232630	28.185344	26.642450
N	26.964523	27.339541	27.184245
C	28.028999	27.928417	27.978507
C	28.355259	29.286403	28.017615
C	29.368972	29.736966	28.843785
C	30.063202	28.843412	29.699109
C	29.715854	27.471491	29.642922
C	28.720666	27.035570	28.788470
H	27.808387	29.981169	27.387705
H	29.606701	30.796028	28.837338
N	31.041375	29.279191	30.563505
H	30.204337	26.740586	30.279130
H	28.442813	25.987810	28.778045
O	21.417324	25.112197	23.903895
O	20.869068	27.212122	23.460185
C	31.654580	28.372546	31.530818
C	31.424741	30.683089	30.640618
C	30.747676	28.106382	32.724391
H	32.588150	28.839351	31.865694
H	31.937281	27.435704	31.034192
H	30.544502	29.032241	33.275234
H	29.785027	27.694111	32.400470
H	31.217988	27.388207	33.404077
H	30.534665	31.316116	30.578351
H	31.851082	30.846056	31.637426
C	32.442614	31.066298	29.576499
H	33.361076	30.474580	29.683959
H	32.043172	30.901522	28.568463
H	32.699206	32.126975	29.667731

DNAB S_0 cis minimum (QM/MM)

C	24.749933	22.838110	25.877425
C	24.122609	22.858212	27.121413
C	24.831863	22.494839	28.253282
C	26.138153	22.045179	28.106760
C	26.765427	21.969262	26.867958
C	26.068682	22.390664	25.747420
H	23.090544	23.185724	27.210034
H	24.384433	22.538075	29.240289
N	26.887065	21.665622	29.299748
H	27.785541	21.600802	26.798442
H	26.535016	22.370996	24.761535
N	24.021404	23.091873	24.670918
N	24.102463	24.176636	24.067449
C	24.802411	25.277010	24.673772
C	25.979183	25.761078	24.113246
C	26.614400	26.864065	24.666413
C	26.072549	27.550783	25.775710
C	24.828339	27.095406	26.263244
C	24.226357	25.964946	25.740913
H	26.418204	25.252970	23.255624
H	27.546547	27.196576	24.214403
N	26.734554	28.603431	26.377201
H	24.334541	27.603879	27.086679
H	23.280372	25.620413	26.156055
O	26.517893	22.124219	30.370066
O	27.849225	20.924422	29.155486
C	26.083702	29.416147	27.395620
C	28.076992	28.975516	25.971414
C	25.070127	30.407242	26.843144
H	25.615212	28.762744	28.143339
H	26.875937	29.950755	27.932635
H	25.543242	31.103111	26.137082
H	24.251915	29.898125	26.320242
H	24.624529	30.997709	27.654773
H	28.592011	29.366339	26.860203
H	28.625067	28.065805	25.688761
C	28.133851	30.000778	24.848631
H	27.559360	29.660090	23.976702
H	27.703765	30.957738	25.173767
H	29.170214	30.185184	24.532598

DNAB S_2/S_1 decay (QM/MM 0K dynamics)

C	25.102508	27.586572	25.986019
C	25.016166	26.232644	25.601658
C	23.877268	25.767606	24.925856
C	22.875523	26.690649	24.597327
C	22.954035	28.069185	24.878621
C	24.098074	28.527371	25.590391
H	25.796967	25.521914	25.855023
H	23.705954	24.720697	24.675995
N	21.678238	26.217966	23.944346
H	22.145530	28.752835	24.629554
H	24.136795	29.588237	25.835598
N	26.209163	28.152613	26.700361
N	27.049374	27.326119	27.105509
C	28.069883	27.935663	27.926868
C	28.387938	29.306566	27.997685
C	29.419662	29.756579	28.831265
C	30.089745	28.861490	29.690245
C	29.752478	27.472724	29.656404
C	28.749838	27.037937	28.767863
H	27.892023	30.027011	27.353874
H	29.727115	30.799226	28.782075
N	31.076495	29.283040	30.557442
H	30.215073	26.726833	30.300577
H	28.549354	25.971816	28.796188
O	21.441338	24.996610	23.912712
O	20.912197	27.056740	23.438597
C	31.669474	28.385734	31.535439
C	31.455264	30.700603	30.622447
C	30.753795	28.147984	32.731570
H	32.610402	28.841355	31.867270
H	31.931068	27.436184	31.050431
H	30.558244	29.078162	33.276702
H	29.792745	27.743347	32.393977
H	31.210139	27.427782	33.418368
H	30.573245	31.341167	30.533584
H	31.852913	30.868622	31.631464
C	32.495056	31.048703	29.567550
H	33.399096	30.438058	29.692425
H	32.092820	30.873141	28.561910
H	32.774977	32.105579	29.637936

DNAB S_1/S_0 decay (QM/MM 0K dynamics)

C	25.298959	27.732375	26.288948
C	24.995626	26.329035	26.180406
C	23.960959	25.904131	25.368761
C	23.095362	26.835953	24.736821
C	23.418109	28.217931	24.824424
C	24.474698	28.664250	25.617891
H	25.658351	25.593736	26.645967
H	23.793890	24.828990	25.263974
N	22.009958	26.408275	23.960334
H	22.792608	28.945974	24.283991
H	24.604596	29.749965	25.649697
N	26.359498	28.099334	26.994594
N	27.221655	27.591419	27.648425
C	28.265138	27.957510	28.393108
C	28.670604	29.324629	28.513351
C	29.783749	29.633313	29.314851
C	30.450074	28.599545	30.053403
C	29.992862	27.264856	29.902253
C	28.917788	26.949088	29.116948
H	28.166102	30.121727	27.975590
H	30.093994	30.665549	29.311812
N	31.479464	28.945471	30.911490
H	30.500524	26.439005	30.391644
H	28.670537	25.893414	29.073705
O	21.789205	25.205509	23.901809
O	21.295088	27.209514	23.371848
C	32.093508	27.947226	31.818106
C	31.894186	30.336384	31.114269
C	31.227448	27.716031	33.051144
H	33.070174	28.357506	32.091631
H	32.269635	27.015725	31.274294
H	31.061249	28.634680	33.625683
H	30.246538	27.349385	32.719167
H	31.669776	26.985468	33.736829
H	31.005280	30.984995	31.197933
H	32.414120	30.390169	32.090289
C	32.836103	30.850664	30.035745
H	33.723109	30.217360	29.912523
H	32.347929	30.872938	29.051494
H	33.137793	31.885615	30.235741

DNAB S_0 trans minimum (PCM)

C	25.054202	27.650014	25.989670
C	24.752721	26.277572	25.990034
C	23.705530	25.798827	25.228817
C	22.957721	26.695510	24.464446
C	23.235708	28.059449	24.446083
C	24.286786	28.528274	25.212657
H	25.354590	25.606139	26.595806
H	23.454821	24.742344	25.214082
N	21.855012	26.190007	23.662966
H	22.632457	28.727603	23.839464
H	24.540410	29.585966	25.229698
N	26.093522	28.246447	26.724682
N	26.766529	27.421335	27.417360
C	27.801017	27.959088	28.158427
C	28.156935	29.322493	28.198609
C	29.205758	29.753942	28.971040
C	29.969531	28.844850	29.758311
C	29.603588	27.474803	29.710641
C	28.550496	27.055998	28.930231
H	27.589693	30.030655	27.599098
H	29.460553	30.809951	28.959680
N	31.008969	29.277613	30.522713
H	30.139431	26.740867	30.305289
H	28.269661	26.004212	28.901923
O	21.620109	24.987532	23.693878
O	21.211386	26.989750	22.993541
C	31.875221	28.352019	31.245791
C	31.327182	30.694149	30.674677
C	31.349922	28.016050	32.630951
H	32.863222	28.826081	31.318865
H	32.024404	27.447162	30.644181
H	31.243448	28.924990	33.237011
H	30.366611	27.532182	32.577326
H	32.037306	27.339364	33.151973
H	30.398468	31.276717	30.703037
H	31.783191	30.816123	31.666258
C	32.268612	31.203939	29.597144
H	33.209149	30.638180	29.603860
H	31.824018	31.102322	28.599123
H	32.507364	32.261414	29.760250

DNAB S_1 minimum (PCM)

C	25.005785	27.566164	25.963961
C	24.656692	26.186346	25.928908
C	23.609086	25.773760	25.147987
C	22.885817	26.709243	24.387174
C	23.224001	28.074781	24.415435
C	24.269275	28.501312	25.192428
H	25.231210	25.481549	26.525357
H	23.325599	24.726164	25.106316
N	21.803463	26.269503	23.582500
H	22.648922	28.775540	23.818238
H	24.548155	29.551604	25.229587
N	26.025456	28.014840	26.716339
N	26.815339	27.425707	27.466852
C	27.841877	27.915640	28.213188
C	28.174744	29.287180	28.232652
C	29.224132	29.734356	29.000007
C	29.999474	28.847722	29.792608
C	29.645694	27.474039	29.760622
C	28.598862	27.021131	28.992333
H	27.596685	29.983029	27.627799
H	29.460891	30.794284	28.973132
N	31.040563	29.299390	30.552114
H	30.191621	26.752805	30.361754
H	28.339455	25.964603	28.985114
O	21.518535	25.062847	23.571604
O	21.177839	27.107166	22.916302
C	31.922003	28.389240	31.273413
C	31.339183	30.719968	30.688177
C	31.412723	28.053048	32.664899
H	32.905414	28.874527	31.337215
H	32.078675	27.482371	30.675986
H	31.301025	28.963715	33.267496
H	30.434303	27.558071	32.622539
H	32.111955	27.386556	33.183496
H	30.401854	31.289617	30.716577
H	31.799479	30.859944	31.675611
C	32.266250	31.235181	29.600160
H	33.216446	30.685735	29.608341
H	31.819177	31.114789	28.605285
H	32.487595	32.298468	29.750087

DNAB S_1/S_0 bending CI (PCM)

C	25.025316	27.580537	25.985649
C	24.677411	26.195316	25.953402
C	23.633646	25.786840	25.170859
C	22.906205	26.719808	24.403220
C	23.247878	28.089583	24.434207
C	24.286930	28.520915	25.208981
H	25.247430	25.487493	26.550404
H	23.351994	24.739079	25.129927
N	21.835052	26.281481	23.602771
H	22.673611	28.788730	23.834223
H	24.562369	29.571920	25.242376
N	26.036245	28.013587	26.734204
N	26.881164	27.585626	27.492657
C	27.919040	28.026028	28.248324
C	28.281303	29.387665	28.277478
C	29.337637	29.799608	29.059722
C	30.083880	28.890631	29.848560
C	29.698455	27.528366	29.799403
C	28.646255	27.102226	29.020954
H	27.727659	30.106047	27.676909
H	29.597194	30.854435	29.043794
N	31.130374	29.310487	30.624966
H	30.221776	26.788131	30.398204
H	28.365942	26.051704	29.007982
O	21.546916	25.069928	23.590142
O	21.204539	27.118339	22.929948
C	31.986580	28.370209	31.335212
C	31.451995	30.721967	30.784719
C	31.458917	28.011393	32.714716
H	32.978614	28.834660	31.419904
H	32.133112	27.472835	30.719957
H	31.356165	28.910468	33.336125
H	30.472544	27.534547	32.652418
H	32.141071	27.321615	33.225870
H	30.523394	31.306443	30.819687
H	31.910906	30.839848	31.775924
C	32.389561	31.244078	29.708405
H	33.331147	30.679800	29.707544
H	31.942868	31.149219	28.710559
H	32.627550	32.300928	29.877994

DNAB S_1/S_0 torsion CI (PCM)

C	-0.288744	0.283156	-0.047680
C	-0.225853	-0.313243	1.229286
C	0.997607	-0.654962	1.768523
C	2.157072	-0.402759	1.037570
C	2.124046	0.191073	-0.226134
C	0.903746	0.541430	-0.758885
H	-1.142932	-0.482992	1.788302
H	1.068653	-1.115051	2.749374
N	3.444364	-0.758350	1.616336
H	3.050066	0.374160	-0.762005
H	0.830818	1.010271	-1.737495
N	-1.457494	0.661218	-0.657282
N	-2.583764	0.377712	-0.106587
C	-3.426102	-0.627373	-0.403396
C	-3.134525	-1.627237	-1.366134
C	-4.033138	-2.633732	-1.631993
C	-5.276502	-2.722960	-0.953947
C	-5.556282	-1.720552	0.014448
C	-4.667630	-0.707718	0.274002
H	-2.193649	-1.580141	-1.911221
H	-3.773270	-3.361673	-2.395774
N	-6.168904	-3.722055	-1.233898
H	-6.488068	-1.742774	0.571904
H	-4.901195	0.052508	1.017017
O	3.463513	-1.257292	2.737246
O	4.460624	-0.482712	0.985257
C	-7.455031	-3.821616	-0.555730
C	-5.821046	-4.827141	-2.120186
C	-7.364314	-4.522095	0.789950
H	-8.129161	-4.371845	-1.225642
H	-7.894757	-2.821331	-0.456740
H	-6.982773	-5.544427	0.670626
H	-6.689714	-3.989682	1.472015
H	-8.351777	-4.581763	1.262510
H	-4.772629	-5.112347	-1.962178
H	-6.414668	-5.694726	-1.803212
C	-6.103746	-4.517737	-3.580695
H	-7.170655	-4.304114	-3.726255
H	-5.537978	-3.642528	-3.923566
H	-5.837850	-5.370532	-4.216326

DNAB S_0 cis minimum (PCM)

C	24.719416	27.607009	26.100960
C	23.885477	26.532034	26.448705
C	22.764261	26.255390	25.691812
C	22.480761	27.056552	24.586468
C	23.281739	28.142280	24.240543
C	24.391347	28.428377	25.015059
H	24.130926	25.920208	27.314251
H	22.108360	25.426237	25.939364
N	21.312454	26.756728	23.778404
H	23.024383	28.750140	23.378601
H	25.028972	29.278196	24.781529
N	25.771203	28.000359	26.959431
N	26.723434	27.255416	27.300405
C	27.035235	26.032883	26.690386
C	26.763800	25.650431	25.362666
C	27.198973	24.440054	24.873540
C	27.909552	23.520012	25.689398
C	28.209372	23.929566	27.013236
C	27.807493	25.162030	27.476290
H	26.237199	26.320518	24.687778
H	26.974958	24.194414	23.839425
N	28.304498	22.306596	25.211255
H	28.793214	23.290147	27.669061
H	28.068626	25.480379	28.484379
O	20.616137	25.800285	24.099231
O	21.077301	27.472527	22.811547
C	28.932383	21.307088	26.068148
C	28.114152	21.922552	23.816760
C	30.443426	21.449010	26.132402
H	28.660730	20.321401	25.666594
H	28.484506	21.352888	27.068573
H	30.884543	21.362196	25.131033
H	30.734749	22.424109	26.542631
H	30.879896	20.666735	26.764527
H	28.285688	22.792262	23.170368
H	28.911044	21.208902	23.568086
C	26.752774	21.300761	23.556679
H	26.609188	20.407413	24.178133
H	25.942907	22.003820	23.788893
H	26.656930	21.002697	22.505957

SRG-NH S_0 trans minimum (QM/MM)

N	26.331528	26.161064	26.223897
N	25.357374	25.358212	25.906097
C	25.305420	24.061000	26.425791
C	26.410622	27.374017	25.706399
C	24.194244	23.271422	26.072729
C	24.105430	21.964004	26.537301
C	25.109465	21.447563	27.354748
C	26.195023	22.235361	27.718489
C	26.296227	23.540029	27.251600
O	23.278695	23.884287	25.282124
H	23.263443	21.339267	26.252087
H	25.040159	20.418460	27.700962
H	26.970662	21.834321	28.365420
H	27.139268	24.174230	27.515131
C	27.546163	28.189841	26.130129
C	27.680401	29.500487	25.619968
C	26.709407	29.991805	24.676428
C	25.653266	29.257795	24.264245
C	25.429596	27.908539	24.751608
H	26.861473	31.000017	24.286437
H	24.924320	29.635051	23.548608
O	24.444289	27.245431	24.378116
C	28.760965	30.299487	26.023654
C	29.708734	29.815602	26.905737
C	29.585490	28.510902	27.396344
C	28.520505	27.712148	27.016970
H	28.850638	31.307923	25.617184
H	30.544655	30.440864	27.207280
H	30.335219	28.114851	28.079743
H	28.436418	26.692635	27.384752
C	22.121303	23.163017	24.930956
H	21.535072	23.819708	24.284995
H	22.368731	22.238740	24.393267
H	21.525062	22.915129	25.819931
H	24.650563	25.685629	25.231384

SRG-NH S_1 minimum (QM/MM)

N	26.219318	26.383861	26.674398
N	25.147408	25.591599	26.477832
C	25.314842	24.229838	26.594989
C	26.383196	27.453120	25.922593
C	24.229095	23.396041	26.215746
C	24.325851	22.020347	26.375076
C	25.483454	21.464163	26.915998
C	26.549580	22.280650	27.290828
C	26.480907	23.652073	27.116958
O	23.170007	24.054653	25.696452
H	23.513381	21.371423	26.064396
H	25.547088	20.386152	27.046443
H	27.434379	21.838338	27.731678
H	27.297092	24.312010	27.409029
C	27.436521	28.389661	26.266956
C	27.543664	29.621876	25.572310
C	26.621178	29.920528	24.525116
C	25.629781	29.050136	24.171502
C	25.478649	27.772502	24.791698
H	26.738525	30.861975	23.989348
H	24.942790	29.268503	23.357293
O	24.574373	26.963799	24.428536
C	28.566474	30.516724	25.931999
C	29.471539	30.205883	26.928692
C	29.375057	28.978761	27.598788
C	28.367379	28.087939	27.274340
H	28.638640	31.465427	25.395636
H	30.264391	30.904459	27.183303
H	30.091548	28.724504	28.377286
H	28.295264	27.122742	27.771548
C	22.049446	23.295107	25.295376
H	21.331737	24.006162	24.882273
H	22.323618	22.556042	24.529300
H	21.594923	22.777016	26.153316
H	24.467527	25.946886	25.764923

SRG-NH S_1/S_0 decay (QM/MM 0K dynamics)

N	25.677359	26.947377	27.684885
N	24.838386	25.898079	27.349157
C	25.272939	24.596224	27.211728
C	26.088187	27.677180	26.639063
C	24.243598	23.700926	26.727027
C	24.636969	22.441214	26.366992
C	25.967720	22.021340	26.568979
C	26.922557	22.881876	27.064380
C	26.598399	24.209447	27.356914
O	23.036270	24.261630	26.559530
H	23.917367	21.764431	25.922507
H	26.228558	20.998915	26.331982
H	27.944541	22.552240	27.213280
H	27.339542	24.942202	27.669396
C	27.180158	28.637297	26.838512
C	27.349465	29.693063	25.912363
C	26.512504	29.769805	24.742181
C	25.718638	28.728805	24.417279
C	25.429194	27.620732	25.287814
H	26.590983	30.603060	24.060367
H	25.114443	28.674005	23.513993
O	24.537818	26.815058	25.014110
C	28.418366	30.593575	26.092931
C	29.305939	30.423254	27.142429
C	29.223232	29.282945	27.958285
C	28.155120	28.434430	27.801068
H	28.547362	31.415450	25.387761
H	30.126098	31.121592	27.303064
H	29.967132	29.038575	28.713320
H	28.058034	27.548926	28.427165
C	21.973755	23.507480	26.017037
H	21.131067	24.203443	25.994322
H	22.219646	23.157874	25.004487
H	21.751197	22.635541	26.655766
H	23.814196	25.988012	27.395329

SRG-NH T_1 minimum (QM/MM)

N	26.690428	26.270479	26.545581
N	25.624584	25.516903	26.216668
C	25.586763	24.206139	26.555488
C	26.701501	27.525498	26.034983
C	24.408000	23.463036	26.247897
C	24.352121	22.108896	26.541373
C	25.436582	21.487341	27.160960
C	26.581071	22.219201	27.496545
C	26.663315	23.563486	27.199352
O	23.418567	24.184624	25.691835
H	23.474620	21.526225	26.276868
H	25.390039	20.423136	27.378598
H	27.410075	21.720885	27.992033
H	27.538744	24.158767	27.440832
C	27.816294	28.337771	26.435936
C	27.938944	29.679644	25.971608
C	26.955944	30.210933	25.075865
C	25.898424	29.457804	24.683846
C	25.692259	28.103101	25.134497
H	27.088823	31.231691	24.714293
H	25.147079	29.837431	23.993881
O	24.671143	27.460494	24.764284
C	29.031219	30.449483	26.393873
C	29.990896	29.928632	27.242596
C	29.881412	28.603775	27.692222
C	28.817299	27.823990	27.292740
H	29.117556	31.475042	26.037427
H	30.832418	30.544855	27.550282
H	30.637487	28.192586	28.357686
H	28.728225	26.792051	27.623435
C	22.192205	23.536846	25.416644
H	21.524032	24.307286	25.029080
H	22.330499	22.745218	24.669664
H	21.759789	23.110893	26.331920
H	24.883715	25.952234	25.634910

SRG-NH S_0 trans minimum (PCM)

N	26.331528	26.161064	26.223897
N	25.357374	25.358212	25.906097
C	25.305420	24.061000	26.425791
C	26.410622	27.374017	25.706399
C	24.194244	23.271422	26.072729
C	24.105430	21.964004	26.537301
C	25.109465	21.447563	27.354748
C	26.195023	22.235361	27.718489
C	26.296227	23.540029	27.251600
O	23.278695	23.884287	25.282124
H	23.263443	21.339267	26.252087
H	25.040159	20.418460	27.700962
H	26.970662	21.834321	28.365420
H	27.139268	24.174230	27.515131
C	27.546163	28.189841	26.130129
C	27.680401	29.500487	25.619968
C	26.709407	29.991805	24.676428
C	25.653266	29.257795	24.264245
C	25.429596	27.908539	24.751608
H	26.861473	31.000017	24.286437
H	24.924320	29.635051	23.548608
O	24.444289	27.245431	24.378116
C	28.760965	30.299487	26.023654
C	29.708734	29.815602	26.905737
C	29.585490	28.510902	27.396344
C	28.520505	27.712148	27.016970
H	28.850638	31.307923	25.617184
H	30.544655	30.440864	27.207280
H	30.335219	28.114851	28.079743
H	28.436418	26.692635	27.384752
C	22.121303	23.163017	24.930956
H	21.535072	23.819708	24.284995
H	22.368731	22.238740	24.393267
H	21.525062	22.915129	25.819931
H	24.650563	25.685629	25.231384

SRG-NH S_1 minimum (PCM)

N	26.401540	26.146850	26.440472
N	25.310768	25.401331	26.204673
C	25.317949	24.064807	26.454273
C	26.532142	27.286127	25.757863
C	24.128009	23.330765	26.162557
C	24.078491	21.966593	26.417416
C	25.188819	21.324733	26.963218
C	26.356936	22.038979	27.254274
C	26.429082	23.395105	26.999177
O	23.134835	24.063664	25.632814
H	23.179501	21.398406	26.195086
H	25.137864	20.257722	27.168148
H	27.212060	21.525693	27.688161
H	27.321686	23.973708	27.220169
C	27.541879	28.214683	26.200038
C	27.733768	29.445470	25.511045
C	26.893478	29.766136	24.402336
C	25.907443	28.916776	23.989944
C	25.682272	27.641112	24.601745
H	27.051314	30.715011	23.889671
H	25.266326	29.158992	23.144035
O	24.777781	26.859903	24.185145
C	28.734715	30.326325	25.951479
C	29.528094	30.018726	27.042302
C	29.333801	28.810546	27.729082
C	28.358095	27.926249	27.314075
H	28.874149	31.266231	25.417111
H	30.299574	30.713731	27.368092
H	29.953975	28.568440	28.589925
H	28.209267	26.983344	27.835666
C	21.918234	23.411775	25.304524
H	21.260626	24.183262	24.901152
H	22.084321	22.635001	24.547228
H	21.461181	22.966303	26.197330
H	24.589856	25.806339	25.580282

SRG-NH S_0 cis minimum (PCM)

N	26.549322	25.937363	26.138804
N	25.537100	25.104483	26.051029
C	25.350912	23.788678	26.512470
C	27.824933	25.616725	26.079849
C	24.135924	23.179695	26.136301
C	23.806400	21.925238	26.636615
C	24.676067	21.279678	27.514629
C	25.865009	21.885283	27.900896
C	26.203283	23.136848	27.397562
O	23.360418	23.910642	25.298625
H	22.875857	21.445085	26.346032
H	24.412499	20.295210	27.895585
H	26.538400	21.386555	28.594202
H	27.120937	23.623336	27.717789
C	28.752891	26.710068	26.398777
C	30.145027	26.499280	26.281562
C	30.634662	25.222373	25.824471
C	29.807024	24.219107	25.466361
C	28.358295	24.353555	25.518281
H	31.715720	25.091110	25.759734
H	30.183358	23.267071	25.096536
O	27.616575	23.487834	25.041838
C	31.038815	27.528836	26.609616
C	30.575216	28.758406	27.042422
C	29.198311	28.967933	27.162394
C	28.302829	27.955466	26.858067
H	32.108692	27.343192	26.514052
H	31.275796	29.552545	27.292175
H	28.823199	29.930498	27.505702
H	27.233482	28.115911	26.972620
C	22.083298	23.401116	24.965856
H	21.610920	24.152916	24.330528
H	22.166524	22.457099	24.410881
H	21.471756	23.243614	25.864564
H	24.658609	25.604452	25.936953

SRG-NH S_1/S_0 torsion CI (PCM)

N	26.088878	26.376042	26.158539
N	25.156731	25.482929	25.926378
C	25.233125	24.189871	26.374459
C	27.086135	26.520443	25.231498
C	24.125843	23.337108	26.122941
C	24.181187	22.015902	26.546858
C	25.319113	21.559383	27.200966
C	26.411450	22.398986	27.445563
C	26.369387	23.713621	27.033890
O	23.107850	23.905482	25.476509
H	23.349921	21.341337	26.367981
H	25.353000	20.524199	27.528979
H	27.287872	22.019798	27.962014
H	27.191649	24.398136	27.218909
C	28.233117	27.315692	25.543236
C	29.278668	27.448068	24.585678
C	29.159857	26.776325	23.337994
C	28.057011	26.028448	23.048159
C	26.951082	25.883513	23.957536
H	29.968502	26.878833	22.614068
H	27.951180	25.523897	22.088737
O	25.890821	25.237190	23.654631
C	30.408005	28.230549	24.907831
C	30.511807	28.861830	26.126034
C	29.480687	28.724950	27.077289
C	28.368125	27.966239	26.793040
H	31.203682	28.324285	24.167790
H	31.390308	29.461695	26.357544
H	29.565345	29.220479	28.043278
H	27.571443	27.856011	27.526833
C	21.952951	23.120062	25.202463
H	21.258360	23.778492	24.679888
H	22.207129	22.267958	24.561131
H	21.495273	22.763835	26.133076
H	24.275699	25.778373	25.502484

SRG-OH S_0 trans minimum (QM/MM)

N	26.407028	26.084136	26.250288
N	25.457208	25.407175	25.750611
C	25.220670	24.151719	26.322110
C	26.598901	27.346877	25.713132
C	24.120438	23.429086	25.802279
C	23.776717	22.201926	26.371751
C	24.523025	21.690100	27.425336
C	25.626095	22.384041	27.919103
C	25.971186	23.613925	27.375797
O	23.457218	23.990693	24.769785
H	22.924961	21.643547	26.001237
H	24.236802	20.737622	27.870485
H	26.206316	21.970773	28.741593
H	26.822985	24.179240	27.735137
C	27.680787	28.117415	26.270969
C	27.919434	29.433973	25.787427
C	27.088138	29.954638	24.761774
C	26.071272	29.217845	24.226622
C	25.804778	27.906826	24.691524
H	27.281956	30.965851	24.405744
H	25.444372	29.600200	23.422036
O	24.786466	27.271710	24.122128
C	28.986362	30.196448	26.315446
C	29.807978	29.672244	27.289264
C	29.568957	28.371356	27.764203
C	28.532894	27.611917	27.278143
H	29.132503	31.207425	25.934063
H	30.635933	30.260903	27.683989
H	30.203808	27.966870	28.542830
H	28.358686	26.610637	27.670061
H	24.724837	26.367043	24.543258
C	22.287927	23.363090	24.299377
H	21.908231	24.001708	23.497962
H	22.499227	22.361252	23.901643
H	21.533850	23.285951	25.095154

SRG-OH S_2 minimum (QM/MM)

N	26.540470	26.018935	26.283831
N	25.532960	25.364583	25.726437
C	25.244793	24.148905	26.244619
C	26.715204	27.266008	25.756909
C	24.141097	23.441776	25.663925
C	23.714717	22.237282	26.210201
C	24.388182	21.694369	27.303566
C	25.503926	22.341320	27.849526
C	25.929811	23.551388	27.330180
O	23.588724	24.035163	24.590342
H	22.856116	21.716626	25.794235
H	24.035024	20.762859	27.738554
H	26.031160	21.893446	28.689957
H	26.787737	24.082504	27.733286
C	27.748288	28.077529	26.330835
C	27.962295	29.411464	25.871647
C	27.147641	29.934784	24.833404
C	26.177184	29.155545	24.254481
C	25.945212	27.832876	24.673266
H	27.310057	30.959505	24.502986
H	25.552701	29.527403	23.443929
O	25.008176	27.150079	24.032013
C	28.990191	30.174242	26.449153
C	29.808896	29.646010	27.437573
C	29.607931	28.336685	27.888965
C	28.586881	27.569970	27.351395
H	29.136232	31.195168	26.095312
H	30.607219	30.253270	27.860973
H	30.241244	27.918882	28.667577
H	28.413777	26.554778	27.700411
H	24.958499	26.249347	24.504309
C	22.454391	23.439079	23.993724
H	22.175241	24.091331	23.161935
H	22.681583	22.432070	23.616962
H	21.621343	23.375011	24.707706

SRG-OH S_1 minimum (QM/MM)

N	26.798271	25.979274	26.042381
N	25.977245	25.097825	25.668536
C	25.488448	24.024055	26.350016
C	26.824887	27.284357	25.673412
C	24.403470	23.328292	25.757244
C	23.911248	22.182893	26.361213
C	24.487119	21.716344	27.545062
C	25.543511	22.402686	28.139153
C	26.039258	23.561081	27.555267
O	23.919104	23.893451	24.624571
H	23.076591	21.644439	25.920548
H	24.097394	20.813823	28.006362
H	25.989475	22.028536	29.057756
H	26.875494	24.106904	27.985726
C	27.845038	28.139836	26.219176
C	27.951383	29.478005	25.749052
C	27.054196	29.938619	24.751658
C	26.080619	29.114178	24.247752
C	25.932859	27.790418	24.698576
H	27.153712	30.960601	24.390834
H	25.385588	29.453440	23.481555
O	24.952307	27.063648	24.135059
C	28.964304	30.310983	26.276654
C	29.840440	29.840437	27.225885
C	29.731200	28.516332	27.693465
C	28.750103	27.685187	27.200325
H	29.032433	31.334549	25.906577
H	30.621987	30.488629	27.618119
H	30.419103	28.143359	28.449285
H	28.664416	26.663721	27.568267
H	24.963496	26.140326	24.467325
C	22.798712	23.286938	24.010008
H	22.535405	23.920628	23.160221
H	23.034262	22.272714	23.659075
H	21.947370	23.239134	24.703453

SRG-OH S_0 trans minimum (PCM)

N	26.366308	26.117449	26.303049
N	25.391659	25.453705	25.834269
C	25.166877	24.190152	26.390912
C	26.571745	27.367968	25.744448
C	24.085007	23.460325	25.846307
C	23.792981	22.191443	26.348734
C	24.564380	21.658115	27.375893
C	25.632074	22.373389	27.916906
C	25.927719	23.633524	27.423047
O	23.395843	24.059745	24.853463
H	22.966386	21.614031	25.944376
H	24.323675	20.667496	27.756994
H	26.228307	21.946980	28.720534
H	26.752891	24.218392	27.823709
C	27.676931	28.124121	26.271486
C	27.947357	29.419523	25.751195
C	27.117478	29.934199	24.719996
C	26.070178	29.213626	24.220024
C	25.779077	27.922828	24.720378
H	27.331702	30.927713	24.326584
H	25.431038	29.602139	23.429874
O	24.741150	27.289471	24.177824
C	29.029948	30.168861	26.264209
C	29.827115	29.660291	27.260982
C	29.560621	28.376751	27.776059
C	28.512324	27.625149	27.295468
H	29.217834	31.159719	25.850424
H	30.659411	30.242271	27.651620
H	30.192318	27.972634	28.565428
H	28.313581	26.635184	27.698473
H	24.670232	26.402234	24.636072
C	22.305592	23.360803	24.283756
H	21.895882	24.015700	23.512173
H	22.632284	22.417997	23.824558
H	21.530040	23.152236	25.033036

SRG-OH S_1/S_0 bending CI (PCM)

N	26.352167	26.045262	26.338682
N	25.424301	25.434125	25.875846
C	24.663002	24.350631	25.849915
C	27.106340	27.122032	26.366243
C	23.611280	24.349206	24.893320
C	22.776853	23.250153	24.810765
C	22.966432	22.151256	25.661210
C	23.996896	22.153954	26.597724
C	24.850169	23.243189	26.702821
O	23.543386	25.471958	24.138735
H	21.968200	23.231978	24.084973
H	22.299118	21.296087	25.580826
H	24.140028	21.299587	27.255296
H	25.663260	23.270767	27.426415
C	28.204487	27.203098	27.287361
C	28.994456	28.386162	27.287703
C	28.684288	29.442311	26.393994
C	27.621867	29.337084	25.517930
C	26.824935	28.192871	25.484918
H	29.298854	30.341067	26.407888
H	27.374601	30.139794	24.826671
O	25.794943	28.153724	24.594650
C	30.078340	28.468704	28.192924
C	30.367439	27.435453	29.054052
C	29.578103	26.268773	29.045360
C	28.515460	26.157222	28.176132
H	30.682687	29.375882	28.191686
H	31.205442	27.516369	29.743692
H	29.807897	25.452193	29.727299
H	27.903648	25.253986	28.168395
H	25.304772	27.323847	24.646127
C	22.524096	25.550253	23.163436
H	22.642447	26.519357	22.674472
H	22.626449	24.750696	22.417057
H	21.528343	25.493745	23.624093

SRG-OH S_1/S_0 torsion CI (PCM)

N	26.913978	25.818054	25.437239
N	26.000949	24.969502	25.178947
C	25.370519	24.169444	26.099969
C	26.778765	27.165851	25.484595
C	24.280163	23.388084	25.647371
C	23.621629	22.551953	26.539874
C	24.028748	22.491930	27.875120
C	25.091036	23.268806	28.327002
C	25.763973	24.102696	27.444557
O	23.960890	23.557287	24.343883
H	22.783331	21.945598	26.207483
H	23.504843	21.827941	28.559703
H	25.404307	23.220426	29.367722
H	26.608404	24.711107	27.767118
C	27.850666	27.952116	26.027959
C	27.695903	29.361360	26.123413
C	26.493801	29.958865	25.663262
C	25.495139	29.198120	25.110830
C	25.622219	27.801136	25.003999
H	26.380811	31.039665	25.735612
H	24.581298	29.648086	24.728249
O	24.623943	27.133941	24.392127
C	28.745948	30.128430	26.678335
C	29.900942	29.529341	27.121409
C	30.047577	28.130692	27.027961
C	29.041705	27.357708	26.494168
H	28.617417	31.208546	26.747619
H	30.701274	30.131032	27.547440
H	30.962867	27.658401	27.379754
H	29.154352	26.276486	26.423669
H	24.888641	26.207646	24.225727
C	22.881042	22.806395	23.824184
H	22.798566	23.080027	22.770274
H	23.070141	21.727398	23.906670
H	21.942157	23.052091	24.339017

SRG-OH S_0 cis minimum (PCM)

N	26.203148	26.380704	25.908714
N	25.117825	25.864086	26.248120
C	25.071069	24.537014	26.789011
C	27.409514	25.616836	25.838651
C	24.291424	23.576902	26.128727
C	24.102906	22.325469	26.704778
C	24.675150	22.049658	27.946245
C	25.444523	23.001366	28.605163
C	25.646820	24.247992	28.021097
O	23.834019	23.959786	24.905181
H	23.519176	21.562360	26.196899
H	24.516737	21.071062	28.394533
H	25.888252	22.778064	29.572560
H	26.236796	25.015260	28.521555
C	28.538917	26.126683	26.544661
C	29.805026	25.498604	26.383390
C	29.915534	24.404916	25.487342
C	28.829754	23.956728	24.790194
C	27.556413	24.553499	24.957403
H	30.886518	23.928822	25.355715
H	28.902794	23.127228	24.089799
O	26.582307	24.038233	24.177649
C	30.912959	25.979921	27.116958
C	30.781471	27.045856	27.974023
C	29.527055	27.670341	28.129724
C	28.427854	27.218721	27.437550
H	31.875571	25.484977	26.987664
H	31.640856	27.408602	28.534344
H	29.426649	28.515386	28.808457
H	27.459435	27.699176	27.568420
H	25.713553	24.457747	24.315538
C	23.228542	22.967556	24.092469
H	22.984620	23.455834	23.147211
H	23.925729	22.137598	23.911330
H	22.308652	22.585283	24.552524

DB366 S_0 trans minimum (QM/MM)

C	30.31256620	26.58146850	30.59675360
C	30.35562000	25.22210480	30.99580600
C	31.11312870	24.79242860	32.07888770
C	31.82554230	25.73859810	32.80132320
C	31.81449910	27.08153980	32.45300490
C	31.07675810	27.51099520	31.35262900
H	31.14921620	23.74200940	32.35344510
N	32.64536400	25.31323830	33.93233140
H	32.40106420	27.78932630	33.03320860
O	33.13232320	26.18335620	34.63702200
O	32.80040800	24.11287960	34.09748860
C	31.15988200	28.90866390	31.05998260
C	29.61776460	24.26064630	30.23576160
N	29.04281870	23.43831680	29.64958590
N	31.33860670	30.05411340	30.98219750
N	29.50700050	26.84256460	29.50745450
N	29.55826320	28.06934730	29.13237990
C	28.79144120	28.47573920	28.08962150
C	28.97565740	29.84604340	27.76970130
C	28.28470550	30.47516050	26.77405000
C	27.34491380	29.73871370	25.99631150
C	27.16095160	28.36148640	26.31301670
C	27.84330010	27.71665750	27.32241960
H	29.71643880	30.38361210	28.36009730
H	28.47994920	31.52253540	26.56318990
N	26.64662240	30.33146470	25.00177470
H	26.42053590	27.78135430	25.76671950
C	27.54024870	26.27831170	27.58774890
C	25.79227810	29.58134250	24.07984200
C	26.70575700	31.77485020	24.76302330
H	25.83460130	30.10090500	23.11285870
H	26.22784960	28.59155910	23.90569440
C	24.35766320	29.47995990	24.56302480
H	25.73039470	32.06739120	24.35096290
H	26.79887210	32.29906150	25.72082310
C	27.82277310	32.17225780	23.81760650
H	27.81471890	33.25618330	23.66447240
H	28.80320000	31.90048770	24.22549050
H	27.70969280	31.68625030	22.84028640
H	23.74100050	28.95795520	23.82280460
H	24.30270390	28.93142900	25.50995670
H	23.92448940	30.47515180	24.72424740
H	28.43282670	25.65200570	27.47438040
H	27.21128670	26.11436220	28.62112810
H	26.75946470	25.92279260	26.90940810

DB366 S_2 minimum (QM/MM)

C	30.474310	26.561462	30.576848
C	30.632724	25.205134	30.890904
C	31.459606	24.787225	31.935830
C	32.129699	25.752998	32.681981
C	31.992960	27.108079	32.390940
C	31.170299	27.517407	31.338726
H	31.584931	23.735211	32.170474
N	32.987807	25.351560	33.763298
H	32.548374	27.829886	32.983442
O	33.547359	26.245688	34.421051
O	33.121163	24.128920	33.964142
C	31.084968	28.929237	31.126131
C	29.960835	24.216701	30.104012
N	29.446787	23.384044	29.475457
N	31.100452	30.089955	31.097499
N	29.589805	26.833304	29.477541
N	29.704594	27.973533	29.006849
C	28.797174	28.321489	27.946376
C	28.948939	29.647000	27.533164
C	28.131521	30.211946	26.572827
C	27.108687	29.429637	25.984732
C	26.981058	28.083390	26.397234
C	27.793468	27.507609	27.373349
H	29.744168	30.221189	28.005456
H	28.296295	31.239641	26.259602
N	26.261483	29.961739	25.043679
H	26.197604	27.459732	25.973586
C	27.542213	26.080210	27.745700
C	25.376892	29.135646	24.230562
C	26.171211	31.397513	24.804864
H	25.315665	29.608563	23.240367
H	25.840676	28.156055	24.076202
C	23.982425	28.999952	24.826364
H	25.116643	31.616802	24.584207
H	26.406438	31.930834	25.732467
C	27.054919	31.863468	23.657355
H	26.909330	32.936014	23.492906
H	28.116194	31.697150	23.874250
H	26.802489	31.338614	22.728014
H	23.351183	28.399601	24.161125
H	24.024403	28.517839	25.808662
H	23.509381	29.982050	24.948863
H	28.445041	25.467380	27.654786
H	27.228230	25.975549	28.791543
H	26.759771	25.656381	27.110394

DB366 S_1 minimum (QM/MM)

C	30.516379	26.618030	30.556275
C	30.604558	25.226228	30.897471
C	31.409148	24.798003	31.931927
C	32.147428	25.732672	32.666799
C	32.093126	27.091622	32.362016
C	31.297530	27.549536	31.325391
H	31.484962	23.741009	32.169286
N	33.009294	25.286590	33.724481
H	32.692384	27.787419	32.942835
O	33.628223	26.133244	34.362769
O	33.085989	24.075170	33.929265
C	31.259557	28.948184	31.071403
C	29.897387	24.274797	30.106609
N	29.329150	23.524562	29.420485
N	31.244284	30.101382	30.920306
N	29.743924	26.969157	29.525500
N	29.486586	28.069934	29.024887
C	28.702651	28.470243	27.995180
C	28.788697	29.830570	27.625411
C	27.994771	30.352336	26.636966
C	27.048333	29.530588	25.963536
C	26.989309	28.161680	26.337995
C	27.780806	27.611693	27.323201
H	29.516580	30.449864	28.147383
H	28.113804	31.397962	26.362632
N	26.228906	30.033870	25.002074
H	26.260603	27.504240	25.869687
C	27.640234	26.173408	27.696124
C	25.371510	29.185822	24.176699
C	26.119355	31.467814	24.743408
H	25.305106	29.658733	23.187211
H	25.863692	28.221557	24.010109
C	23.984088	29.001138	24.765386
H	25.077367	31.660399	24.450989
H	26.271522	32.018709	25.680292
C	27.065394	31.947750	23.659698
H	26.915122	33.015312	23.466472
H	28.113440	31.804802	23.947939
H	26.889468	31.403758	22.723076
H	23.365005	28.383037	24.104330
H	24.039214	28.522718	25.749865
H	23.481255	29.968856	24.892901
H	28.590548	25.631804	27.611796
H	27.306284	26.047933	28.735528
H	26.906759	25.679337	27.054205

DB366 S_0 trans minimum (PCM)

C	30.292010	26.595580	30.601758
C	30.326045	25.234092	30.999412
C	31.099744	24.796624	32.064336
C	31.859003	25.728529	32.758112
C	31.856593	27.072702	32.410805
C	31.082828	27.517047	31.342946
H	31.116238	23.750249	32.353996
N	32.675125	25.287512	33.877691
H	32.460808	27.774609	32.979077
O	33.335892	26.128892	34.470767
O	32.655411	24.098379	34.164042
C	31.144148	28.922472	31.076190
C	29.539371	24.285531	30.272984
N	28.917740	23.478254	29.712661
N	31.305643	30.073237	31.004724
N	29.473413	26.864436	29.528325
N	29.518838	28.097147	29.162872
C	28.753010	28.505276	28.125334
C	28.910190	29.885519	27.829336
C	28.235839	30.506541	26.819309
C	27.333283	29.758618	26.009673
C	27.173706	28.374655	26.308466
C	27.841506	27.733773	27.325751
H	29.610784	30.441013	28.450815
H	28.412203	31.560673	26.630628
N	26.652271	30.337052	24.997554
H	26.473742	27.783793	25.723419
C	27.576215	26.281040	27.552473
C	25.800081	29.573571	24.084771
C	26.717333	31.775870	24.737422
H	25.812451	30.102929	23.123395
H	26.255950	28.595816	23.892800
C	24.377176	29.437520	24.596585
H	25.756841	32.060796	24.289227
H	26.773875	32.315237	25.689478
C	27.863782	32.147498	23.814163
H	27.862065	33.225564	23.617140
H	28.832654	31.879989	24.253710
H	27.775247	31.626021	22.852705
H	23.761054	28.891474	23.873240
H	24.346557	28.897692	25.551142
H	23.923249	30.424152	24.753737
H	28.493939	25.684993	27.480642
H	27.192920	26.096249	28.563579
H	26.848387	25.908008	26.822732

DB366 S_2/S_1 decay (QM/MM 0K dynamics)

C	30.43790520	26.62514610	30.62916530
C	30.56155750	25.26690120	30.92719460
C	31.39733560	24.83053800	31.95639800
C	32.10207500	25.78152920	32.68668550
C	31.98836430	27.14012050	32.40620750
C	31.15922800	27.57088960	31.37000450
H	31.51330150	23.77759760	32.19182050
N	32.98590480	25.35806180	33.73933850
H	32.56892010	27.84264050	32.99738650
O	33.58641100	26.24196810	34.38048020
O	33.10005440	24.12884980	33.92831070
C	31.10044080	28.98430420	31.15217850
C	29.86554210	24.30009050	30.13172820
N	29.33082110	23.50040380	29.47796920
N	31.13262130	30.14592970	31.10891570
N	29.53994820	26.92667910	29.53447270
N	29.70552330	28.03419830	29.02042100
C	28.76922910	28.38217290	27.96796030
C	28.88693950	29.70933910	27.57416670
C	28.06832770	30.25441690	26.60155040
C	27.08513850	29.44444790	25.99028100
C	26.99202320	28.09462380	26.39443230
C	27.80607820	27.53712880	27.38220590
H	29.65810360	30.30467970	28.06122070
H	28.21156330	31.28676490	26.29347270
N	26.23973280	29.95560360	25.02969280
H	26.23821340	27.44783310	25.95191360
C	27.59725820	26.09715470	27.73297110
C	25.39777400	29.10790050	24.19769170
C	26.11543260	31.38766050	24.79651140
H	25.33844240	29.58039930	23.20665270
H	25.89226770	28.14151590	24.04889210
C	23.99715060	28.92531220	24.76722060
H	25.06515750	31.58128940	24.53477180
H	26.29753580	31.92208750	25.73625390
C	27.02821980	31.89056360	23.68677560
H	26.86116720	32.96057650	23.52225760
H	28.08531600	31.75096070	23.93998830
H	26.82356520	31.36534100	22.74574830
H	23.39499440	28.30831120	24.08995530
H	24.03814590	28.44299670	25.74964510
H	23.49201500	29.89221500	24.88417580
H	28.52980990	25.52323650	27.69969130
H	27.21685170	25.97156890	28.75485200
H	26.87775220	25.64267650	27.04608280

DB366 S_1 minimum (PCM)

C	30.347933	26.594749	30.673483
C	30.302764	25.216824	31.071836
C	31.055652	24.761342	32.131300
C	31.878786	25.652746	32.832334
C	31.949852	27.000653	32.473317
C	31.205313	27.484422	31.414270
H	31.017801	23.717830	32.428982
N	32.657836	25.177358	33.930136
H	32.597442	27.667511	33.035360
O	33.375537	25.980353	34.530615
O	32.577330	23.983661	34.228346
C	31.298663	28.861601	31.070451
C	29.463516	24.317986	30.353031
N	28.772798	23.597417	29.754695
N	31.382197	29.990814	30.796530
N	29.599183	26.981879	29.642339
N	29.464984	28.097745	29.121944
C	28.740082	28.580360	28.092825
C	28.884495	29.957952	27.813186
C	28.194159	30.547562	26.789178
C	27.310438	29.784055	25.978157
C	27.178890	28.401803	26.278202
C	27.860497	27.781405	27.302107
H	29.567180	30.537657	28.431574
H	28.349531	31.604733	26.597858
N	26.618484	30.351971	24.956677
H	26.498067	27.786871	25.695283
C	27.672946	26.326348	27.575764
C	25.792384	29.566773	24.043122
C	26.651858	31.789950	24.702247
H	25.792758	30.093354	23.079713
H	26.275077	28.600715	23.853181
C	24.370296	29.389428	24.546412
H	25.688408	32.056631	24.248189
H	26.688879	32.328574	25.656497
C	27.795180	32.195979	23.788644
H	27.771160	33.275142	23.598157
H	28.767565	31.946932	24.231581
H	27.724080	31.678599	22.823360
H	23.773662	28.821957	23.822915
H	24.349666	28.853954	25.503828
H	23.886955	30.363247	24.696592
H	28.619369	25.773125	27.501010
H	27.289552	26.149383	28.590240
H	26.965961	25.887272	26.864457

DB366 S_0 cis minimum (PCM)

C	30.651105	26.544052	30.726997
C	30.414247	25.229559	31.232816
C	31.205193	24.672501	32.221853
C	32.280467	25.401923	32.719967
C	32.579578	26.668104	32.233256
C	31.784004	27.243134	31.250164
H	30.992379	23.681707	32.612720
N	33.110398	24.826950	33.756670
H	33.439758	27.205055	32.623292
O	34.048416	25.492875	34.177482
O	32.828928	23.704911	34.159449
C	32.173864	28.520858	30.749903
C	29.298222	24.498147	30.725671
N	28.386913	23.897530	30.323915
N	32.536522	29.554791	30.358721
N	29.918277	26.937586	29.654305
N	29.565690	28.115293	29.321472
C	29.296262	29.127962	30.188470
C	29.359515	30.405542	29.568707
C	29.279568	31.567126	30.277720
C	29.040008	31.524570	31.682127
C	28.801729	30.246371	32.263348
C	28.920094	29.064110	31.571203
H	29.546437	30.422595	28.496950
H	29.375455	32.514623	29.757409
N	28.991039	32.651541	32.421712
H	28.490659	30.183138	33.303253
C	28.512438	27.798871	32.264364
C	28.666391	32.640761	33.849171
C	29.292562	33.966810	31.853739
H	28.243509	33.625283	34.084577
H	27.863009	31.919571	34.037681
C	29.882661	32.362824	34.713957
H	29.720539	34.571160	32.663702
H	30.086281	33.865114	31.105217
C	28.062385	34.643955	31.277291
H	28.318638	35.635909	30.888368
H	27.630333	34.054753	30.458837
H	27.289337	34.768992	32.046033
H	29.613008	32.394351	35.775625
H	30.307931	31.375132	34.496480
H	30.664301	33.112587	34.537757
H	28.088962	27.066272	31.566455
H	29.349523	27.315856	32.787679
H	27.751922	28.021513	33.020903

DB165-C1 S_0 trans minimum (QM/MM)

C	23.694210	26.570456	30.451276
C	26.827705	30.412223	30.509781
H	28.621646	29.722663	28.626290
C	30.521851	31.328438	28.268214
H	29.853912	31.444783	29.126330
H	31.563092	31.308787	28.606364
H	30.405909	32.188865	27.598271
C	29.003221	24.427069	24.150618
H	28.813662	23.796590	25.028280
H	29.901560	24.009293	23.675837
C	27.826459	24.401482	23.190298
H	27.610294	23.377913	22.863283
H	26.919675	24.803440	23.659319
H	28.039569	25.006390	22.300475
C	30.329400	26.492375	23.783781
H	30.031337	27.544211	23.704661
H	30.260569	26.076041	22.771816
C	31.750816	26.362492	24.300644
H	31.857327	26.790247	25.305240
H	32.051458	25.308310	24.339759
H	32.441235	26.885512	23.629658
C	28.769603	26.347771	25.683650
C	29.243518	27.586969	26.173215
H	30.095728	28.065012	25.717768
C	28.675314	28.188815	27.278128
C	27.542559	27.579672	27.912467
C	27.088008	26.328866	27.422111
H	26.224863	25.887344	27.913728
C	27.678787	25.716884	26.358974
H	27.278074	24.775898	25.994807
C	30.268593	30.088604	27.461544
C	25.276306	28.454720	30.407449
C	24.089237	27.864920	30.914681
C	23.295569	28.506640	31.852539
H	22.388264	28.048152	32.231750
C	23.685740	29.752089	32.317496
C	24.851626	30.359261	31.878127
H	25.130970	31.333166	32.269896
C	25.648296	29.723447	30.927445
N	22.851308	30.431993	33.298942
N	29.344241	25.777611	24.598066
N	29.149020	29.377507	27.823324
N	26.950937	28.259141	28.922957
N	25.914616	27.706842	29.446392
N	27.760610	31.050461	30.234396
N	23.326503	25.516205	30.127470
O	23.237181	31.507443	33.727069
O	21.801038	29.894810	33.621375
O	31.013551	29.770136	26.542629

DB165-C1 S_2 minimum (QM/MM)

C	23.762011	26.959470	30.618411
C	26.972494	30.730314	30.802899
H	28.708172	30.033832	28.797882
C	30.608399	31.611307	28.462703
H	29.708945	32.015925	28.936750
H	31.298243	31.301090	29.259024
H	31.100774	32.376440	27.856110
C	29.156483	24.571447	24.532825
H	28.980079	23.988753	25.446303
H	30.051737	24.146934	24.057318
C	27.970438	24.476519	23.581988
H	27.766727	23.431090	23.322823
H	27.064715	24.899676	24.031515
H	28.177235	25.019205	22.652857
C	30.468363	26.613588	24.054460
H	30.205894	27.675293	23.977785
H	30.366619	26.188943	23.047060
C	31.899341	26.438412	24.543899
H	32.015987	26.803096	25.571554
H	32.196979	25.383235	24.509419
H	32.583606	26.995535	23.894244
C	28.892452	26.572702	25.962071
C	29.368839	27.826298	26.401252
H	30.237559	28.285857	25.951660
C	28.767529	28.483706	27.469110
C	27.628842	27.902760	28.091186
C	27.171993	26.647754	27.666909
H	26.301979	26.222212	28.161703
C	27.786796	25.980455	26.633242
H	27.381178	25.026984	26.309860
C	30.320788	30.421082	27.594899
C	25.338383	28.843963	30.599961
C	24.141994	28.257983	31.080092
C	23.326060	28.911895	31.990986
H	22.406974	28.463688	32.349126
C	23.716549	30.165907	32.456554
C	24.915601	30.753591	32.057505
H	25.195710	31.720145	32.466524
C	25.733971	30.103088	31.138146
N	22.860764	30.876073	33.375014
N	29.487097	25.942526	24.901122
N	29.221829	29.692804	27.984075
N	27.017034	28.665413	29.106651
N	25.994695	28.112466	29.618499
N	27.975899	31.286909	30.601862
N	23.426745	25.902414	30.268454
O	23.214698	31.991340	33.751103
O	21.803377	30.330609	33.712767
O	31.031128	30.115760	26.645583

DB165-C1 S_1 minimum (QM/MM)

C	23.843536	26.931722	30.656438
C	26.932545	30.761546	30.806540
H	28.844593	30.016629	28.814255
C	30.625532	31.650167	28.413409
H	29.694202	32.185939	28.624202
H	31.047929	31.331681	29.375445
H	31.334501	32.307543	27.904711
C	29.282599	24.572087	24.504694
H	29.093606	23.980212	25.410132
H	30.182755	24.142577	24.042118
C	28.114093	24.489868	23.538094
H	27.910857	23.449774	23.256862
H	27.199658	24.904133	23.980037
H	28.330024	25.050595	22.621689
C	30.605882	26.617000	24.061468
H	30.343489	27.679444	23.989844
H	30.508720	26.206828	23.048061
C	32.031161	26.439937	24.554954
H	32.142261	26.791092	25.588131
H	32.328929	25.384525	24.515776
H	32.725014	27.001938	23.919542
C	29.007878	26.558028	25.944741
C	29.468772	27.814419	26.400891
H	30.339649	28.278447	25.960321
C	28.864316	28.469120	27.457013
C	27.719420	27.877482	28.074387
C	27.270732	26.610246	27.633904
H	26.396810	26.173542	28.113020
C	27.897684	25.962149	26.606974
H	27.496271	25.009019	26.276960
C	30.377566	30.445342	27.551350
C	25.346201	28.880656	30.628814
C	24.156761	28.242514	31.116580
C	23.336215	28.881008	32.019284
H	22.435467	28.399183	32.382836
C	23.671873	30.156712	32.482125
C	24.850870	30.783593	32.083390
H	25.098059	31.760409	32.488388
C	25.699123	30.164597	31.180003
N	22.785893	30.837385	33.384316
N	29.615453	25.938130	24.895067
N	29.309950	29.683856	27.970237
N	27.080147	28.555012	29.052936
N	26.052757	28.259706	29.688122
N	27.974796	31.173471	30.487849
N	23.618920	25.860571	30.260409
O	23.090937	31.962694	33.757498
O	21.749890	30.262313	33.720255
O	31.071734	30.165630	26.584685

DB165-C1 S_2/S_1 decay (QM/MM 0K dynamics)

C	23.81897040	26.99155580	30.67211530
C	27.01716590	30.77572990	30.73287810
H	28.75490960	29.99558360	28.79003310
C	30.61254900	31.59826080	28.43205990
H	29.66274980	32.06314010	28.71820330
H	31.10229240	31.26304600	29.35753390
H	31.25985730	32.30557030	27.90925080
C	29.41066810	24.44747620	24.63487920
H	29.25807430	23.83211090	25.53714080
H	30.27582420	24.01263120	24.11553660
C	28.20553770	24.37408090	23.71285740
H	27.99286440	23.32883560	23.46081650
H	27.29953000	24.78915560	24.17040540
H	28.38782450	24.91773010	22.77901480
C	30.83543520	26.44076980	24.26793030
H	30.61192090	27.51346950	24.18210380
H	30.75973750	26.04506570	23.24710890
C	32.24648920	26.22516790	24.77884410
H	32.36782060	26.54017050	25.82427340
H	32.53838960	25.16965950	24.71573940
H	32.94678720	26.79520110	24.15764270
C	29.12469300	26.40652530	26.06479950
C	29.62022220	27.65931290	26.57301670
H	30.55886210	28.04553480	26.19755150
C	28.91016230	28.38518510	27.58251170
C	27.75811940	27.82598780	28.16626350
C	27.33708030	26.60003110	27.69250410
H	26.42575500	26.21856770	28.15261700
C	27.98117390	25.90662240	26.68112240
H	27.59498720	24.94546750	26.36733940
C	30.38152900	30.40866370	27.56705310
C	25.40165390	28.88222990	30.60560430
C	24.20456950	28.29503460	31.10788100
C	23.39992950	28.94540500	32.02322330
H	22.48266600	28.51678070	32.41171620
C	23.80521330	30.20403890	32.47308890
C	24.98172000	30.80545570	32.05731420
H	25.26066470	31.77911910	32.45120690
C	25.80406770	30.15826050	31.13082270
N	22.97135960	30.91687400	33.42109900
N	29.79133750	25.78699560	25.04810950
N	29.32298740	29.59947380	28.04169840
N	27.13477720	28.62508710	29.16229620
N	26.05359680	28.14832560	29.65238340
N	28.00948190	31.33026280	30.46611640
N	23.50277650	25.92868930	30.32105830
O	23.32761910	32.01774640	33.82100420
O	21.91453880	30.39087570	33.78910640
O	31.02434710	30.10789030	26.58146700

DB165-C1 S_0 trans minimum (PCM)

C	23.465926	26.770538	30.229008
C	26.931999	30.308623	30.569610
H	28.459495	29.811545	28.485463
C	30.240102	31.500241	28.100453
H	30.237656	31.312141	29.179799
H	31.167570	31.996895	27.807181
H	29.391921	32.164659	27.892539
C	29.021216	24.406169	24.167260
H	28.712579	23.793831	25.021265
H	29.936788	23.939979	23.781629
C	27.952126	24.448101	23.090516
H	27.726038	23.435474	22.737984
H	27.024403	24.896384	23.467317
H	28.286842	25.040930	22.229971
C	30.512904	26.365790	23.921555
H	30.321608	27.439727	23.818592
H	30.503268	25.957559	22.903060
C	31.850348	26.096900	24.587978
H	31.884413	26.518443	25.600080
H	32.039128	25.018611	24.663819
H	32.662384	26.543756	24.003420
C	28.762508	26.360198	25.652259
C	29.228676	27.604179	26.144091
H	30.112406	28.067106	25.730599
C	28.590998	28.247671	27.185291
C	27.420364	27.655295	27.780338
C	26.963724	26.406753	27.271971
H	26.074125	25.976442	27.724730
C	27.600335	25.771378	26.255185
H	27.205676	24.829447	25.888205
C	30.104038	30.221972	27.322129
C	25.215851	28.491013	30.323842
C	23.999178	27.953751	30.829742
C	23.314043	28.541001	31.880754
H	22.385300	28.116875	32.250776
C	23.836974	29.690894	32.459595
C	25.022573	30.254000	32.008338
H	25.406970	31.150846	32.486435
C	25.714411	29.668631	30.952595
N	23.130912	30.315565	33.563470
N	29.395694	25.742911	24.635321
N	29.019963	29.454593	27.711055
N	26.829199	28.326748	28.781266
N	25.767570	27.788432	29.290842
N	27.901906	30.919478	30.363783
N	22.996432	25.810452	29.771048
O	23.612902	31.328497	34.052713
O	22.091445	29.793817	33.944134
O	30.872085	29.903719	26.429586

DB165-C1 S_1 minimum (PCM)

C	25.282512	28.545576	30.334380
N	26.010370	28.069950	29.334469
N	27.019201	28.489895	28.742993
C	27.705587	27.943638	27.720592
C	27.316040	26.712432	27.140759
C	28.024224	26.168945	26.106579
C	29.170384	26.836681	25.584612
C	29.553269	28.070809	26.162818
C	28.852279	28.628655	27.214846
C	25.559340	29.788510	31.009327
C	24.746429	30.206876	32.046475
C	23.656468	29.431984	32.444646
C	23.358467	28.218708	31.809189
C	24.151273	27.777860	30.774067
C	23.870619	26.548722	30.111129
N	23.671611	25.550811	29.546639
C	26.665984	30.579700	30.604074
N	27.579946	31.209862	30.250160
N	22.829362	29.886409	33.519120
O	23.111230	30.955735	34.061591
O	21.872311	29.186350	33.852179
H	22.503621	27.635986	32.139086
H	24.950840	31.143072	32.558146
H	26.433340	26.213777	27.537201
H	27.682376	25.231822	25.678498
N	29.880810	26.307777	24.555576
C	29.595060	24.982888	24.010208
C	30.983019	27.030706	23.924799
C	28.562752	25.018868	22.896872
C	32.308811	26.818091	24.635139
H	27.602325	25.408638	23.256867
H	28.898996	25.663185	22.074573
H	28.395875	24.013223	22.494039
H	29.287860	24.312743	24.821577
H	30.545643	24.579623	23.636802
H	31.045297	26.674278	22.888434
H	30.729219	28.095658	23.860649
H	32.562464	25.750994	24.673560
H	33.114473	27.340388	24.106421
H	32.275513	27.194676	25.665180
H	30.426185	28.601644	25.810748
N	29.202825	29.829215	27.824906
C	30.304827	30.624014	27.570083
C	30.346997	31.869169	28.412151
H	28.585796	30.161785	28.565312
O	31.154869	30.355335	26.737233
H	31.314671	32.356916	28.275535
H	30.184649	31.646915	29.472800
H	29.550925	32.559103	28.104379

DB165-C1 S_0 cis minimum (PCM)

C	27.326648	28.400538	27.762539
N	27.213328	28.567631	29.097847
N	26.305421	28.032900	29.820042
C	25.033638	27.730505	29.435825
C	24.417332	26.574460	29.995590
C	23.073848	26.298584	29.797130
C	22.301440	27.184917	29.055051
C	22.846360	28.345208	28.517207
C	24.193713	28.622238	28.696438
C	28.256757	29.278537	27.099561
C	28.433150	29.200279	25.734430
C	27.748578	28.227434	24.964808
C	26.887019	27.301918	25.641009
C	26.682305	27.403729	26.981668
C	24.714695	29.844696	28.177706
N	25.133978	30.846713	27.760965
C	25.222790	25.670825	30.752671
N	25.868022	24.927259	31.371579
N	20.897049	26.899773	28.846098
O	20.441314	25.873803	29.335364
O	20.240797	27.699024	28.190091
H	22.624036	25.400186	30.209900
H	22.213100	29.031582	27.962079
N	28.900237	30.200825	27.903895
C	29.827455	31.162912	27.552534
C	30.316073	31.964920	28.727143
O	30.207144	31.348041	26.409044
H	28.655822	30.116674	28.888865
H	29.484324	32.494670	29.207570
H	31.054567	32.691807	28.382456
N	27.916603	28.160878	23.629149
C	28.726196	29.138430	22.898319
C	30.192686	28.749970	22.842891
C	27.298038	27.117748	22.807430
C	25.894506	27.488268	22.364229
H	28.587412	30.129169	23.345169
H	28.306621	29.202294	21.886459
H	30.630028	28.699598	23.847659
H	30.759265	29.484116	22.259086
H	30.318561	27.768016	22.369515
H	27.313425	26.166115	23.349873
H	27.951104	26.974018	21.937598
H	25.476610	26.703845	21.723237
H	25.227323	27.620747	23.225057
H	25.900389	28.426346	21.795043
H	26.390708	26.508004	25.093125
H	26.047673	26.669457	27.472145
H	29.124180	29.893078	25.274938
H	30.772934	31.314432	29.482821

DB165-C2 S_0 trans minimum (QM/MM)

C	26.328518	32.045395	30.815930
C	24.606031	27.391456	31.105863
H	28.223858	30.169030	29.306073
C	29.932029	31.953671	28.957030
H	28.992353	32.503456	28.820676
H	30.037874	31.772636	30.032062
H	30.772060	32.555434	28.601685
C	29.348098	24.764707	25.072265
H	29.184908	24.090475	25.918288
H	30.275969	24.420002	24.601834
C	28.199650	24.713033	24.080078
H	28.068959	23.698057	23.688061
H	27.251341	25.005678	24.546005
H	28.384649	25.383620	23.230225
C	30.647734	26.859080	24.819497
H	30.366102	27.914351	24.733387
H	30.646206	26.470458	23.793036
C	32.018568	26.702126	25.450558
H	32.038390	27.162722	26.445992
H	32.275535	25.640955	25.566077
H	32.789910	27.173473	24.834362
C	28.970252	26.647867	26.618080
C	29.281286	27.952382	27.085315
H	30.102257	28.508497	26.654673
C	28.567151	28.556871	28.104958
C	27.468379	27.839529	28.706921
C	27.237383	26.503638	28.280920
H	26.423762	25.979085	28.778972
C	27.945441	25.909874	27.283248
H	27.701008	24.898923	26.981376
C	29.901518	30.655595	28.210893
C	25.544997	29.728899	30.926372
C	25.417213	31.077649	31.343311
C	24.409074	31.485096	32.207974
H	24.311678	32.522793	32.512246
C	23.527832	30.531120	32.693329
C	23.633283	29.195084	32.340033
H	22.942200	28.468869	32.752917
C	24.617293	28.784159	31.444465
N	22.445656	30.947311	33.580306
N	29.615625	26.122736	25.553985
N	28.858462	29.829557	28.576573
N	26.565521	28.261095	29.612296
N	26.570675	29.477215	30.033465
N	24.473899	26.250744	30.918858
N	27.048974	32.849532	30.384834
O	21.780560	30.075566	34.117058
O	22.268201	32.147339	33.723397
O	30.727222	30.353019	27.358662

DB165-C2 S_2 minimum (QM/MM)

C	26.322847	31.922525	30.833849
C	24.675343	27.232251	30.981743
H	28.112039	30.233567	29.088343
C	29.832045	32.027409	28.888612
H	28.920191	32.605905	28.695196
H	29.843676	31.814889	29.963836
H	30.712921	32.617528	28.624305
C	29.735550	24.851953	24.980746
H	29.470397	24.225277	25.837269
H	30.743376	24.533054	24.689531
C	28.759088	24.672709	23.826616
H	28.743649	23.627629	23.496345
H	27.736359	24.942363	24.119227
H	29.044301	25.294954	22.968876
C	30.919382	27.008521	24.749170
H	30.589960	28.039967	24.580691
H	31.064314	26.571229	23.753233
C	32.225894	26.966110	25.530112
H	32.134836	27.517902	26.474097
H	32.505196	25.930658	25.760308
H	33.029875	27.408375	24.935512
C	29.078587	26.777585	26.385913
C	29.310678	28.080653	26.867300
H	30.137583	28.667115	26.493830
C	28.522133	28.652180	27.865129
C	27.428238	27.891444	28.391833
C	27.213628	26.594781	27.906532
H	26.374074	26.052340	28.339128
C	28.002497	26.024930	26.932943
H	27.775322	25.027765	26.578186
C	29.837658	30.757755	28.094304
C	25.559823	29.596019	30.866633
C	25.461260	30.917942	31.371730
C	24.515705	31.264232	32.326130
H	24.438712	32.276257	32.709026
C	23.661771	30.270708	32.808644
C	23.743038	28.955025	32.357474
H	23.078068	28.204687	32.770134
C	24.669791	28.605133	31.382376
N	22.667185	30.614183	33.793615
N	29.859103	26.244237	25.399537
N	28.774730	29.922532	28.373373
N	26.475952	28.230333	29.365598
N	26.545509	29.376568	29.909958
N	24.588749	26.097175	30.737692
N	27.007921	32.745874	30.380919
O	21.948677	29.713551	34.234141
O	22.587937	31.796408	34.135909
O	30.706937	30.487891	27.273117

DB165-C2 S_1 minimum (QM/MM)

C	26.280527	32.021511	30.887602
C	24.667443	27.370731	30.902638
H	28.147995	30.324796	29.024593
C	29.880632	32.057213	28.851794
H	28.959443	32.628856	28.681726
H	29.928326	31.845084	29.926459
H	30.749298	32.653884	28.563027
C	29.669689	24.835399	25.000001
H	29.397781	24.234993	25.874994
H	30.671043	24.488648	24.717084
C	28.694064	24.638758	23.852774
H	28.663326	23.588350	23.538802
H	27.673342	24.929798	24.132770
H	28.987632	25.239830	22.981614
C	30.903994	26.965666	24.743913
H	30.599151	28.002032	24.554598
H	31.043940	26.517655	23.751465
C	32.201768	26.906610	25.531251
H	32.116258	27.463009	26.474127
H	32.459694	25.866568	25.770328
H	33.024255	27.329350	24.946941
C	29.036754	26.791491	26.355077
C	29.295883	28.098887	26.830379
H	30.139032	28.663092	26.456547
C	28.528205	28.697049	27.816133
C	27.409022	27.969890	28.341914
C	27.146801	26.667575	27.853545
H	26.286740	26.145187	28.269204
C	27.934231	26.081501	26.903528
H	27.681432	25.085079	26.555943
C	29.879325	30.782665	28.063392
C	25.494739	29.711719	30.887803
C	25.410650	31.031811	31.430792
C	24.493526	31.331975	32.417999
H	24.428677	32.331691	32.835522
C	23.646437	30.327260	32.893275
C	23.706094	29.028057	32.397411
H	23.048377	28.265708	32.799078
C	24.608868	28.703031	31.398501
N	22.683941	30.643656	33.919898
N	29.814794	26.235268	25.389113
N	28.796788	29.966473	28.328160
N	26.586011	28.413969	29.303997
N	26.374468	29.488684	29.899717
N	24.737312	26.286342	30.483559
N	27.005382	32.807392	30.427561
O	21.968421	29.739915	34.340683
O	22.634056	31.803884	34.316929
O	30.754619	30.483451	27.262995

DB165-C2 S_2/S_1 decay (QM/MM 0K dynamics)

C	26.33793360	31.95492670	30.85781430
C	24.86576120	27.24913270	30.99591650
H	28.23754220	30.29808610	29.12903110
C	29.93656070	32.10262130	28.93154320
H	28.98758020	32.64336750	28.84424820
H	30.06628830	31.91000740	30.00376540
H	30.74985490	32.75322020	28.60495760
C	29.91293960	24.91278470	25.07454780
H	29.55976780	24.31831450	25.92179010
H	30.90931170	24.51672460	24.85933130
C	29.03055490	24.71687430	23.83929420
H	29.05442760	23.65753230	23.55359010
H	27.98659630	24.98474480	24.04516400
H	29.38189320	25.31154520	22.98654450
C	31.15054300	27.02831860	24.89526000
H	30.85511840	28.06785470	24.72809370
H	31.34334830	26.62029320	23.89471690
C	32.42783480	26.95107690	25.73182280
H	32.30731500	27.50065780	26.67537660
H	32.66162020	25.90427630	25.96902080
H	33.26194520	27.38353440	25.16984060
C	29.17879460	26.87026490	26.38091700
C	29.45794150	28.15114380	26.92012110
H	30.34594820	28.65851140	26.57927060
C	28.61040380	28.75334280	27.82730170
C	27.42820700	28.04157970	28.26087170
C	27.19757900	26.77453180	27.72112780
H	26.29914390	26.26662140	28.07713820
C	28.03335330	26.15880000	26.80885890
H	27.78937800	25.16825190	26.44535330
C	29.93130230	30.84008800	28.12925900
C	25.59302830	29.65601870	30.85827140
C	25.48985470	30.94712330	31.37613840
C	24.52275090	31.24219570	32.34296480
H	24.45286070	32.24842920	32.73706850
C	23.68273300	30.26115140	32.82052980
C	23.80305980	28.95532520	32.37176190
H	23.20184880	28.14666930	32.76749690
C	24.75035130	28.64565520	31.40344760
N	22.65003170	30.55472240	33.80979150
N	30.04187720	26.29779540	25.50197850
N	28.87670030	30.01267680	28.38272180
N	26.45888960	28.41007370	29.18330830
N	26.65416000	29.42804870	29.93188330
N	24.86165830	26.10865750	30.72063550
N	27.02897640	32.76008220	30.38111100
O	21.94940690	29.60301510	34.20142060
O	22.57046170	31.73842240	34.17555530
O	30.79675060	30.58210830	27.30473550

DB165-C2 S_0 trans minimum (PCM)

C	26.191617	32.091632	30.607305
C	24.688218	27.400806	31.249652
H	27.990037	30.206156	29.099515
C	29.839568	31.868621	29.013066
H	28.969057	32.492869	28.775992
H	29.792432	31.655483	30.088675
H	30.758266	32.416165	28.791577
C	29.444085	24.714524	25.108946
H	29.125567	24.101965	25.959391
H	30.435322	24.338647	24.824724
C	28.477478	24.601919	23.944030
H	28.389899	23.558819	23.619742
H	27.478459	24.961467	24.220237
H	28.824311	25.195875	23.089042
C	30.729545	26.822143	24.907904
H	30.423565	27.858550	24.725548
H	30.868595	26.369966	23.917982
C	32.018341	26.752468	25.707763
H	31.908733	27.235744	26.686675
H	32.317152	25.709946	25.875241
H	32.827965	27.255950	25.167447
C	28.844901	26.669423	26.485656
C	29.133382	27.973792	26.961628
H	29.995943	28.512314	26.597168
C	28.367807	28.590367	27.930014
C	27.209237	27.899144	28.457333
C	26.940598	26.591500	27.958942
H	26.065121	26.093344	28.371187
C	27.710869	25.980942	27.020611
H	27.441976	24.990026	26.670151
C	29.820821	30.593382	28.218632
C	25.487942	29.757320	30.854467
C	25.375739	31.114360	31.256517
C	24.484223	31.520091	32.237991
H	24.405431	32.563788	32.527207
C	23.684472	30.562097	32.845719
C	23.762503	29.221654	32.493869
H	23.129428	28.496881	32.998485
C	24.653593	28.808201	31.508185
N	22.747946	30.970727	33.879458
N	29.628984	26.098681	25.549428
N	28.676438	29.842956	28.432418
N	26.324534	28.311297	29.377739
N	26.420698	29.500347	29.882600
N	24.613504	26.241744	31.173123
N	26.834418	32.912114	30.091611
O	22.055406	30.106296	34.398445
O	22.704655	32.157607	34.172336
O	30.726763	30.259750	27.473049

DB165-C2 S_1 minimum (PCM)

C	26.215130	32.071917	30.726825
C	24.468593	27.461785	31.113859
H	27.918205	30.321724	28.910256
C	29.829804	31.875531	28.965343
H	28.970921	32.494969	28.681710
H	29.757460	31.709224	30.047921
H	30.758784	32.405938	28.745415
C	29.531213	24.687680	25.128940
H	29.273395	24.091116	26.011901
H	30.528140	24.346132	24.820663
C	28.529994	24.480714	24.005998
H	28.479112	23.422337	23.725493
H	27.525398	24.806110	24.304056
H	28.818718	25.055201	23.116574
C	30.706980	26.847261	24.824481
H	30.340983	27.858359	24.607697
H	30.855873	26.367443	23.848691
C	32.013372	26.884702	25.599159
H	31.892522	27.395949	26.562645
H	32.376007	25.867951	25.797393
H	32.782652	27.415058	25.026041
C	28.875602	26.656306	26.462920
C	29.109766	27.984698	26.890964
H	29.929359	28.559116	26.483040
C	28.355080	28.591960	27.875626
C	27.257971	27.860833	28.443980
C	27.019425	26.535232	28.008029
H	26.184419	26.002921	28.458518
C	27.797207	25.940996	27.055790
H	27.561050	24.928249	26.745407
C	29.831295	30.563795	28.233756
C	25.400138	29.763507	30.906218
C	25.364060	31.122720	31.359749
C	24.509567	31.514777	32.367020
H	24.487521	32.546330	32.705102
C	23.657211	30.572732	32.954657
C	23.652275	29.242814	32.531769
H	22.976640	28.536146	33.005614
C	24.500825	28.824050	31.522729
N	22.772031	30.980102	34.002444
N	29.662736	26.091055	25.512616
N	28.632111	29.874082	28.341905
N	26.442918	28.332986	29.398810
N	26.237723	29.448232	29.916061
N	24.424554	26.343127	30.791165
N	26.909828	32.829967	30.181420
O	22.028719	30.134202	34.501080
O	22.796304	32.159044	34.357580
O	30.795953	30.142776	27.619390

DB165-C2 S_0 cis minimum (PCM)

C	26.774226	28.144778	27.776161
N	25.698980	28.700494	28.410759
N	25.768039	29.659599	29.245861
C	26.857871	30.025956	29.993800
C	27.057051	31.414186	30.228278
C	28.041132	31.877618	31.088835
C	28.835001	30.956283	31.758969
C	28.647322	29.589337	31.603573
C	27.671166	29.118795	30.733209
N	25.628760	33.110644	28.888557
C	26.256068	32.346397	29.501265
H	28.199714	32.942696	31.232692
N	29.875203	31.432688	32.652680
O	30.012916	32.642248	32.774052
O	30.557187	30.598514	33.231828
H	29.264685	28.892719	32.163815
C	27.477131	27.706608	30.653081
N	27.334256	26.552632	30.634153
C	28.060791	28.713186	27.510641
C	29.086595	27.902630	27.091297
C	28.909635	26.503265	26.907160
C	27.582585	25.989263	26.980364
C	26.557737	26.805719	27.367192
H	25.545677	26.418654	27.466958
H	27.372039	24.954228	26.731262
N	29.964405	25.712744	26.619099
C	29.833626	24.261780	26.484460
H	30.777497	23.823953	26.834296
H	29.063280	23.903133	27.176358
C	29.543883	23.836494	25.056587
H	30.338116	24.175833	24.379583
H	29.480833	22.744645	24.986863
H	28.595781	24.259365	24.701383
C	31.319597	26.249940	26.477746
H	31.291215	27.162776	25.870384
H	31.887913	25.519048	25.890052
C	31.990505	26.493461	27.818076
H	32.022083	25.568888	28.408347
H	33.019468	26.840793	27.671052
H	31.454316	27.252940	28.401465
H	30.044051	28.365665	26.888956
N	28.227733	30.106223	27.563999
H	27.429565	30.666131	27.281710
C	29.388116	30.774440	27.904312
O	30.379034	30.202393	28.326094
C	29.336915	32.262984	27.699167
H	29.876025	32.505981	26.775189
H	29.862578	32.753557	28.524450
H	28.322992	32.669201	27.615422

REFERENCES

(1) *Cobramm is Optimized in Bologna to Run Ab initio Molecular Mechanics*. https://gitlab.com/cobrammgroup/cobramm.

(2) Wang, J.; Wolf, R. M.; Caldwell, J. W.; Kollman, P. A.; Case, D. A. Development and Testing of a General Amber Force Field. *J. Comput. Chem.* **2004**, *25* (9).

(3) Case, D. A.; Ben-Shalom, I. Y.; Brozell, S. R.; Cerutti, D. S.; Cheatham, T. E.; Cruzeiro, V. W. D.; Darden, T. A.; Duke, R. E.; Ghoreishi, D.; Gilson, M. K. AMBER 2018.

(4) Aleotti, F.; Soprani, L.; Nenov, A.; Berardi, R.; Arcioni, A.; Zannoni, C.; Garavelli, M. Multidimensional Potential Energy Surfaces Resolved at the RASPT2 Level for Accurate Photoinduced Isomerization Dynamics of Azobenzene. *J. Chem. Theory Comput.* **2019**, *15* (12), 6813–6823.

(5) Aleotti, F.; Nenov, A.; Salvigni, L.; Bonfanti, M.; El-Tahawy, M. M.; Giunchi, A.; Gentile, M.; Spallacci, C.; Ventimiglia, A.; Cirillo, G.; Montali, L.; Scurti, S.; Garavelli, M.; Conti, I. Spectral Tuning and Photoisomerization Efficiency in Push–Pull Azobenzenes: Designing Principles. *J. Phys. Chem. A* **2020**, *124* (46), 9513–9523.

(6) Ciminelli, C.; Granucci, G.; Persico, M. The Photoisomerization Mechanism of Azobenzene: A Semiclassical Simulation of Nonadiabatic Dynamics. *Chem. – Eur. J.* **2004**, *10* (9), 2327–2341.

(7) Conti, I.; Garavelli, M.; Orlandi, G. The Different Photoisomerization Efficiency of Azobenzene in the Lowest Nπ* and Ππ* Singlets: The Role of a Phantom State. *J. Am. Chem. Soc.* **2008**, *130* (15), 5216–5230.

(8) Josep Casellas; Michael J. Bearpark; Mar Reguero. Excited State Decay in the Photoisomerization of Azobenzene: A New Balance between Mechanisms. *ChemPhysChem* **2016**.

(9) Nenov, A.; Borrego-Varillas, R.; Oriana, A.; Ganzer, L.; Segatta, F.; Conti, I.; Segarra-Marti, J.; Omachi, J.; Dapor, M.; Taioli, S.; Manzoni, C.; Mukamel, S.; Cerullo, G.; Garavelli, M. UV-Light-Induced Vibrational Coherences: The Key to Understand Kasha Rule Violation in Trans-Azobenzene. *J. Phys. Chem. Lett.* **2018**, *9* (7), 1534–1541.

(10) Magde, D.; Rojas, G. E.; Seybold, P. G. Solvent Dependence of the Fluorescence Lifetimes of Xanthene Dyes. *Photochem. Photobiol.* **1999**, *70* (5), 737–744.

(11) Fakis, M.; Petropoulos, V.; Hrobárik, P.; Nociarová, J.; Osuský, P.; Maiuri, M.; Cerullo, G. Exploring Solvent and Substituent Effects on the Excited State Dynamics and Symmetry Breaking of Quadrupolar Triarylamine End-Capped Benzothiazole Chromophores by Femtosecond Spectroscopy. *J. Phys. Chem. B* **2022**, *126* (42), 8532–8543.
